# Supplementary material for: Trial protocol: a multicentre randomised trial of first-line treatment pathways for newly diagnosed immune thrombocytopenia: standard steroid treatment versus combined steroid and mycophenolate. The FLIGHT trial
Source: BMJ Open. 2018 Oct 18;8(10):e024427. doi: 10.1136/bmjopen-2018-024427 (PMC6196935; doi:10.1136/bmjopen-2018-024427)
Supplement: Author's manuscript [file bmjopen-2018-024427.draft_revisions.pdf]

BMJ Open

BMJ Open is committed to open peer review. As part of this commitment we make the peer review history of every article we publish publicly available.

When an article is published we post the peer reviewers' comments and the authors' responses online. We also post the versions of the paper that were used during peer review. These are the versions that the peer review comments apply to.

The versions of the paper that follow are the versions that were submitted during the peer review process. They are not the versions of record or the final published versions. They should not be cited or distributed as the published version of this manuscript.

BMJ Open is an open access journal and the full, final, typeset and author-corrected version of record of the manuscript is available on our site with no access controls, subscription charges or pay-per-view fees (http://bmjopen.bmj.com).

If you have any questions on BMJ Open's open peer review process please email info.bmjopen@bmj.com

BMJ Open

A multicentre randomised trial of First Line treatment pathways for newly diagnosed Immune Thrombocytopenia: Standard steroid treatment versus combined steroid and mycophenolate. The FLIGHT trial.

Journal:	*BMJ Open*
Manuscript ID	bmjopen-2018-024427
Article Type:	Protocol
Date Submitted by the Author:	25-May-2018

Complete List of Authors:	Pell, Julie; Cardiff University Centre for Trials Research<br>Greenwood, Rosemary; University Hospitals Bristol NHS Foundation Trust, Research and Design Service<br>Ingram, Jenny; Bristol University , School of Social & Community Medicine<br>Wale, Katharine; Research and Innovation, University Hospitals Bristol<br>Thomas, Ian; Cardiff University Centre for Trials Research<br>Kandiyali, Rebecca; Research and Innovation, University Hospitals Bristol<br>Mumford, Andrew; University Hospitals Bristol NHS Foundation Trust, Department of Haematology<br>Dick, Andrew; University of Bristol<br>Bagot, Catherine; Glasgow Royal Infirmary<br>Cooper, Nichola; Imperial College London<br>Hill, Quentin; Leeds Teaching Hospitals NHS Trust<br>Bradbury, Charlotte; University of Bristol, Cellular and Molecular Medicine; University Hospitals Bristol NHS Foundation Trust, Haematology

Keywords:	Immune Thrombocytopenia, ITP, Prednisolone, mycophenolate

SCHOLARONE™ Manuscripts

TITLE: A multicentre randomised trial of First Line treatment pathways for newly diagnosed Immune Thrombocytopenia: Standard steroid treatment versus combined steroid and mycophenolate.

Trial Acronym: The FLIGHT study

Authors: Julie Pell[1], Rosemary Greenwood[2], Jenny Ingram[2], Katherine Wale[3], Ian Thomas[1], Rebecca Kandiyali[3], Andrew Mumford[4,5], Andrew Dick[4,6], Catherine Bagot[7], Nichola Cooper[8], Quentin Hill[9], Charlotte Bradbury[4,5]

1 Centre for Trials Research, Cardiff University, PellJC@cardiff.ac.uk, thomasif@cardiff.ac.uk,

2 Research Design Service – South West, University of Bristol, rosemary.greenwood@UHBristol.nhs.uk, jenny.ingram@bristol.ac.uk,

3 Research & Innovation, University Hospitals Bristol NHS Foundation Trust, katharine.Wale@UHBristol.nhs.uk, rebecca.kandiyali@uhbristol.nhs.uk,

4 Cellular and Molecular Medicine, University of Bristol, a.mumford@bristol.ac.uk, a.dick@bristol.ac.uk, c.bradbury@bristol.ac.uk,

5 Department of Haematology, University Hospitals Bristol NHS Foundation Trust

6 UCL-Institute of Ophthalmology

7 Glasgow Royal Infirmary, Glasgow, Catherine.Bagot@ggc.scot.nhs.uk,

8 Imperial College London and Hammersmith Hospital, London, n.cooper@imperial.ac.uk,

9 Leeds Teaching Hospitals NHS Trust, quentinhill@nhs.net,

KEYWORDS

Immune Thrombocytopenia, Prednisolone, Mycophenolate, Corticosteroid

Corresponding author: Dr Charlotte Bradbury, Level 8, Bristol Haematology & Oncology Centre, Horfield Road, Bristol, BS2 8ED

Version: V3.0, 24-Oct 2017

Trial sponsor: University Hospitals Bristol NHS Foundation Trust, Research and Innovation, Level 3, UH Bristol Education and Research Centre, Upper Maudlin Street, Bristol BS2 8AE

Word Count: 3822

For peer review only - http://bmjopen.bmj.com/site/about/guidelines.xhtml

ABSTRACT

Introduction

Immune thrombocytopenia (ITP) is an autoimmune condition that may cause thrombocytopenia related bleeding. Current first line ITP treatment is with high dose corticosteroids but frequent side effects, heterogeneous responses and high relapse rates are significant problems with only 20% remaining in sustained remission with this approach. Mycophenolate (MMF) is often used as the next treatment with efficacy in 50-80% of patients and good tolerability but can take up to 2 months to work.

Objective: To test the hypothesis that MMF combined with corticosteroid is a more effective first line treatment for immune thrombocytopenia (ITP) than current standard of corticosteroid alone.

Methods and analysis

Design: Multicentre, UK based, open label, randomised controlled trial

Setting: Haematology departments in secondary care

Participants: We plan to recruit 120 patients >16 years old with a diagnosis of ITP and a platelet count <30 x10^9/L who require first line treatment. Patients will be followed up for a minimum of 12 months following randomisation.

Primary outcome: Time from randomisation to treatment failure defined as platelets <30x10^9/L and a need for 2nd line treatment.

Secondary outcomes: Remission rates, time to relapse and next therapy, cumulative corticosteroid dose, side effects, bleeding events, rescue therapy, splenectomy, socioeconomic costs, patient reported outcomes (quality of life, fatigue, impact of bleeding, care costs).

Analysis: The sample size of 120 achieves a 91.5% power to detect a doubling of the median time to treatment failure. This will be expressed as a hazard ratio with 95% confidence interval, median time to event if more than 50% have had an event and illustrated with Kaplan Meier curves. Cost effectiveness will be based on the first 12 months from diagnosis.

Ethics and dissemination

Ethical approval from NRES Committee South West (IRAS number 225959). EudraCT Number: 2017-001171-23. Results will be submitted for publication in peer-reviewed journals.

For peer review only

For peer review only - http://bmjopen.bmj.com/site/about/guidelines.xhtml

STRENGTHS AND LIMITATIONS OF THIS STUDY

- First UK multicentre RCT for first line treatment of ITP
- Independent funding from NIHR testing a pragmatic, cost effective approach which if effective, may be applicable to other autoimmune conditions
- The trial includes patient oriented outcomes by using validated questionnaires to assess quality of life, fatigue, impact of bleeding and care costs
- Option to consent to additional blood samples for translational research to maximise scientific potential
- The limitations include the lack of very long term follow up and sample size to detect only moderate differences between treatment arms

FUNDING

The FLIGHT trial is independent research funded by the National Institute for Health Research (NIHR) under its Research for Patient Benefit (RfPB) Programme (Grant Reference Number PB-PG-0815-20016). The views expressed are those of the authors and not necessarily those of the NHS, the NIHR or the Department of Health.

CONFLICTS OF INTEREST

None of the authors have competing interests

INTRODUCTION

Immune thrombocytopenia (ITP) has an incidence of 2.9/100,000 person-years. [1] It is an autoimmune condition that presents with bleeding and bruising due to a low platelet count. In ITP, there is increased consumption and reduced production of platelets due to both antibody and cell mediated autoimmune attack of platelets and megakaryocytes involving dysregulated autoreactive T and B cell lymphocytes. [2-5]

Current first line ITP treatment is with high dose corticosteroids but this has several downsides. First, the majority of patients suffer significant side effects including mood swings, difficulty sleeping, weight gain, high blood pressure, diabetes, gastric irritation, skin thinning and osteoporosis. A published survey of ITP patients reported 98% had at least one side effect and 38% stopped or reduced dosage due to intolerable side effects.[6] In the UK ITP registry, the most frequently reported co-morbidities were related to corticosteroids and correlated with duration of treatment (hypertension in 30%, diabetes in 19%) (Newland A et al, poster BSH 2015). The second problem is

For peer review only - http://bmjopen.bmj.com/site/about/guidelines.xhtml

that patients are heterogeneous in their response to corticosteroid with some (approximately 20%) not responding at all and the majority of others (70%-90%) relapsing when the corticosteroids are reduced or stopped. [7-9] Patients who are refractory or relapse (the majority), remain at risk of bleeding/bruising, which occasionally can be severe including intracranial haemorrhage.[10] They often receive more corticosteroid with associated side effects. Some require hospital admission and expensive rescue therapies (e.g. IVIG for a 70kg patient=£3,906). They continue to require frequent blood tests and doctor visits and are usually unable to continue their normal activities until their illness is controlled. Fatigue is also associated with disease activity and can be severe. [11] Physical factors combine with psychological stress through fear of bleeding, need for time off work and lifestyle restrictions due to bleeding risk to adversely impact quality of life.[12 13]

First line treatment for ITP is unsatisfactory but it has been unchallenged for decades. The lack of new approaches has arisen from a chronic lack of research funding and clinical trials. The few trials done have been funded by pharmaceutical companies, risking publication bias towards high cost non-generic drugs. The relative rarity (2.9/100,000 person-years), non-cancerous nature and rare impact on survival of ITP have prevented ITP being a priority for research funding in the past. However, this underestimates the profound adverse impact a diagnosis of ITP and its treatment can have for individual patients, many of whom are young. There is also a costly financial impact for the NHS from the healthcare resources patients require when their illness is uncontrolled. In addition, the problems faced by patients with ITP mirror those with other autoimmune conditions which as a group are common, affecting 3% of the population. There is an urgent clinical need to address this inequality, improving first line treatment for ITP through high quality, independently funded research to allow patients with this condition access to improvements in care seen in other illnesses such as cancer or heart disease.

Current popular options for second line or subsequent treatment include Mycophenolate, Rituximab, Thrombopoeitin receptor agonists (TPO RA) and splenectomy. Splenectomy is an effective treatment (60% long term remission rates) but irreversible and international guidelines recommend deferring splenectomy for the first 12 months following diagnosis due to the chance of spontaneous remission (risk of unnecessarily removing a healthy organ).[7, 9] Surgical operations are not popular with patients and there is increasing awareness of the short and long term complications of splenectomy including infection, bleeding, arterial and venous thrombosis, cancer and relapse.[14] The splenectomy numbers performed in the UK has dramatically reduced over recent years (UK ITP registry data). Rituximab is a monoclonal antibody treatment which targets antibody

For peer review only - http://bmjopen.bmj.com/site/about/guidelines.xhtml

production by B cells. It is relatively expensive, with disappointing long term remission rates similar to placebo.[14] TPO RA stimulate platelet production, are well tolerated and effective in the majority[15] but at significant financial cost, prohibiting widespread use in the UK for early treatment (NICE guidance). A small (n=12) non-randomised study using TPO RA with corticosteroid first line showed efficacy but perhaps less than expected.[16] By contrast, Mycophenolate (MMF) is a widely used second line agent in the UK due to good efficacy (response rates of 50-80%), safety and tolerability profile.[17-24] Mycophenolate has activity against both autoreactive T and B cells and has also shown efficacy in refractory ITP including steroid resistance suggesting a complimentary mechanism of action. [22] It is less expensive to the NHS than some other second line options costing approximately £182/year (generic cost) compared to costs for average doses of romiplostim (TPO RA) at £25 000/year, Eltrombopag (TPO RA) £20 000/year or rituximab at £8000 for a course of 4 infusions. However, similar to other second line therapies, MMF has a relatively slow (up to 2 months) onset of action. In the meantime, patients often receive further steroid (to maintain a "safe platelet count") and continue to suffer problems associated with their illness (see above). Direct feedback from patients regarding the difficulties they face in the first months following ITP diagnosis has been the primary driving force for this clinical trial. Local (Bristol) and national patient groups (ITP support association) have been fundamental to the formulation of patient relevant priorities for treatment.

Rationale

The Flight trial is the first UK, NHS coordinated, Pharma independent multicentre randomised controlled trial, testing a “common sense/practical” new approach using MMF first line instead of second line with the aim of preventing the almost inevitable first relapse when corticosteroids stop. Patients will be randomly allocated to one of 2 treatment arms, either standard of care (corticosteroid alone) or MMF combined with corticosteroid with the primary outcome of time to treatment failure. By giving patients a stable platelet count sooner, we expect to improve other outcomes such as quality of life and fatigue. By reducing the risk of relapse, patients may also be less likely to receive a second course of corticosteroid with associated side effects. Potential indirect benefits to the NHS include reduced need for rescue treatments, blood tests, hospital attendances and admissions and reduced need for high cost treatments such as TPO mimetics. However, there will be some patients who will be treated with MMF who may have been successfully treated with corticosteroids alone (10-30%).[7-9] Similar to other immunosuppressives, MMF may slightly increase infection and cancer risk with long term use (SmPC) In addition, MMF is teratogenic and therefore stringent pregnancy prevention is essential for men and women taking the drug. This puts the trial in

For peer review only - http://bmjopen.bmj.com/site/about/guidelines.xhtml

equipoise. The trial includes a strategy to reduce and stop MMF at 6 months for patients in complete remission to prevent unnecessary long-term use.

The choice of this open label design was made in order to allow true patient treatment costs to be calculated for the cost effectiveness analysis, and to deal with the complexities of placebo controlling a drug that needed titrating at the start and tapering at the end. In addition, over encapsulation was only possible for the lower MMF dose (250 mg) and the resulting capsule was the largest size which would mean most patients taking 8 large capsules per day in both arms; something that patients in Bristol thought would put them off taking part in the study. Patients were clear that from their perspective that a straight forward open label design would be preferable and was easier for a new patient to understand and consent. In addition, the quotes from 2 separate companies also showed the financial costs of encapsulation to generate a placebo were prohibitively expensive.

This trial proposal has received support and input from clinicians and patients nationally (UK ITP forum and ITP support association). To ensure objective and meaningful outcomes, it will be a multicentre RCT, aiming to recruit 120 patients (expecting 100 full datasets). Patients will be given up to one week of corticosteroid prior to randomisation to enable sufficient time to read information, discuss and ask questions with informed consent in an appropriate setting. Patients will receive the usual follow up according to clinical need and local policy. Laboratory and clinical data will be collected from routine appointments. In addition, patient oriented outcomes will be recorded at diagnosis, 2, 4, 6, and 12 months using validated patient questionnaires. Patients are also offered consent to additional blood samples for translational research studies (time 0 and 2 months).

OBJECTIVES

Primary objective

To compare two first line treatment pathways for ITP, standard corticosteroid only versus corticosteroid combined with MMF and demonstrate which pathway helps patients achieve a stable platelet count sooner, measured as survival free from treatment failure (time from randomisation to treatment failure).

For peer review only - http://bmjopen.bmj.com/site/about/guidelines.xhtml

Secondary objectives are to collect:

- data on time to treatment failure as measured objectively by a fall in platelet count to <30x10^9/L and need for new treatment.
- clinical data on additional treatments (including "rescue treatments" such as IVIG).
- clinical data on cumulative steroid dose.
- clinical data on additional investigations, hospital appointments and admissions.
- patient reported outcomes through standardised questionnaires.
- resource use data relating to an NHS/PSS and societal perspective.
- data on treatment side effects and adverse events (including bleeding, infections and steroid side effects)

For peer review only

METHODS AND ANALYSIS

Trial design

A multicentre, open label randomised clinical trial of MMF with corticosteroid as first line treatment for patients with ITP versus the standard care pathway of corticosteroids alone as first line treatment.

Eligibility criteria

Inclusion criteria: Patients (males and females) >16 years old with a diagnosis of ITP, a platelet count <30x10^9/L AND a clinical need for first line treatment. Patients have provided written informed consent.

Exclusion criteria: Pregnancy and breastfeeding (Women of child bearing potential require a pregnancy test result within 7 days prior to randomisation to rule out unintended pregnancy). Patients with HIV, Hepatitis B or C, or Common Variable immunodeficiency. Contraindications to MMF or corticosteroid (see SmPC) including patients with active significant infections, hypersensitivity to mycophenolate mofetil, mycophenolic acid or to any of the excipients or active significant infection. Patients not capable of giving informed consent (e.g. due to incapacity). Patients (men and women) unwilling to follow contraceptive advice if allocated to MMF treatment arm

For peer review only - http://bmjopen.bmj.com/site/about/guidelines.xhtml

Study setting

120 patients will be recruited from approximately 40 Haematology departments of hospitals (secondary care) across the UK where ITP patients are treated.

The trial processes will be run by the Centre for Trials Research (CTR), Cardiff University and sponsored by University Hospitals Bristol NHS Foundation Trust.

Randomisation

Patients who agree to participate will be randomised to MMF with corticosteroid or corticosteroid alone in a 1:1 ratio using a web based randomisation system based at Cardiff CTR. Randomisation will be stratified by primary or secondary ITP diagnosis. Due to the large number of centres and the small number of patients it will not be sensible to stratify randomisation by study centre. However, to ensure an even spread of patients across time, randomisation will be blocked using random block sizes of 6 and 8 to retain concealment.

Treatment arms (Figures 1 and 2):

1. **Corticosteroid +MMF pathway:** 1mg/kg od prednisolone 4 days (maximum of 100mg), 40mg od 2 weeks, 20mg od 2 weeks, 10mg od 2 weeks, 5mg od 2 weeks then 5mg alternate days 2 weeks then stop*. For the duration of steroid, patients will get a PPI or H2 antagonist to protect against gastric bleeding and appropriate bone protection.

*Dexamethasone 20mg or 40mg daily for 4 days is an alternative option to prednisolone if deemed clinically more appropriate for individual circumstances. Any steroid commenced prior to randomisation will be deducted from the regime above.

From randomisation (alongside steroid), MMF 500mg bd starting dose then increased to 750mg bd after 2 weeks if tolerated and 1g bd after another 2 weeks if tolerated (4 weeks after starting). Earlier dose escalation to MMF 1g bd can be considered if clinically indicated.

After 6 months of MMF therapy, all patients who have remained in complete remission (pl count> 100 x10 ^{9}/L) will reduce the dose by 250mg (one capsule) each month. The aim is to continue on the lowest dose that achieves a haemostatic (safe) platelet count (pl >30 x10 ^{9}/L) and to ensure that patients who have gone into a spontaneous remission do not continue to take the drug indefinitely.

For peer review only - http://bmjopen.bmj.com/site/about/guidelines.xhtml

2. **Corticosteroid only pathway:** 1mg/kg od prednisolone 4 days (maximum of 100mg), 40mg od 2 weeks, 20mg od 2 weeks, 10mg od 2 weeks, 5mg od 2 weeks then 5mg alternate days 2 weeks then stop*. For the duration of steroid, patients will get a PPI or H2 antagonist to protect against gastric bleeding and appropriate bone protection.

*Dexamethasone 20mg or 40mg orally daily for 4 days is an alternative option to prednisolone if deemed clinically more appropriate for individual circumstances. Any steroid commenced prior to randomisation will be deducted from the regime above.

In both groups: Any steroid commenced prior to randomisation will be deducted from the regimes. Importantly, emergency and rescue treatments will be permitted throughout the study. These include platelet transfusions, tranexamic acid and intravenous immunoglobulin. These are known not to impact on the natural history of ITP and it is recognised that they may be important for patient safety. The use of “rescue treatments” will be recorded on the CRF.

In addition, some degree of flexibility of corticosteroid dose and duration may be needed for individual patients according to comorbidity, tolerability and other factors.

If treatment failure occurs, choice of second line treatment will be individualised according to patient’s clinical circumstances. Further steroid will be given according to clinical need.

Primary outcome is time from randomisation to treatment failure defined as a pl count <$30x10^9$/L AND a need to commence second line treatment. This will include patients who are refractory (pl <$30x10^9$/L in spite of 2 weeks treatment in the steroid arm or pl <$30x10^9$/L in spite of 2 months treatment in the steroid +MMF arm) or who initially respond but then relapse (defined clinically as pl <$30x10^9$/L and need for further therapy).

Secondary outcomes

1. Remission rates (pl >30 $x10^9$/L and at least 2 fold increase from baseline); Complete >$100x10^9$/L, partial 30-$100x10^9$/L.
2. Time to relapse and time to next therapy
3. Cumulative corticosteroid dose
4. Bleeding events
5. Need for rescue therapies
6. Need for splenectomy

For peer review only - http://bmjopen.bmj.com/site/about/guidelines.xhtml

7. Time off work

8. Number of hospital admissions, day unit and clinic attendances

9. Fatigue and Quality of life

10. Medication side effects, toxicity or other adverse events (including infection episodes)

11. NHS costs and Personal and social costs.

Data collection (table 1)

Hospital monitoring of platelet levels (FBC) is part of routine care for ITP patients and these data will be collected and recorded on the CRF without requiring patients to come in for additional samples to be taken. These locally collected samples may be collected monthly (or less often) for patients believed to be in stable remission and weekly at lower or declining platelet levels. We expect this to allow us to calculate the time in remission and time to relapse with reasonable accuracy over the 12 to 24 month follow up period. Other clinical and laboratory data needed for the trial endpoints will be collected from the medical and electronic records and recorded on the CRF. In addition, we will also ask the patients to complete questionnaires on fatigue, quality of life and bleeding scores at baseline, 2, 4, 6, and 12 months.

Patient reported outcomes will be captured by the following questionnaires:

1. SF36v2 (Your health & wellbeing) – Quality of life,
2. FACIT-Fatigue (version 4) – Fatigue,
3. FACT-Th6 (version 4)- Bleeding,
4. ICECAP-A V2- Quality of life,
5. Health economic/resource use questionnaire - Personal and social costs.

Additional optional research blood samples (requiring separate consent) will be sent at baseline and 2 month follow up to the Bristol Biobank.

Data Management

Source documents produced for this trial will be kept in the patient's hospital records and source data will be transcribed into trial-specific Case Report Forms (CRFs) at the end of each patient visit. Data recording for this trial will be via a web based system. This is a secure encrypted system accessed by an institutional password which complies with Data Protection Act standards. The database will be stored and regularly backed up on a Cardiff University Server. The CRFs will be coded with the study number and will not include patients' names and addresses

For peer review only - http://bmjopen.bmj.com/site/about/guidelines.xhtml

STATISTICS AND DATA ANALYSIS

Sample size calculation

There is no published clinical data available for MMF use first line in ITP as this is a novel approach. We have analysed local data on MMF used second line in ITP in 12 patients which shows an estimated median survival free from treatment failure of more than 10 months. We have data on 68 who experienced corticosteroids as a first line treatment showing that 70% of them had experienced a treatment failure by 12 months and that the median survival free from treatment failure was 5.0 months (95% CI [3.2, 6.8]). Data for the 12 patients treated with MMF second line therapy have shorter follow-up times, with only 5 patients having follow up beyond 12 months. The cox proportional hazards ratio model demonstrates the 90% confidence interval for the hazard ratio to be between 0.13 and 0.59, showing that our decision to power this on an estimate of a hazard of lower than 0.5 is potentially achievable.

Clinically a doubling in the time to remission was thought to be something that the patients would have welcomed. Less than that was not thought to be sufficient grounds for switching this treatment from second line to first line due to the potential for additional toxicity and immune suppression in those who may have remained in remission with corticosteroids alone.

The sample size of 120 (60 per group) with less than 5% loss to follow-up achieves 91.5% power to detect a doubling of the median time to treatment failure from 5 months to 10 months if the patients are recruited at a steady rate or 10 per month for 12 months and all followed up until the last patients reaches 12 months follow up.

Statistical analysis

The full statistical analysis will be written into a statistical analysis plan available separately. The analysis will produce a CONSORT diagram for the reporting of clinical trials.

The baseline characteristics of the two groups will be tabulated but not tested for statistically significant differences between the groups.

The primary analysis is by intention to treat. However an investigation of compliance with the treatment pathway and compliance with the criteria for changing to a second line therapy will be carried out prior to the primary analysis to check the date of the primary event. The primary event is the date at which there was a requirement for second line therapy. Where the platelet count falls

For peer review only - http://bmjopen.bmj.com/site/about/guidelines.xhtml

below the level required for this treatment decision, the first date at which either symptoms or a blood test revealing this event will be used. If a clinician decides to use a second line therapy without a platelet count below the criteria, the date of the treatment decision/new prescription will be taken to represent that event. The results will be expressed as a hazard ratio with 95% confidence interval, median time to event if more than 50% have had an event and plotted as Kaplan Meier curves.

The primary analysis will contain all patients who are randomised for as long as they have been followed up or until their first event in a survival analysis using intention to treat methodology. All patients will be followed up to 12 months. In addition, patients who have not had an event in the first 12 months post randomisation will be followed until their first event or until the last patient has reached the 12 month point – whichever is the sooner and included in the analysis until that time accordingly.

Analysis of other outcomes will use as full a data set as possible and focus on the 12 month data point or area under the curve as appropriate and detailed in the analysis plan.

No interim analyses of the main endpoint will be supplied to the independent Data Monitoring Committee (DMC) due to the short time frame (12 months recruitment) in which all patients will be recruited by the time the first patient has completed follow-up. Serious adverse event rates will be reported on a monthly basis to the trial management group (TMG) and the DMC. The DMC could advise the chairman of the Trial Steering Committee and Chief Investigator if these provide proof beyond reasonable doubt that it would be unethical to continue with the trial.

Patient and Public Involvement and Engagement

During the trial development, a group of 8 ITP patients discussed the study design, burden of outcome measure completion to patients and the size of a potential placebo capsule which they reported could put them off getting involved in a trial. They reported that avoidance of relapse, early achievement of a stable platelet count, reduced overall corticosteroid dose and reduced hospital attendances are the most important goals for ITP management from their perspective.

We formed a Patient Advisory Group (PAG) with some of these patients and representatives from the ITP association that will advise the trial management group throughout the study. They have commented on all patient-facing documentation and will be instrumental in disseminating the study findings to patient groups and the public.

For peer review only - http://bmjopen.bmj.com/site/about/guidelines.xhtml

Author Contributions: Charlotte Bradbury was responsible for writing the protocol with clinical input from Nichola Cooper, Quentin Hill and Catherine Bagot. Rosemary Greenwood is the trial statistician who has contributed to the trial design and writing of protocol. Jenny Ingram contributed to trial design and leads the Patient Advisory Group input. Rebecca Kandiyali is the trial health economist and provided input to the trial design and protocol. Julie Pell and Ian Thomas (Cardiff CTR) and Katharine Wale (sponsor representative) have also provided contribution to writing the protocol. Andrew Mumford and Andrew Dick have provided mentorship to the Chief investigator.

Acknowledgements: We would like to thank the members of the UK ITP forum who have been invaluable providing feedback on the trial design and recruitment to the trial.

Figure 1. Flight treatment pathway: Corticosteroid only

Figure 2. Flight treatment pathway: Corticosteroid and Mycophenolate

For peer review only

Table 1: Time schedule of enrolment, interventions, assessments and visits

**Procedures**	**V0**	**V1**	**V2 (2 mths)**	**V3 (4 mths)**	**V4 (6 mths)**	**V5 (12 mths)**	**V6 12-24 mths**
	**Screen**	**Baseline / Randomisation to pathway 1 or 2**	**Follow up**	**Follow up**	**Follow up**	**Follow up**	**Data collection from sites**
Eligibility assessment	x						
Randomisation		x					
Informed consent		x					
Demographics		x					
Medical history		x	x	x	x	x	
Physical examination		x					
Vital signs (incl height & weight)		x	x	x	x	x	
Pregnancy test	x						
Concomitant medications		x	x	x	x	x	
Standard practice bloods (includes blood sugar if applicable)	x	x	x	x	x	x	
Hepatitis B, C & HIV serology	x						
Immunoglobulins (blood)		x			x	x	
Extra blood samples (optional)		x	x				
Dispensing of trial drugs		x*					
Compliance			x				
QofL FACT-Th6, V4	x	x	x	x	x	x	
QofL ICECAP V2 – A measure	x	x	x	x	x	x	
QofL SF-36v2 – Health Survey	x	x	x	x	x	x	
QofL FACIT-F, V4, pg 3 (fatigue)	x	x	x	x	x	x	
QofL Thrombocytopenia costs questionnaire	x	x	x	x	x	x	
Data collection from sites on platelet count & treatment		x	x	x	x	x	x

* MMF and corticosteroid dispensing frequency can follow standard local practice.

For peer review only - http://bmjopen.bmj.com/site/about/guidelines.xhtml

REFERENCES

1. Moulis G, Palmaro A, Montastruc JL, et al. Epidemiology of incident immune thrombocytopenia: a nationwide population-based study in France. *Blood* 2014;124(22):3308-15. doi: 10.1182/blood-2014-05-578336 [published Online First: 2014/10/12]
2. McKenzie CG, Guo L, Freedman J, et al. Cellular immune dysfunction in immune thrombocytopenia (ITP). *British journal of haematology* 2013;163(1):10-23. doi: 10.1111/bjh.12480 [published Online First: 2013/08/14]
3. Iraqi M, Perdomo J, Yan F, et al. Immune thrombocytopenia: antiplatelet autoantibodies inhibit proplatelet formation by megakaryocytes and impair platelet production in vitro. *Haematologica* 2015;100(5):623-32. doi: 10.3324/haematol.2014.115634 [published Online First: 2015/02/16]
4. Ji X, Zhang L, Peng J, et al. T cell immune abnormalities in immune thrombocytopenia. *Journal of hematology & oncology* 2014;7:72. doi: 10.1186/s13045-014-0072-6 [published Online First: 2014/10/03]
5. Audia S, Rossato M, Santegoets K, et al. Splenic TFH expansion participates in B-cell differentiation and antiplatelet-antibody production during immune thrombocytopenia. *Blood* 2014;124(18):2858-66. doi: 10.1182/blood-2014-03-563445 [published Online First: 2014/09/19]
6. Brown TM, Horblyuk RV, Grotzinger KM, et al. Patient-reported treatment burden of chronic immune thrombocytopenia therapies. *BMC blood disorders* 2012;12:2. doi: 10.1186/1471-2326-12-2 [published Online First: 2012/03/23]
7. Provan D, Stasi R, Newland AC, et al. International consensus report on the investigation and management of primary immune thrombocytopenia. *Blood* 2010;115(2):168-86. doi: 10.1182/blood-2009-06-225565 [published Online First: 2009/10/23]
8. Neunert CE. Management of newly diagnosed immune thrombocytopenia: can we change outcomes? *Hematology American Society of Hematology Education Program* 2017;2017(1):400-05. doi: 10.1182/asheducation-2017.1.400 [published Online First: 2017/12/10]
9. Neunert C, Lim W, Crowther M, et al. The American Society of Hematology 2011 evidence-based practice guideline for immune thrombocytopenia. *Blood* 2011;117(16):4190-207. doi: 10.1182/blood-2010-08-302984 [published Online First: 2011/02/18]
10. Neunert C, Noroozi N, Norman G, et al. Severe bleeding events in adults and children with primary immune thrombocytopenia: a systematic review. *Journal of thrombosis and haemostasis : JTH* 2015;13(3):457-64. doi: 10.1111/jth.12813 [published Online First: 2014/12/17]
11. Hill QA, Newland AC. Fatigue in immune thrombocytopenia. *British journal of haematology* 2015;170(2):141-9. doi: 10.1111/bjh.13385 [published Online First: 2015/04/01]
12. Snyder CF, Mathias SD, Cella D, et al. Health-related quality of life of immune thrombocytopenic purpura patients: results from a web-based survey. *Current medical research and opinion* 2008;24(10):2767-76. doi: 10.1185/03007990802377461 [published Online First: 2008/08/22]
13. Tarantino MD, Mathias SD, Snyder CF, et al. Impact of ITP on physician visits and workplace productivity. *Current medical research and opinion* 2010;26(2):319-28. doi: 10.1185/03007990903451298 [published Online First: 2009/12/09]
14. Kristinsson SY, Gridley G, Hoover RN, et al. Long-term risks after splenectomy among 8,149 cancer-free American veterans: a cohort study with up to 27 years follow-up. *Haematologica* 2014;99(2):392-8. doi: 10.3324/haematol.2013.092460 [published Online First: 2013/09/24]

For peer review only

For peer review only - http://bmjopen.bmj.com/site/about/guidelines.xhtml

15. Rodeghiero F, Ruggeri M. Treatment of immune thrombocytopenia in adults: the role of thrombopoietin-receptor agonists. *Seminars in hematology* 2015;52(1):16-24. doi: 10.1053/j.seminhematol.2014.10.006 [published Online First: 2015/01/13]
16. Gomez-Almaguer D, Herrera-Rojas MA, Jaime-Perez JC, et al. Eltrombopag and high-dose dexamethasone as frontline treatment of newly diagnosed immune thrombocytopenia in adults. *Blood* 2014;123(25):3906-8. doi: 10.1182/blood-2014-01-549360 [published Online First: 2014/05/08]
17. Miano M, Ramenghi U, Russo G, et al. Mycophenolate mofetil for the treatment of children with immune thrombocytopenia and Evans syndrome. A retrospective data review from the Italian association of paediatric haematology/oncology. *British journal of haematology* 2016;175(3):490-95. doi: 10.1111/bjh.14261 [published Online First: 2016/10/28]
18. Taylor A, Neave L, Solanki S, et al. Mycophenolate mofetil therapy for severe immune thrombocytopenia. *British journal of haematology* 2015;171(4):625-30. doi: 10.1111/bjh.13622 [published Online First: 2015/08/08]
19. Kotb R, Pinganaud C, Trichet C, et al. Efficacy of mycophenolate mofetil in adult refractory auto-immune cytopenias: a single center preliminary study. *European journal of haematology* 2005;75(1):60-4. doi: 10.1111/j.1600-0609.2005.00437.x [published Online First: 2005/06/11]
20. Provan D, Moss AJ, Newland AC, et al. Efficacy of mycophenolate mofetil as single-agent therapy for refractory immune thrombocytopenic purpura. *American journal of hematology* 2006;81(1):19-25. doi: 10.1002/ajh.20515 [published Online First: 2005/12/22]
21. Hou M, Peng J, Shi Y, et al. Mycophenolate mofetil (MMF) for the treatment of steroid-resistant idiopathic thrombocytopenic purpura. *European journal of haematology* 2003;70(6):353-7. [published Online First: 2003/05/21]
22. Colovic M, Suvajdzic N, Colovic N, et al. Mycophenolate mophetil therapy for chronic immune thrombocytopenic purpura resistant to steroids, immunosuppressants, and/or splenectomy in adults. *Platelets* 2011;22(2):153-6. doi: 10.3109/09537104.2010.520372 [published Online First: 2010/12/15]
23. Zhang WG, Ji L, Cao XM, et al. Mycophenolate mofetil as a treatment for refractory idiopathic thrombocytopenic purpura. *Acta pharmacologica Sinica* 2005;26(5):598-602. doi: 10.1111/j.1745-7254.2005.00088.x [published Online First: 2005/04/22]
24. Howard J, Hoffbrand AV, Prentice HG, et al. Mycophenolate mofetil for the treatment of refractory auto-immune haemolytic anaemia and auto-immune thrombocytopenia purpura. *British journal of haematology* 2002;117(3):712-5. [published Online First: 2002/05/25]

For peer review only

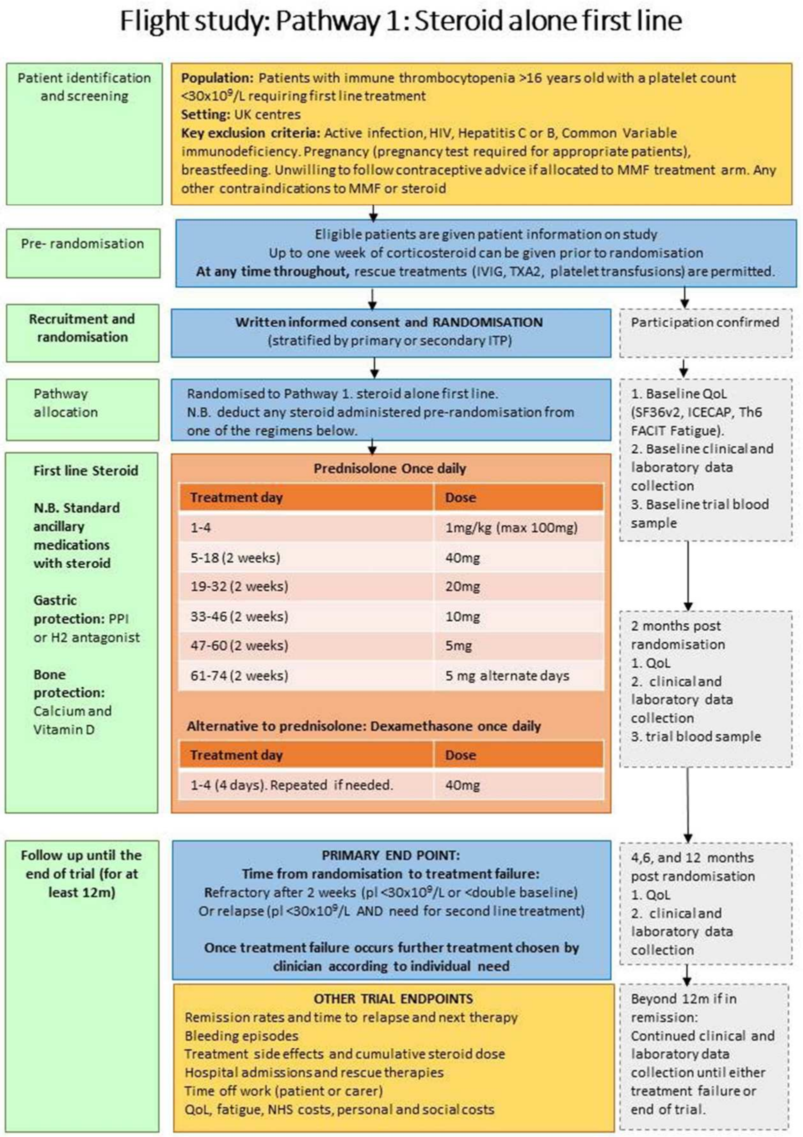

Figure 1. Flight treatment pathway: Corticosteroid only

190x275mm (96 x 96 DPI)

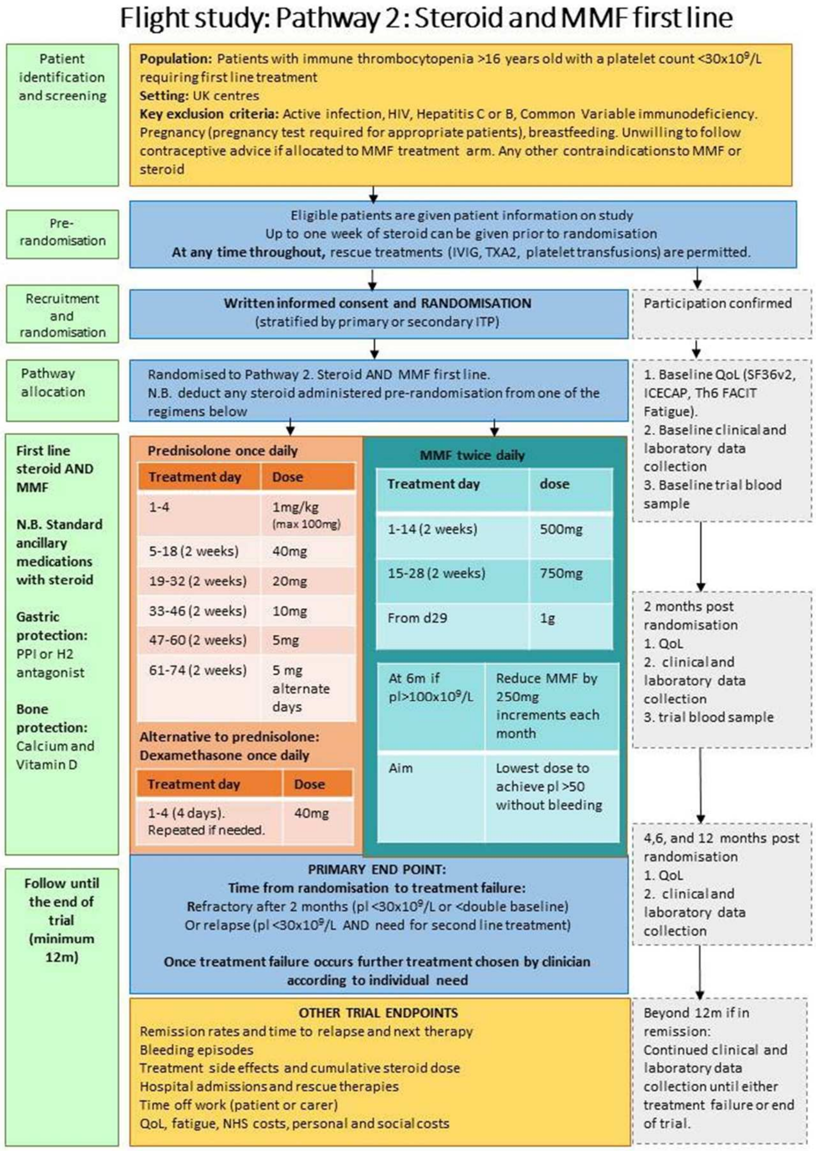

Figure 2. Flight treatment pathway: Corticosteroid and Mycophenolate

190x275mm (96 x 96 DPI)

A multicentre randomised trial of First Line treatment pathways for newly diagnosed Immune Thrombocytopenia: Standard steroid treatment versus combined steroid and mycophenolate.

The **FLIGHT** study

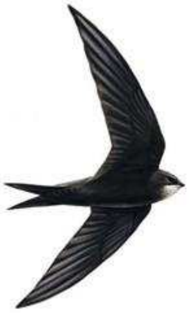

IRAS Number: 225959

EudraCT Number: 2017-001171-23

SPONSORS Number: ON/2016/6004

FUNDERS Number: PB-PG-0815-20016

For peer review only - http://bmjopen.bmj.com/site/about/guidelines.xhtml

SIGNATURE PAGE

The undersigned confirm that the following protocol has been agreed and accepted and that the Chief Investigator agrees to conduct the trial in compliance with the approved protocol and will adhere to the principles outlined in the Medicines for Human Use (Clinical Trials) Regulations 2004 (SI 2004/1031), amended regulations (SI 2006/1928) and any subsequent amendments of the clinical trial regulations, GCP guidelines, the Sponsor's SOPs, and other regulatory requirements as amended.

I agree to ensure that the confidential information contained in this document will not be used for any other purpose other than the evaluation or conduct of the clinical investigation without the prior written consent of the Sponsor

I also confirm that I will make the findings of the study publically available through publication or other dissemination tools without any unnecessary delay and that an honest accurate and transparent account of the study will be given; and that any discrepancies from the study as planned in this protocol will be explained.

Chief Investigator:

Signature: Date:

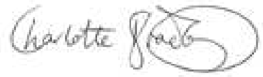

....

24/October/2017

Name: (please print):

Charlotte Bradbury

....

For peer review only

For peer review only - http://bmjopen.bmj.com/site/about/guidelines.xhtml

AMENDMENT HISTORY

Amendment No.	Protocol version no.	Date issued	Author(s) of changes	Details of changes made
	V1.0	11 May 2017		New Protocol
	V2.0	20 July 2017	Julie Pell	1) Add the option for sites to dispense MMF and steroid frequency as per standard local practice.<br>2) Add 2 new investigator sites in Scotland<br>3) Change PIs at 6 existing investigator sites<br>4) Amend RSI section and web link to IMP SPC in line with SPC submitted in initial applications to REC & MHRA
	V3.0	24 Oct 2017	Charlotte Bradbury / Julie Pell	1) Pg 6, Trial Summary:<br>a) clarify the term "steroid" refers to corticosteroid,<br>b) clarify 'newly diagnosed' with regard to first line treatment;<br><br>2) Treatment Pathway 1 & 2. Section 1: Written Informed consent and RANDOMISATION (stratified by primary or secondary ITP, ~~age~~). Age has been removed as there is no evidence of age affecting relapse (there is no other reference to stratification factors in the protocol);<br><br>3) 7.2 Exclusion criteria: Clarify that sites need to ensure patients adequately understand and comply with pregnancy and contraception advice;<br><br>4) 8.4.1 Additional consent: Clarify sample and data collection if a patient withdraws;<br><br>5) 8.6 Trial Interventions: Clarification of steroid dose and duration;

For peer review only

Cardiff University Centre for Trials Research / Prifysgol Caerdydd Canolfan Ymchwil Treialon — NHS National Institute for Health Research — itp — University Hospitals Bristol NHS Foundation Trust

				6) 8.9 & Appendix 1 (Schedule of Procedures): Specific study follow up visits: Blood sugars have been removed as these are usually only performed on patients taking steroids;<br><br>7) 8.13 Laboratory study: Update of sample logistics to and from sites;<br><br>8) 9.8 Assessment of compliance: Removal of last bullet point as ALL patients will be included in final analysis;<br><br>9) Section 10 Pharmacovigilance: Reviewed and amended by CTR Safety Team to bring this section in line with CTR SOPs;<br><br>10) 14.3 Protocol compliance: Recommended changes from the Trial Steering Committee (It was felt the dose regimen was clearly reflective of the generality of clinical practice in this area, and that small deviations were likely to happen during the study but were not likely to be clinically significant. All dosages will be recorded in the case report forms. It was recommended that the wording in the protocol be amended to allow minor variations to not count as formal protocol violations.<br><br>11) Grammatical corrections and updated contact details.

For peer review only

For peer review only - http://bmjopen.bmj.com/site/about/guidelines.xhtml

KEY TRIAL CONTACTS

Chief Investigator	Dr Charlotte Bradbury<br>Bristol Haemophilia centre<br>Location D701, Level 7<br>Bristol Haematology & Oncology Centre<br>Horfield Road<br>Bristol, BS2 8ED<br>Consultant Senior Lecturer Haematology<br>Email: c.bradbury@bristol.ac.uk<br>Mobile: 07973353316
Trial Co-ordinator	Julie Pell, Trial Manager / Paula Foscarini-Craggs, Data Manager , Centre for Trials Research, College of Biomedical & Life Sciences, Cardiff University, Neuadd Meirionydd, Heath Park, Cardiff CF14 4YS<br>Email:<br>FLIGHT@cardiff.ac.uk
Sponsor	University Hospitals Bristol NHS Foundation Trust, Research and Innovation, Level 3, UH Bristol Education and Research Centre, Upper Maudlin Street, Bristol BS2 8AE
Funder(s)	NIHR Funded Research for Patient Benefit grant<br>ITP (sub study)
Clinical Trials Unit	Centre for Trials Research [CTR]), Cardiff University<br>College of Biomedical & life Sciences<br>University Hospital of Wales<br>Heath Park<br>Cardiff, CF14 4XN
Statistician	Rosemary Greenwood<br>Senior Statistician<br>Research Design Service – South West

For peer review only - http://bmjopen.bmj.com/site/about/guidelines.xhtml

	Honorary Senior Lecturer, School of Social and Community Medicine, University of Bristol.<br>Based at University Hospitals Bristol NHS Foundation Trust, Level 3 Education Centre<br>Upper Maudlin Street, Bristol BS2 8AE,<br>Tel: 0117 34 20234,<br>E-mail: Rosemary.Greenwood@UHBristol.nhs.uk
Emergency contact details	Julie Pell<br>Trials Manager<br>Centre for Trials Research<br>02922 510528<br>Email: pelljc@cardiff.ac.uk

For peer review only - http://bmjopen.bmj.com/site/about/guidelines.xhtml

TRIAL SUMMARY

Trial Title	A multicentre randomised trial of First Line treatment pathways for newly diagnosed Immune Thrombocytopenia: Standard steroid treatment versus combined steroid and mycophenolate.<br><br>1) Throughout this protocol the term "steroid" refers to corticosteroid<br>2) Patients do not necessarily need to have a brand new ITP diagnosis, but should be eligible for first line treatment. For example, patients may have been observed initially before treatment is needed.
Internal ref. no. (or short title)	The Flight Study
Funding	NIHR & ITP (sub study)
Trial Design	Randomised, open label, multicentre
Trial Participants	Patients (males and females) >16 years old with a diagnosis of ITP, pl <30x10^9/L AND a clinical need for first line treatment.
Key exclusion criteria	• Pregnant or lactating women<br>• HIV, Hepatitis B or C, or Common Variable immunodeficiency.<br>• Patients (men and women) unwilling to follow contraceptive advice if allocated to MMF treatment arm<br>• Contraindications to MMF or steroid (see SPC) including patients with hypersensitivity to mycophenolate mofetil, mycophenolic acid or to any of the excipients
Planned Sample Size	120
Follow up duration	12 to 24 months
Planned Trial Period	36 months
Trial interventions	Standard steroid vs Steroid + Mycophenolate, MMF (500mg orally bd increasing up to 1g bd if tolerated and tapered at 6m if in complete remission)
Primary objective	To test the hypothesis that for patients with newly diagnosed Immune thrombocytopenia (ITP), Mycophenolate (MMF) commenced first line with steroids is a more effective treatment pathway than the current standard care of steroids alone first line.
Primary outcome	Time from randomisation to treatment failure:<br>Refractory patients (pl <30x10^9/L in spite of 2 weeks treatment in the steroid arm or pl <30x10^9/L in spite of 2 months treatment in the steroid +MMF arm)

For peer review only - http://bmjopen.bmj.com/site/about/guidelines.xhtml

Centre for Trials Research
Canolfan Ymchwil Treialon

	or who initially respond but then relapse (defined clinically as pl <30x10^9/L and need for further therapy).
Secondary outcomes	1. Remission rates (pl >30 x10^9/L and at least 2 fold increase from baseline). Complete >100x10^9/L, partial 30-100x10^9/L<br>2. Time to relapse<br>3. Time to next therapy<br>4. Cumulative steroid dose<br>5. Bleeding events<br>6. Need for rescue therapies<br>7. Need for splenectomy<br>8. Time off work<br>9. Number of hospital admissions, day unit and clinic attendances<br>10. Fatigue<br>11. Quality of life<br>12. Medication side effects, toxicity or other adverse events (including infection episodes)<br>13. NHS costs<br>14. Personal and social costs
Ancillary translational research study	Performed at the University of Bristol and UHBristol<br>Funded by various charitable sources
Sample Storage	Bristol Biobank, Level 7, Bristol Royal Infirmary, Upper Maudlin Street<br>Bristol, BS2 8AE<br>Telephone: 0117 342 3190/07813 344 577<br>E-mail: Bristol-biobank@bristol.ac.uk<br>NHS REC 14/WA/1253<br>Licence no 12512

For peer review only - http://bmjopen.bmj.com/site/about/guidelines.xhtml

LIST of CONTENTS

GENERAL INFORMATION	Page No.
TITLE PAGE	1
SIGNATURE PAGE	2
AMENDMENT HISTORY AND KEY TRIAL CONTACTS	3-6
TRIAL SUMMARY	7-8
CONTENTS	9-11
LIST OF ABREVIATIONS	12-13
SECTION	
1. TREATMENT PATHWAYS 1 & 2	14-15
2. PLAIN ENGLISH SUMMARY	16
3.BACKGROUND AND RATIONALE	16-18
4. TRIAL DESIGN	19
5. STUDY SETTING	19
6. OBJECTIVES AND OUTCOME MEASURES/ENDPOINTS	19
6.1 Primary objective	19
6.2 Secondary objectives	19-20
6.3 Primary endpoints/outcomes	20
6.4 Secondary endpoints/outcomes	20
7. ELIGIBILITY CRITERIA	21
7.1 Inclusion criteria	21
7.2 Exclusion criteria	21-22
8. TRIAL PROCEDURES	22
8.1 Risk Assessment	22
8.2 Site Selection	22-23
8.3 Recruitment	23
8.3.1 Patient Identification	24
8.3.2 Screening	24
8.4 Consent	24
8.4.1 Additional Consent Provisions	25
8.5 Randomisation Scheme	25
8.5.1. Method of implementing the allocation sequence	25-26
8.6 Trial Interventions	26-27
8.7 Baseline Data Collection	27
8.8 Trial Assessments	27
8.9 Specific study follow up visits	27-28
8.10 End of trial assessment	28
8.11 Withdrawal criteria	28
8.12 Lost to follow up	29
8.13 Storage and analysis of samples	29
8.14 End of trial	30
9. TRIAL INTERVENTION	30

For peer review only - http://bmjopen.bmj.com/site/about/guidelines.xhtml

Cardiff University Centre for Trials Research / Prifysgol Caerdydd Canolfan Ymchwil Treialon — NHS National Institute for Health Research — itp — University Hospitals Bristol NHS Foundation Trust

9.1 MMF	30
9.2 Legal Status of drug	30
9.3 Drug storage and supply	30
9.4 Dosage modifications	30
9.5 Interacting drugs	31
9.6 Trial Restrictions	31
9.7 Assessment of Compliance	31
9.8 Accountability procedures	31
9.9 Non-intervention Corticosteroids	31-32
**10. PHARMACOVIGILANCE**	32
10.1 Definitions	32-33
10.2 Trial Specific SAE reporting requirements	33
10.3 Causality	33-34
10. 4 Expectedness<br>Table 1: Reference Safety Information to be used for Expectedness Assessments<br>Summary of Product Characteristics (SPC)	34
10.5 Reporting procedures	35
10.5.1 Participating Site Responsibilities	35-36
10.5.2 CTR Responsibilities	36
10.6 SUSAR reporting	36
10.7 Notification of deaths	37
10.8 Overdose	37
10.9 Contraception and Pregnancy reporting whilst participating in the trial	37
10.10 Reporting urgent safety measures	38
**11. STATISTICS AND DATA ANALYSIS**	38
11.1 Sample size	38
11.2 Statistical analysis	38-39
11.3 Interim analysis	39
11.4 Procedure(s) to account for missing or spurious data	39
11.5 Economic evaluation	39-40
**12. DATA HANDLING**	40
**13. QUALITY CONTROL & ASSURANCE**	41
13.1 Monitoring, Audit and Inspections	41
13.2 Quality Control	41
**14. ETHICAL AND TRIAL ADMINISTRATION**	41-42
14.1 Peer Review	42
14.2 Public and Patient Involvement	42
14.3 Protocol compliance	43
14.4 Data protection and patient confidentiality	43
14.5 Financial considerations	43
14.6 Indemnity	44
**15 TRIAL MANAGEMENT**	44

For peer review only - http://bmjopen.bmj.com/site/about/guidelines.xhtml

15.1 Trial Management Group (TMG)	44
15.2 Trial Steering Committee (TSC)	44
15.3 Data Monitoring Committee (DMC)	44
16. ANCILLARY TRANSLATIONAL RESEARCH SUB-STUDY	44-45
17. DISSEMINATION POLICY	45
18. ARCHIVING	45
19. REFERENCES	46-48
20. APPENDICIES	49-69
Appendix 1: Schedule of Procedures	49
Appendix 2: Mycophenolate Mofetil (web link to SPC)	50
Appendix 3: Prednisolone (web link to SPC)	50
Appendix 4: Dexamethasone (web link to SPC)	50
Appendix 5: Common Terminology Criteria for Grading of Adverse Events (web link)	50
Appendix 6: Quality of Questionnaires:<br>FACT-Th6 (V4)<br>ICECAP V2 - A Measure<br>SF-36v2 Health Survey<br>FACIT-F V4, pg 3 FATIGUE<br>Thrombocytopenia care costs	51-67

For peer review only - http://bmjopen.bmj.com/site/about/guidelines.xhtml

Cardiff University / Prifysgol Caerdydd — Centre for Trials Research / Canolfan Ymchwil Treialon; NHS National Institute for Health Research; itp; University Hospitals Bristol NHS Foundation Trust

LIST OF ABBREVIATIONS

AE	Adverse Event
AR	Adverse Reaction
bd	Twice daily
CI	Chief Investigator
CRF	Case Report Form
CTA	Clinical Trial Authorisation
CTCAE	Common Terminology Criteria for Grading of Adverse Events
CTIMP	Clinical Trial of Investigational Medicinal Product
DMC	Data Monitoring Committee
EUCTD	European Clinical Trials Directive
EudraCT	European Clinical Trials Database
GCP	Good Clinical Practice
ICF	Informed Consent Form
ICH	International Conference on Harmonisation of technical requirements for registration of pharmaceuticals for human use.
IMP	Investigational Medicinal Product
ISF	Investigator Site File
ISRCTN	International Standard Randomised Controlled Trials Number
ITP	Immune Thrombocytopenia
MA	Marketing Authorisation
MHRA	Medicines and Healthcare products Regulatory Agency
MMF	Mycophenolate Mofetil
NHS R&D	National Health Service Research & Development
od	Once daily
PI	Principal Investigator
PIC	Participant Identification Centre
PIS	Participant Information Sheet
QA	Quality Assurance
QC	Quality Control
RCT	Randomised Control Trial

For peer review only

For peer review only - http://bmjopen.bmj.com/site/about/guidelines.xhtml

REC	Research Ethics Committee
SAE	Serious Adverse Event
SAR	Serious Adverse Reaction
SDV	Source Data Verification
SOP	Standard Operating Procedure
SPC	Summary of Product Characteristics
SSI	Site Specific Information
SUSAR	Suspected Unexpected Serious Adverse Reaction
TMG	Trial Management Group
TSC	Trial Steering Committee
TMF	Trial Master File
TSF	Trial Site File

For peer review only - http://bmjopen.bmj.com/site/about/guidelines.xhtml

Cardiff University Centre for Trials Research / Prifysgol Caerdydd Canolfan Ymchwil Treialon — NHS National Institute for Health Research — itp — University Hospitals Bristol NHS Foundation Trust

1. TREATMENT PATHWAYS 1 & 2

Flight study: Pathway 1: Steroid alone first line

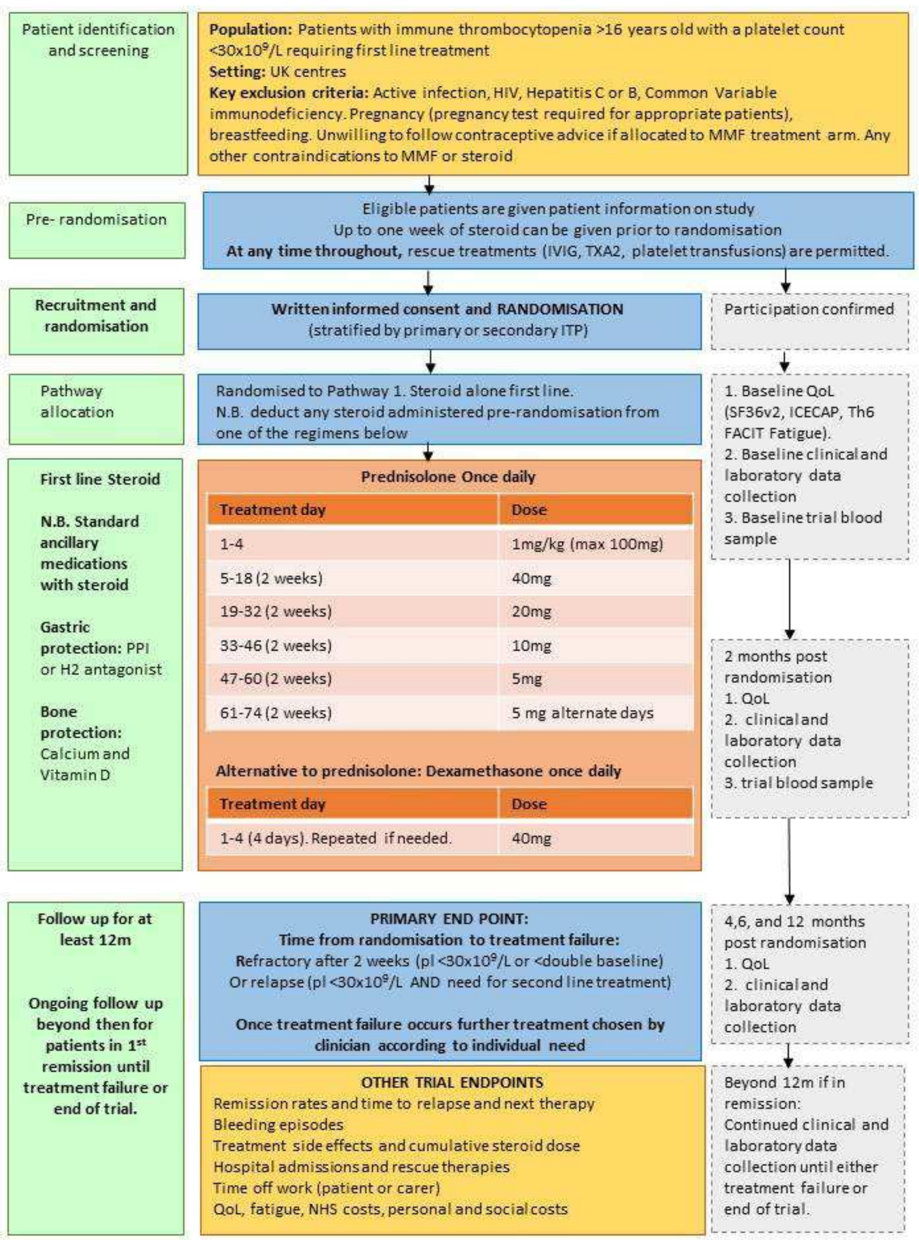

Cardiff University Centre for Trials Research / Prifysgol Caerdydd Canolfan Ymchwil Treialon
NHS National Institute for Health Research
itp
University Hospitals Bristol NHS Foundation Trust

Flight study: Pathway 2 : Steroid and MMF first line

Patient identification and screening

Population: Patients with immune thrombocytopenia >16 years old with a platelet count <$30x10^9$/L requiring first line treatment
Setting: UK centres
Key exclusion criteria: Active infection, HIV, Hepatitis C or B, Common Variable immunodeficiency. Pregnancy (pregnancy test required for appropriate patients), breastfeeding. Unwilling to follow contraceptive advice if allocated to MMF treatment arm. Any other contraindications to MMF or steroid

Pre-randomisation

Eligible patients are given patient information on study
Up to one week of steroid can be given prior to randomisation
At any time throughout, rescue treatments (IVIG, TXA2, platelet transfusions) are permitted.

Recruitment and randomisation

Written informed consent and RANDOMISATION
(stratified by primary or secondary ITP)

Participation confirmed

Pathway allocation

Randomised to Pathway 2. Steroid AND MMF first line.
N.B. deduct any steroid administered pre-randomisation from one of the regimens below

1. Baseline QoL (SF36v2, ICECAP, Th6 FACIT Fatigue).
2. Baseline clinical and laboratory data collection
3. Baseline trial blood sample

First line steroid AND MMF

N.B. Standard ancillary medications with steroid

Gastric protection: PPI or H2 antagonist

Bone protection: Calcium and Vitamin D

Prednisolone once daily

Treatment day	Dose
1-4	1mg/kg (max 100mg)
5-18 (2 weeks)	40mg
19-32 (2 weeks)	20mg
33-46 (2 weeks)	10mg
47-60 (2 weeks)	5mg
61-74 (2 weeks)	5 mg alternate days

Alternative to prednisolone: Dexamethasone once daily

Treatment day	Dose
1-4 (4 days). Repeated if needed.	40mg

MMF twice daily

Treatment day	dose
1-14 (2 weeks)	500mg
15-28 (2 weeks)	750mg
From d29	1g

At 6m if pl>$100x10^9$/L	Reduce MMF by 250mg increments each month
Aim	Lowest dose to achieve pl >50 without bleeding

2 months post randomisation
1. QoL
2. clinical and laboratory data collection
3. trial blood sample

Follow until the end of trial (minimum 12m)

PRIMARY END POINT:
Time from randomisation to treatment failure:
Refractory after 2 months (pl <$30x10^9$/L or <double baseline)
Or relapse (pl <$30x10^9$/L AND need for second line treatment)

Once treatment failure occurs further treatment chosen by clinician according to individual need

4,6, and 12 months post randomisation
1. QoL
2. clinical and laboratory data collection

OTHER TRIAL ENDPOINTS
Remission rates and time to relapse and next therapy
Bleeding episodes
Treatment side effects and cumulative steroid dose
Hospital admissions and rescue therapies
Time off work (patient or carer)
QoL, fatigue, NHS costs, personal and social costs

Beyond 12m if in remission:
Continued clinical and laboratory data collection until either treatment failure or end of trial.

For peer review only - http://bmjopen.bmj.com/site/about/guidelines.xhtml

2. PLAIN ENGLISH SUMMARY

Immune thrombocytopenia (ITP) is an illness that causes bruising and bleeding due to a low platelet count (blood cells essential for normal clotting). As treatment, patients are first given high dose steroids but most suffer side effects (e.g. difficulty sleeping, weight gain, moods swings, high blood pressure, diabetes etc). In addition, the illness doesn't get better for some patients, and the majority of others get ill again when the steroids are stopped. Only about 20% stay well long term. Mycophenolate (MMF) is often used as the next treatment for ITP and it works quite well. However, it can take up to 2 months to work during which patients continue to be at risk of bleeding, bruising, feeling tired and usually need more steroids which they find intolerable. They are required to come to hospital each week for blood tests and many need to take time off work. We want to find out whether more patients would feel better sooner if everyone takes MMF at diagnosis instead of what we do at the moment (waiting for the illness to come back). We plan to test this by comparing the current way we treat patients to a new way with patients given MMF right at the start of their treatment. Patients from different hospitals will be asked to take part and half will be randomly chosen for the new pathway. Their normal hospital appointments will be used to record how well they are for a year, therefore no extra appointments will be required as a result of agreeing to take part. We have a group of local patients in Bristol who have helped us and we will continue to meet with them throughout. We expect the results will be able to improve care for patients with ITP within 5 years.

3. BACKGROUND AND RATIONALE

Immune thrombocytopenia (ITP) has an incidence of 2.9/100,000 person-years (Moulis et al 2014). It is an autoimmune condition that presents with bleeding and bruising due to a low platelet count. In ITP, there is increased consumption and reduced production of platelets due to both antibody and cell mediated autoimmune attack of platelets and megakaryocytes involving dysregulated autoreactive T and B cell lymphocytes (McKenzie et al 2013, Iraqi et al 2015, Ji et al 2014, Audia et al 2014, Hu et al 2015, Yu et al 2015, Kuwana et al 2014, Li et al 2015, Hua et al 2014, Ma et al 2014, Cines et al 1983, Nishimoto et al 2013).

Current first line treatment following ITP diagnosis is with high dose steroids but this has several downsides. First, the majority of patients suffer significant side effects including mood swings, difficulty sleeping, weight gain, high blood pressure, diabetes, gastric irritation, skin thinning and osteoporosis. A published survey of ITP patients reported 98% had at least one side effect and 38% stopped or reduced dosage due to intolerable side effects (Brown et al 2012). In the UK ITP registry, the most frequently reported co-morbidities were related to steroids and correlated with duration of treatment (hypertension in 30%, diabetes in 19%) (Newland A et al, poster BSH 2015). The second problem is that patients are heterogeneous in their response to steroid with some (20%) not responding at all and the majority of others (70%-90%) relapsing when the steroids are reduced or stopped (Provan et al 2010, Neunert et al 2011). Patients who are refractory or relapse (the majority), remain at risk of bleeding/bruising, which occasionally can be severe including intracranial haemorrhage (Neunert et al 2015). They often receive more steroid with associated side effects. Some require hospital admission and expensive rescue therapies (e.g. IVIG for a 70kg patient=£3,906). They continue to require frequent blood tests and doctor visits and are usually unable to continue their normal activities until their illness is controlled. Fatigue

For peer review only - http://bmjopen.bmj.com/site/about/guidelines.xhtml

is also associated with disease activity and can be severe (Hill et al 2015). Physical factors combine with psychological stress through fear of bleeding, need for time off work and lifestyle restrictions due to bleeding risk to adversely impact quality of life (Snyder et al 2008, Tarantino et al 2010).

First line treatment for ITP is unsatisfactory but it has been unchallenged for decades. The lack of new approaches has arisen from a chronic lack of research funding and clinical trials. The few trials done have been funded by pharmaceutical companies, risking publication bias towards high cost non-generic drugs. The relative rarity (2.9/100,000 person-years), non-cancerous nature and rare impact on survival of ITP have prevented it being a priority for research funding in the past. However, this underestimates the profound adverse impact a diagnosis of ITP and its treatment can have for individual patients, many of whom are young. There is also a costly financial impact for the NHS from the healthcare resources patients require when their illness is uncontrolled. In addition, the problems faced by patients with ITP mirror those with other autoimmune conditions which as a group are common, affecting 3% of the population. There is an urgent clinical need to address this inequality, improving first line treatment for ITP through high quality, independently funded research to allow patients with this condition access to improvements in care seen in other illnesses such as cancer or heart disease.

Current popular options for second line or subsequent treatment include Mycophenolate, Rituximab, Thrombopoeitin mimetics and splenectomy. Splenectomy is an effective treatment (60% long term remission rates) but international guidelines recommend deferring splenectomy for the first 12 months following diagnosis due to the chance of spontaneous remission (risk of unnecessarily removing a healthy organ) (Provan et al 2010, Neunert et al 2011). Surgical operations are not popular with patients and there is increasing awareness of the short and long term complications of splenectomy including infection, bleeding, arterial and venous thrombosis, cancer and relapse (Kristinsson et al 2014). The splenectomy numbers performed in the UK has dramatically reduced over recent years (ITP registry data). Rituximab is a monoclonal antibody treatment which targets antibody production by B cells. It is relatively expensive, with disappointing long term remission rates which are similar to placebo (Ghanima et al 2015, Gudbrandsdottir et al 2013, Psaila et al 2015). Thrombopoeitin (TPO) mimetics stimulate platelet production, are well tolerated and effective in the majority (Rodeghiero et al 2015) but at significant financial cost, prohibiting widespread use in the UK for early treatment (NICE guidance). A small (n=12) non-randomised study using TPO mimetic with corticosteroid first line showed efficacy but perhaps less than expected (Gomez-Almaguer 2014). By contrast, Mycophenolate (MMF) is a widely used second line agent in the UK due to good efficacy (response rates of 50-80%), safety and tolerability profile (Miano et al 2016, Taylor et al 2015, Kotb et al 2005, Provan D 2006, Hou et al 2003, Colovic et al 2011, Zhang et al 2005, Howard et al 2002). Mycophenolate has activity against both autoreactive T and B cells and has also shown efficacy in refractory ITP including corticosteroid resistant patients suggesting a complimentary mechanism of action (Colovic et al 2011). It is less expensive to the NHS than some other second line options costing approximately £182/year (generic cost) compared to costs for average doses of romiplostim at £25 000/year, Eltrombopag £20 000/year or rituximab at £8000 for a course of 4 infusions. However, similar to other second line therapies, MMF has a relatively slow (up to 2 months) onset of action. In the meantime, patients often receive further steroid (to maintain a "safe platelet count") and continue to suffer problems associated with their illness (see above).

Direct feedback from patients regarding the difficulties they face in the first months following ITP diagnosis has been the primary driving force for this research proposal. Local (Bristol) and national patient groups (ITP support

For peer review only - http://bmjopen.bmj.com/site/about/guidelines.xhtml

association) have been fundamental to the formulation of patient relevant priorities for treatment. Patients report that avoidance of relapse, early achievement of a stable platelet count, reduced overall steroid dose and reduced hospital attendances are the most important goals for ITP management from their perspective. We are proposing the first UK, NHS coordinated, Pharma independent multicentre randomised controlled trial, testing a "common sense/practical" new approach using MMF first line with the aim of preventing the almost inevitable first relapse. By giving patients a stable platelet count sooner we expect to improve other outcomes such as quality of life and fatigue. Potential indirect benefits to the NHS include reduced need for rescue treatments, hospital attendances and admissions and reduced need for high cost treatments such as TPO mimetics. However, there will be some patients who will be treated with MMF who may have been successfully treated with steroids alone (10-30%). Similar to other immunosuppressive treatments, MMF may slightly increase infection and cancer risk with long term use. This puts the trial in equipoise. The proposal includes a strategy to reduce and stop MMF at 6 months for patients in complete remission to prevent unnecessary long term use.

This trial proposal has received support from clinicians and patients nationally (UK ITP forum and ITP support association). To ensure objective and meaningful outcomes, it will be a multicentre RCT comparing MMF with steroid vs steroid alone for first line ITP treatment, expecting to recruit 120 patients (expecting 100 full datasets). Patients can be given up to one week of steroid prior to randomisation to enable sufficient time to read information, discuss and ask questions with informed consent in an appropriate setting. Patients will receive the usual follow up according to clinical need and local policy. Laboratory and clinical data will be collected from routine appointments. In addition, patient oriented outcomes will be recorded at diagnosis, 2, 4, 6, and 12 months using validated patient questionnaires. Consent to additional blood samples for translational research studies will be optional (time 0 and 2 months).

The choice of this open label design was made both in order to allow true patient treatment costs to be calculated for the cost effectiveness analysis, and to deal with the complexities of placebo controlling a drug that needed titrating at the start and tapering at the end. In addition, over encapsulation was only possible for the lower MMF dose (250 mg) and the resulting capsule was the largest size which would mean most patients taking 8 large capsules per day in both arms. Following discussion with patients in Bristol, this was something that would put them off taking part in the study. Patients were clear that from their perspective the straight forward open label design would be preferable and was easier for a new patient to understand, increasing likelihood of consent (it was recognised that these patients sometimes feel overwhelmed already with the impact of a new diagnosis and simplicity was a priority). The quotes from 2 separate companies also showed the financial costs of encapsulation to generate a placebo were prohibitively expensive (>£100,000 excluding VAT for 80 patients).

Patients in Bristol supported the proposed trial design, end points and data collection and encouraged inclusion of patient oriented outcomes (quality of life, time off work). They suggested that any extra blood tests needed for the trial and data collection should be done during usual care appointments. These suggestions have been incorporated into the design. This will be the first UK multicentre ITP RCT and if successful represents a platform for future trials in ITP and other non-cancerous rare disorders.

For peer review only - http://bmjopen.bmj.com/site/about/guidelines.xhtml

4. TRIAL DESIGN

A multicentre, open label, randomised clinical trial of MMF with steroid as first line treatment for patients with ITP against the standard care pathway of steroids alone as first line treatment.

5. STUDY SETTING

Haematology departments in hospitals (secondary care) across the UK where ITP patients are treated.

The trial is expected to include 120 patients with a diagnosis of ITP receiving first line treatment. We will select approximately 30 of the highest throughput centres to set up the trial. The trial processes will be run by the Centre for Trials Research, Cardiff University and sponsored by University Hospital Bristol NHS Foundation Trust.

6. AIMS, OBJECTIVES AND OUTCOME MEASURES/ENDPOINTS

The overarching aim of this study is to test the hypothesis that for patients with Immune thrombocytopenia (ITP) requiring first line treatment, Mycophenolate (MMF) commenced with steroids is a more effective treatment pathway than the current standard care of steroids alone.

We would expect survival free from treatment failure to be longer when MMF is used with corticosteroid first line than with steroid alone. We also aim to test whether this is a safe and well tolerated approach and whether other outcomes are improved:

- Remission rates
- Patient oriented outcomes (quality of life, fatigue, time off work)
- Cumulative steroid dose
- Hospital attendances and admissions
- Bleeding episodes
- Need for rescue and second line treatment

6.1 Primary objective

To demonstrate which first line treatment pathway helps patients with ITP achieve a stable platelet count sooner, measured as survival free from treatment failure (time from randomisation to treatment failure).

To assess this question definitively in a fully powered multicentre randomised controlled trial of the two different treatment pathways in patients followed up for a minimum of 12 months from randomisation.

6.2 Secondary objectives

The secondary objectives include:

- Collecting data on time to treatment failure as measured objectively by a fall in platelet count to $<30x10^9/L$ and need for new treatment.
- Collecting clinical data on additional treatments (including "rescue treatments" such as IVIG).

For peer review only - http://bmjopen.bmj.com/site/about/guidelines.xhtml

- Collecting clinical data on cumulative steroid dose.
- Collecting clinical data on additional investigations, hospital appointments and admissions.
- Collecting patient reported outcomes through standardised questionnaires.
- Collecting resource use data relating to an NHS/PSS and societal perspective.
- Collecting data on treatment side effects and adverse events (including bleeding, infections and steroid side effects)

For peer review only

6.3 Primary endpoint/outcome

The primary outcome is time from randomisation to treatment failure (Rodeghiero et al 2009). This will include patients who are refractory (pl <$30x10^9$/L in spite of 2 weeks treatment in the steroid arm or pl <$30x10^9$/L in spite of 2 months treatment in the steroid +MMF arm) or who initially respond but then relapse (defined clinically as pl <$30x10^9$/L and need for further therapy).

Hospital monitoring of platelet levels is part of routine care for ITP patients and we will collect these details from the medical notes without requiring patients to come in for additional samples to be taken. These locally collected samples may be collected monthly (or less often) for patients believed to be in stable remission and weekly at lower or declining platelet levels. We expect this to allow us to calculate the time in remission and time in relapse with reasonable accuracy over the 12 to 24 month follow up period. In addition to clinical data taken from medical records, we will also ask the patients to complete questionnaires on fatigue, quality of life and bleeding scores at baseline, 2, 6, and 12 months.

6.4 Secondary endpoints/outcomes

1. Remission rates (pl >30 $x10^9$/L and at least 2 fold increase from baseline). Complete >$100x10^9$/L, partial 30-$100x10^9$/L
2. Time to relapse and time to next therapy
3. Cumulative steroid dose
4. Bleeding events
5. Need for rescue therapies
6. Need for splenectomy
7. Time off work
8. Number of hospital admissions, day unit and clinic attendances
9. Fatigue and Quality of life
10. Medication side effects, toxicity or other adverse events (including infection episodes)
11. NHS costs and Personal and social costs

Patient reported outcomes will be captured by the following questionnaires:

1. SF36v2 (Your health & wellbeing) – Quality of life (the license for this questionnaire is currently being purchased)
2. FACIT-Fatigue (version 4) – Fatigue
3. FACT-Th6 (version 4)- Bleeding
4. ICECAP-A V2- Quality of life
5. Health economic/resource use questionnaire - Personal and social costs

For peer review only - http://bmjopen.bmj.com/site/about/guidelines.xhtml

7. ELIGIBILITY CRITERIA

7.1 Inclusion criteria

- Patients (males and females) >16 years old with a diagnosis of ITP, a pl count <30x10^9/L AND a clinical need for first line treatment.
- Patients have provided written informed consent

7.2 Exclusion criteria

- The exclusion criteria include pregnancy and breastfeeding
- Patients with HIV, Hepatitis B or C, or Common Variable immunodeficiency.
- Women of child bearing potential require a pregnancy test result within 7 days prior to randomisation (as per 7.1 below) to rule out unintended pregnancy
- Contraindications to MMF or corticosteroid (see SPC, Appendix 2) including patients with hypersensitivity to mycophenolate mofetil, mycophenolic acid or to any of the excipients or active significant infection
- Patients not capable of giving informed consent (e.g. due to incapacity)
- Patients unwilling to follow contraceptive advice if allocated to MMF treatment arm (this information must be confirmed prior to randomization):

i. **WOMEN**: Because of the genotoxic and teratogenic potential of MMF, **women** of childbearing potential must use two reliable forms of contraception simultaneously before starting MMF, during therapy and six weeks after stopping MMF (hormonal or barrier method of birth control; abstinence). Contraceptive methods that can achieve a failure rate of less than 1% per year when used consistently and correctly are considered as highly effective birth control methods. Such methods include:

ii.
- combined (oestrogen and progestogen containing) hormonal contraception associated with inhibition of ovulation:
  - oral
  - intravaginal
  - transdermal
- progestogen-only hormonal contraception associated with inhibition of ovulation
  - oral
  - injectable
  - implantable*
- intrauterine device (IUD)*
- intrauterine hormone-releasing system (IUS)*
- bilateral tubal occlusion*

For peer review only - http://bmjopen.bmj.com/site/about/guidelines.xhtml

- vasectomised partner*
- sexual abstinence defined as refraining from heterosexual intercourse during the entire period of risk associated with the trial treatments.

These contraception methods are considered to be low user dependency.

NB: Women are considered not of child bearing potential if they are surgically sterile (i.e. they have undergone a hysterectomy, bilateral tubal ligation, or bilateral oophorectomy) or they are postmenopausal.

iii. **MEN:** Sexually active men with female partners that are potentially child bearing are recommended to use condoms during treatment and for at least 90 days after cessation of treatment. Condom use applies for both reproductively competent and vasectomised men, because the risks associated with the transfer of seminal fluid also apply to men who have had a vasectomy. In addition, female partners of male patients treated with MMF are recommended to use highly effective contraception during treatment and for a total of 90 days after the last dose of MMF.

8. TRIAL PROCEDURES

8.1 Risk Assessment

A Trial Risk Assessment has been completed to identify the potential hazards associated with the trial and to assess the likelihood of those hazards occurring and resulting in harm. This risk assessment has been completed in accordance with the MRC/DH/MHRA Joint project guidance document 'Risk-adapted approaches to the management of Clinical Trials of Investigational Medicinal Products' and includes:

- The known and potential risks and benefits to human subjects
- How high the risk is compared to normal standard practice
- How the risk will be minimised/managed

This trial has been categorised as a Type A where the level of risk is no higher than the risk of standard medical care. A copy of the trial risk assessment may be requested from the Trial Manager. The trial risk assessment is used to determine the intensity and focus of monitoring activity.

8.2 Site Selection

The study has been widely publicised through UK ITP Forum, CRNs and the CI has advertised the study at the British Society for Haematology. Potential savings to sites for treatment costs may be an incentive to take part. Sites have also indicated that this would be a relatively straightforward study to set up and run.

For peer review only - http://bmjopen.bmj.com/site/about/guidelines.xhtml

This trial will be carried out at approximately 30 participating sites within the UK. All sites who are interested in participating in the trial will be required to complete a registration form to confirm that they have adequate resources and experience to conduct the trial.

- A signed Trial Agreement, including MTA for the translational component
- Current Curriculum Vitae and GCP training certificate of the Principal Investigator (PI)
- Completed Site Delegation Log and Roles and Responsibilities document
- Full contact details for all host care organisation personnel involved, indicating preferred contact
- A copy of the most recent approved version of the Participant Information Sheet(s) and Consent Form(s) on host care organisation headed paper
- A copy of the most recent approved GP letter on host care organisation headed paper
- A set of laboratory normal ranges and laboratory certification/accreditation from the host care organisation laboratory being used for analyses
- Returned copy of the Self-Evident Correction Log signed by the PI.

Upon receipt of all the above documents, the Trial Manager will send written confirmation to the Principal Investigator/lead Research Nurse detailing that the centre is now ready to recruit participants into the trial. This letter/email must be filed in each site's Site File. Along with the written confirmation, the site should receive a trial pack holding all the documents required to recruit into the Trial.

Occasionally during the trial, amendments may be made to the trial documentation listed above. CTR will issue the site with the latest version of the documents as soon as they become available. It is the responsibility of the CTR to ensure that they obtain local R&D approval for the new documents.

Site initiation will be by teleconferencing.

8.3 Recruitment

At each hospital haematology centre, patients with a diagnosis of ITP will be assessed for eligibility for the trial. Patients will be given information on the trial and allowed ample time to read this, discuss it with healthcare professionals, family members and given the opportunity to ask questions. Up to one week of steroid may be used prior to randomisation to allow the patients enough time to consider trial participation without compromising either their health or the therapeutic effect of MMF as a first line treatment. Informed consent will be taken by the local clinical investigator or specialist nurse in the appropriate setting following good clinical practice guidelines.

A screening log of all ineligible and eligible but not consented/not approached will be kept at each site so that any biases from differential recruitment will be detected. The screening log will capture reasons for ineligibility. When at site, logs may contain identifiable information but this must be redacted prior to being sent to CTR. The screening log should be sent to the Trial Manager on request.

For peer review only - http://bmjopen.bmj.com/site/about/guidelines.xhtml

8.3.1. Patient identification

- Identification will involve reviewing and screening the identifiable personal information of patients, undertaken by members of the normal clinical team.
- Eligible patients will be identified by a Haematology Consultant or Specialist Registrar before or soon after the patient is commenced on first line steroid.
- Patients' notes (history and examination) and results of investigations (hospital computer records) will be used to identify patients.

8.3.2 Screening

ITP is a diagnosis of exclusion and investigation should follow consensus guidelines (Provan et al 2010).

- Clinical information
- laboratory tests should follow consensus guidance (including blood film, HIV, Hepatitis B and C serology, Immunoglobulins, liver and renal function, LDH)
- Pregnancy test if woman with child bearing potential (must be performed within 7 days prior to randomisation)

8.4 Consent

The Principal Investigator (PI) retains overall responsibility for the informed consent of participants at their site and must ensure that any person delegated responsibility to participate in the informed consent process is duly authorised, trained and competent to participate according to the ethically approved protocol, principles of Good Clinical Practice (GCP) and Declaration of Helsinki.

Informed consent must be obtained prior to the participant undergoing procedures that are specifically for the purposes of the trial, using Patient Information Sheet 1.

The right of a participant to refuse participation without giving reasons must be respected.

Patients can consent to enter the trial but decline to have additional bloods taken for the lab study including bio banking for future research.

The participant must remain free to withdraw at any time from the trial without giving reasons and without prejudicing his/her further treatment and must be provided with a contact point where he/she may obtain further information about the trial. Where a participant is required to re-consent or new information is required to be provided to a participant it is the responsibility of the PI to ensure this is done in a timely manner.

A person is assumed to have the mental capacity to make a decision unless it is shown to be absent. Mental capacity is considered to be lacking if, in a specific circumstance, a person is unable to make a decision for him or herself because of impairment or a disturbance in the functioning of their mind or brain. Participants with mental incapacity are excluded from participating.

Where a participant is able to consent but later becomes incapacitated, the patient will be withdrawn from further trial procedures and standard medical care will be followed. However, the data collected up to the point

For peer review only - http://bmjopen.bmj.com/site/about/guidelines.xhtml

of incapacity and follow up data not requiring patient intervention will be recorded and retained as the original consent given endures the loss of capacity.

8.4.1 Additional consent provisions for collection and use of participant data and biological specimens in ancillary studies (Patient Information Sheet 2)

- Clinical data and biological specimens for ancillary studies will be acquired and stored during the trial.
- Participation in the ancillary research is not required for participation in trial and participants may opt out but still be included in the main study
- Following consent, if a subject withdraws from the ancillary research further samples, data will not be collected and if they choose, data collected prior to withdrawal will be destroyed
- Samples ready collected will not be used further in the trial and will be destroyed according to locally approved practices. Any results derived from the samples that have already been used prior to the withdrawal of consent will continue to be used in the trial.

8.5 The randomisation scheme

Patients who agree to participate will be randomised to MMF with corticosteroid or corticosteroid alone in a 1:1 ratio using a web based randomisation system based at Cardiff CTR. Due to the large number of centres and the small number of patients it will not be sensible to stratify randomisation by study centre, however randomisation will be blocked using random block sizes of 6 and 8 to ensure an even spread of patients across time and to retain concealment.

8.5.1 Method of implementing the allocation sequence

The sequence of allocation will be generated in advance using random block sizes in a Cardiff University validated randomisation system. The sequence will be kept hidden from all recruiting staff and only released patient by patient as patients are randomised. Throughout the study the list of randomised patient IDs and their allocation will be available to the trial statistician, and only recruiting research staff following last patient follow-up. The trial is not blinded or placebo controlled, and so access to this list to identify an individual patient’s allocation is not considered a breach of protocol.

The randomisation will be provided by remote computerised web-based allocation and will be supported by telephone service during working hours should network failure occur.

The recruiting nurse or clinician will need to have the following details available:

Name, date of birth, date of ITP diagnosis, study site.

The treatment allocation will be confirmed to site staff and members of the trial team via e-mail. The patient will be given a copy of the relevant pathway and a copy of the pathway will be placed in the patient notes.

The randomisation system will comply with the Data Protection Act.

For peer review only - http://bmjopen.bmj.com/site/about/guidelines.xhtml

Randomisation/Database website address:

http://flight.sewtudb.cf.ac.uk/login/

8.6 Trial interventions (see flow charts)

<u>Steroid +MMF pathway:</u>

1mg/kg od prednisolone 4 days (maximum of 100mg), 40mg od 2 weeks, 20mg od 2 weeks, 10mg od 2 weeks, 5mg od 2 weeks then 5mg alternate days 2 weeks then stop*. For the duration of steroid, patients will get a PPI or H2 antagonist to protect against gastric bleeding and appropriate bone protection.

*Dexamethasone 20mg or 40mg daily for 4 days is an alternative option to prednisolone if deemed clinically more appropriate for individual circumstances.

Any steroid commenced prior to randomisation will be deducted from the regime above. Some degree of flexibility of steroid dose and duration may be needed for individual patients according to comorbidity, tolerability and other factors.

From randomisation (alongside steroid), MMF 500mg bd starting dose then increased to 750mg bd after 2 weeks if tolerated and 1g bd after another 2 weeks if tolerated (4 weeks after starting).

After 6 months of MMF therapy, all patients who have remained in complete remission (pl count> 100 x10^9/L) will reduce the dose by 250mg (one capsule) each month. The aim is to continue on the lowest dose that achieves a haemostatic (safe) platelet count (pl >30 x10^9/L) and to ensure that patients who have gone into a spontaneous remission do not continue to take the drug indefinitely.

<u>Steroid only group:</u>

1mg/kg od prednisolone 4 days (maximum of 100mg), 40mg od 2 weeks, 20mg od 2 weeks, 10mg od 2 weeks, 5mg od 2 weeks then 5mg alternate days 2 weeks then stop*. For the duration of steroid, patients will get a PPI or H2 antagonist to protect against gastric bleeding and appropriate bone protection.

*Dexamethasone 20mg or 40mg orally daily for 4 days is an alternative option to prednisolone if deemed clinically more appropriate for individual circumstances.

Any steroid commenced prior to randomisation will be deducted from the regime above. Some degree of flexibility of steroid dose and duration may be needed for individual patients according to comorbidity, tolerability and other factors.

For peer review only - http://bmjopen.bmj.com/site/about/guidelines.xhtml

Cardiff University Centre for Trials Research / Prifysgol Caerdydd Canolfan Ymchwil Treialon — NHS National Institute for Health Research — itp — University Hospitals Bristol NHS Foundation Trust

<u>In both groups:</u>
Importantly, emergency and rescue treatments will be permitted throughout the study. These include platelet transfusions, tranexamic acid and intravenous immunoglobulin. These are known not to impact on the natural history of ITP and it is recognised that they may be important for patient safety. The use of "rescue treatments" will be recorded on the CRF.

If treatment failure occurs, choice of second line treatment will be individualised according to patient's clinical circumstances. Further steroid will be given according to clinical need.

8.7 Baseline data collection

- Age
- Gender
- Date of diagnosis
- Date first line treatment commenced
- Date of randomisation
- Height
- Weight
- Blood pressure
- Clinical presentation (including bleeding history)
- Comorbidities
- Medications
- Primary or secondary ITP? If secondary, detail underlying cause?
- FBC and blood film results
- Immunoglobulins
- Hepatitis B, C and HIV serology results
- Other relevant investigations (if done e.g. Bone marrow results)
- Additional optional research blood samples will be sent to: *Bristol Biobank, Level 7, Bristol Royal Infirmary, Upper Maudlin Street, Bristol, BS2 8AE, Telephone: 0117 342 3190/07813 344 577*

8.8 Trial assessments

Patients will have routine bloods checked and hospital attendances according to local policy and clinical need.

Patients taking MMF should have FBC monitoring according to standard care. Please see Appendix 1 Schedule of Procedures

8.9 Specific study follow up visits

2m, 4m, 6m, 12m

- FBC results, bleeding episodes,

For peer review only - http://bmjopen.bmj.com/site/about/guidelines.xhtml

- Date of treatment failure (refractory or relapse AND need for second line therapy)
- Weight
- Blood pressure
-
- Medication side effects (including infections)
- Dose and duration of corticosteroids
- Need for rescue or other treatments (including second or third line)
- Hospital attendances or admissions
- Days off work
- Patient questionnaires: Quality of life assessment
- Immunoglobulins rechecked at 6 months and 12 months

8.10 End of trial assessment

12-24 m

In order to confirm the treatment failure endpoint has been reached, we will collect the platelet levels details from sites (as described in 6.3) after all their patients have completed their follow up visits at any given site to the end of the trial. Priority data to be collected would be platelet count data (remission, relapse) and treatment data (treatment taken or not taken).

8.11 Withdrawal criteria

Participants have the right to withdraw their consent from the study at any time for any reason. Should a patient decide to withdraw from the study, all efforts should be made to report the reason for withdrawal as thoroughly as possible. The investigator should ascertain from which aspects of the trial the patient wishes to withdraw and record the details in the appropriate part of the CRF. If a patient chooses to withdraw from treatment only, the patient should continue to be assessed in accordance with the protocol. If a patient wishes to withdraw from the trial (i.e. including trial-specific assessments) but is willing for further data to be supplied to CTR, then further routine follow-up data such as disease response status and further treatment, will continue to be supplied by the investigator to CTR according to the protocol.

The local investigator also has the right to withdraw patients from the study treatment for a number of justifiable reasons including unacceptable toxicity (an adverse event or events that, in the judgment of the investigator, may cause severe or permanent harm or which rule out continuation of the study drug), unforeseen events which in the opinion of the investigator make further treatment inadvisable, serious violation of the study protocol (including persistent patient non-attendance and non-compliance) or for clinical reasons not related to the study treatment. Patients withdrawn by the investigator should continue to undergo safety assessments as per the trial schedule and to receive standard care as per local policy.
Once the completed CRF has been received in CTR, a withdrawal note will be recorded on the participant's records and inform all relevant members of the Trial Team of the level and type of withdrawal and what information will no longer be collected for this participant. Any data received after the withdrawal date will be disposed of.

For peer review only - http://bmjopen.bmj.com/site/about/guidelines.xhtml

8.12 Lost to follow up

If patients are lost to follow up then their local centre should attempt to find out the reason for this by local sites contacting the patient and GP according to standard policy.

It may be possible for limited data collection to continue for patients lost to follow up (for example through their GP blood results or an alternative hospital if they are relocated or by telephone if they are unable to attend).

If possible, follow up data should continue to be collected to the end of the trial.

8.13 Storage and analysis of samples

- Bloods required for routine ITP standard of care should be collected, processed and destroyed locally according to site specific standard policy (e.g. FBC, immunoglobulins, virology)
- Patients who have consented for the laboratory sub-study (translational basic science research) will have additional bloods taken using the sample packs provided by the FLIGHT study team. These bloods will be taken at baseline and 2 months
- Ideally blood samples should be taken from Monday to Thursday and posted promptly the same day by Royal mail to: Bristol Biobank, Level 7, Bristol Royal Infirmary, Upper Maudlin Street, Bristol, BS2 8AE, Telephone: 0117 342 3190/07813 344 577, Email: Bristol-biobank@bristol.ac.uk
- Prior to sending, no sample processing by local laboratories is required and samples do not need refrigerating and can be kept at room temperature
- The form within the sample packs should be completed and the blood tubes labelled before they are enclosed together in the pre-paid sample packs and sent the same day Royal Mail in the safe packs provided
- These additional blood samples will be processed at the University of Bristol and stored in specialist freezers e.g. a -80°C +/- 10°C in Bristol Biobank (ethically approved) according to standard procedure
- Additional blood samples will be held in long-term storage for future unspecified use as outlined in patient information sheet.
- The additional blood samples will be destroyed following standard procedure.

It is the responsibility of the trial site to ensure that samples are appropriately labelled in accordance with the trial procedures to comply with the 1998 Data Protection Act. Biological samples collected from participants as part of this trial will be transported, stored, accessed and processed in accordance with national legislation relating to the use and storage of human tissue for research purposes and such activities shall at least meet the requirements as set out in the 2004 Human Tissue Act and the 2006 Human Tissue (Scotland) Act.

For peer review only - http://bmjopen.bmj.com/site/about/guidelines.xhtml

8.14 End of trial

The trial will end for a patient after they have completed their final study visit (12m from randomisation). The end of the trial is after all trial participants have completed their final study visit at 12m and follow up data (end of trial assessment data collected from sites (8.10) collected to the end of the trial, all data queries resolved, the database locked and the analysis completed.

9 TRIAL INTERVENTION

9.1 MMF

Mycophenolate Mofetil 500 mg and 250mg tablets for oral administration
Mycophenolate Mofetil Tablets should not be crushed.
The IMP is a generic drug, therefore any brand of the IMP can be used.

9.2 Legal status of the drug

Mycophenolate Mofetil is indicated in combination with Ciclosporin and corticosteroids for the prophylaxis of acute transplant rejection in patients receiving allogeneic renal, cardiac or hepatic transplants. Although commonly used for ITP treatment it is not licensed for this indication (as it is a rare illness, many treatments are not licensed for this indication).

9.3 Drug storage and supply

As this is an open label trial of different treatment pathways rather than a placebo controlled trial, the dispensing will all be carried out by the local hospital pharmacy after randomisation. The medication should be stored as described in the Summary of Product Characteristics.

Study medication will be stored and dispensed by the trial site's pharmacy department in accordance with Good Clinical Practice, Good Manufacturing Practice and pharmacy department SOPs.

9.4 Dosage modifications

Recommendation from the SPC: *Patients taking MMF should have a minimum of FBC weekly during the first month, twice monthly for the second and third months of treatment, then monthly through the first year. If neutropenia develops (absolute neutrophil count < 1.3 x* 10^9*/L), it may be appropriate to interrupt or discontinue MMF.*

No dose adjustments are needed for renal or liver impairment. The SPC states that if glomerular filtration rate < 25 mL/min/1.73 m2, these patients should be closely observed and doses greater than 1 g administered twice a day should be avoided.

For peer review only - http://bmjopen.bmj.com/site/about/guidelines.xhtml

9.5 Interacting drugs

Avoid: Live vaccines.

Other drug interactions for consideration but unlikely to be clinically significant in this RCT due to the MMF doses used and the indication (not solid organ transplant):

- Aciclovir, ciclosporin, ganciclovir, probenecid: Potential to slightly increase levels. At expected doses used in this RCT, this is not considered clinically significant.
- Antacids and proton pump inhibitors (PPIs), cholestyramine, temilsartan, Rifampicin, Sevelamer, ciprofloxacin, Augmentin: Potential to slightly reduce MMF levels.

9.6 Trial restrictions

MMF is teratogenic. Therefore, the most important restrictions apply to men and women to prevent conception and pregnancy whilst taking this medication (see exclusion criteria).

Standard of care is to clearly inform patients of risk of pregnancy, importance of pregnancy prevention and test for pregnancy prior to starting MMF; the latter only repeated if clinically indicated (e.g. gap in contraception). Sites will check patient contraception compliance at all visits for both males and females, this will be confirmed in the case report form. If any non-compliance with the contraception advice is discovered during the contraception check, then a pregnancy test will be performed on female participants. Male participants will be asked for their partners to have a pregnancy test performed and report the result to their local site.

9.7 Accountability Procedures

Local site pharmacies will be provided with a pharmacy manual which will include a trial specific accountability form. Pharmacies will also be asked to record batch numbers and expiry dates on the prescription when dispensing.

9.8 Assessment of compliance

- Compliance will be monitored and recorded at the routine hospital appointments.
- Patients who are non-compliant will remain in the study with the reason for non-compliance documented and recorded on the CRF.
- The reason for non-compliance should be addressed if possible. If due to medication side effects, this is an important secondary outcome. For other reasons, compliance should be encouraged with constructive advice on how this could be improved (e.g. phone alarm reminder).

9.9 Non Intervention - Corticosteroids

i. Prednisolone (Appendix 3)

ii. Dexamethasone (Appendix 4)

For peer review only - http://bmjopen.bmj.com/site/about/guidelines.xhtml

iii. Rescue therapies: Tranexamic acid, Intravenous human immunoglobulin (IVIG), platelet transfusions

All of these can be used according to clinical need.

10 PHARMACOVIGILANCE

The collection and reporting of Adverse Event (AEs) will be in accordance with the Medicines for Human Use Clinical Trials Regulations 2004 and its subsequent amendments and CTR SOPs. Definitions of different types of AE are listed below. Investigators should assess the seriousness and causality (relatedness) of all SAEs experienced with reference to the SPC. The Principal Investigator is responsible for ensuring that all site staff involved in this trial are familiar with the content of this section.

All SAEs must be reported immediately (within 24 hours of knowledge of the event) by personnel at the participating site to the CTR Pharmacovigilance & Safety Specialist unless the event is specified as not requiring immediate reporting (see section 10.4). This includes SAEs related to MMF and steroid.

10.1 Definitions

Term	Definition
Adverse Event (AE)	Any untoward medical occurrence in a participant to whom a medicinal product has been administered, including occurrences which are not necessarily caused by or related to that product.
Adverse Reaction (AR)	An untoward and unintended response in a participant to an investigational medicinal product which is related to any dose administered to that participant.
Serious Adverse Event (SAE)	An adverse event that:<br>• results in death<br>• is life-threatening*<br>• requires inpatient hospitalisation or prolongation of existing hospitalisation**<br>• results in persistent or significant disability/incapacity<br>• consists of a congenital anomaly or birth defect<br>• other medically important condition***<br>*NOTE: The term 'life-threatening' refers to an event in which the participant was at risk of death at the time of the event or it is suspected that used or continued use of the product would result in the subject's death ; it does not refer to an event which hypothetically might have caused death if it were more severe.<br>** Note: Hospitalisation is defined as an inpatient admission, regardless of length of stay, even if the hospitalisation is a precautionary measure for continued observation. Pre-planned hospitalisation, e.g. for pre-existing

For peer review only - http://bmjopen.bmj.com/site/about/guidelines.xhtml

CARDIFF UNIVERSITY PRIFYSGOL CAERDYDD Centre for Trials Research Canolfan Ymchwil Treialon

NHS National Institute for Health Research

itp

University Hospitals Bristol NHS Foundation Trust

	conditions which have not worsened, or elective procedures, does not constitute an SAE.<br>***Note: other events that may not result in death, are not life-threatening, or do not require hospitalisation, may be considered as an SAE when, based upon appropriate medical judgement, the event may jeopardise the participant and may require medical or surgical intervention to prevent one of the outcomes listed above.
Serious Adverse Reaction (SAR)	An SAE occurring in a clinical trial participant for which there is a reasonable possibility that it is **related** to the IMP at any dose administered.
Suspected Unexpected Serious Adverse Reaction (SUSAR)	A SAR, the nature and severity of which is not consistent with the Reference Safety Information (RSI) for the IMP.

10.2 Trial Specific SAE reporting requirements

In addition to the reporting requirements above, for the purposes of this trial the following expected adverse events or reactions listed in the SPC ()

10.3 Causality

Causal relationship will be assessed for IMPs (Mycophenolate, MMF), other trial treatments (non IMPs Steroid) and procedures.

The Principal Investigator (or another delegated medically qualified doctor from the trial team) and Chief Investigator will assess each SAE to determine the casual relationship:

Relationship	Description	Reasonable possibility that the SAE may have been caused by the IMP?
Unrelated	There is no evidence of any causal relationship with the trial/intervention	No
Unlikely	There is little evidence to suggest there is a causal relationship with the trial/intervention (e.g. the event did not occur within a reasonable time after administration of the trial medication). There is another reasonable explanation for the event (e.g. the participant's clinical condition, other concomitant treatment).	No
Possible	There is some evidence to suggest a causal relationship with the trial/intervention (e.g. because the event occurs within a reasonable time after administration of the trial medication). However, the influence of other factors may have contributed to the event (e.g. the participant's clinical condition, other concomitant treatments).	Yes

For peer review only - http://bmjopen.bmj.com/site/about/guidelines.xhtml

Centre for Trials Research
Canolfan Ymchwil Treialon

**Probable**	There is evidence to suggest a causal relationship and the influence of other factors is unlikely.	Yes
**Definite**	There is clear evidence to suggest a causal relationship and other possible contributing factors can be ruled out.	Yes

The causality assessment given by the Principal Investigator (or delegate) cannot be downgraded by the Chief Investigator (or delegate) and in the case of disagreement both opinions will be provided.

10.4: Expectedness

The Chief Investigator (or another delegated appropriate qualified individual) will assess each SAE to perform the assessment of expectedness.

The expectedness assessment should be made with reference to the current Reference Safety Information (RSI) for each IMP. Expectedness decisions must be based purely on the content of the RSI; other factors such as the participant population and participant history should not be taken into account. Expectedness is not related to what is an anticipated event within a particular disease.

SARs which add significant information on specificity or severity of a known, already documented adverse event constitute unexpected events. For example, an event more specific or more severe than that described in the RSI is considered unexpected.

Any SAR that has a fatal or truly serious life threatening outcome will be reported as unexpected and therefore a SUSAR due to the RSI not specifying death and life-threatening events as an expected outcome.

The RSI to be used for the expectedness assessments for this trial are listed in Table 1 below.

Table 1: Reference Safety Information to be used for Expectedness Assessments

IMP	RSI to be used for expectedness assessment	Relevant section of RSI to be used for expectedness assessment
Mycophenolate Mofetil	SPC 250 mg capsules Actavis UK Limited	4.8

The Reference Safety Information (RSI) on any CTR trial will be reviewed regularly according to CTR procedures.

Summary of Product Characteristics (SPC) for Mycophenolate Mofetil 250 mg film-coated Tablets (Appendix 2).

Updates made to the SPC for MMF will be reviewed by the Chief Investigator and Sponsor and a joint decision made whether the updated SPC will be submitted to the MHRA for use as the RSI in the trial.

For peer review only - http://bmjopen.bmj.com/site/about/guidelines.xhtml

10.5 Reporting Procedures

10.5.1 Participating Site Responsibilities

The PI (or delegated medically qualified doctor from the trial team) should sign and date the SAE to acknowledge that he/she has performed the seriousness and causality assessments. Investigators should also report SAEs to their own health boards or trust in accordance with local practice.

A completed SAE form for all events requiring immediate reporting should be submitted via fax or email to the CTR within 24 hours of knowledge of the event. A separate form must be used to report each event, irrespective of whether or not the events had the same date of onset.

The participant will be identified only by trial number, date of birth and initials. The participant's name should not be used on any correspondence.

It is also required that sites respond to and clarify any queries raised on any reported SAEs and report any additional information as and when it becomes available through to the resolution of the event. Additionally, CTR may request additional information relating to any SAE/SARs and the site should provide as much information as is available to them in order to resolve these queries.

Serious Adverse Event (SAE) email address:

CTR-Safety@Cardiff.ac.uk

SAE Fax number:

0203 043 2376

The period of time over which AEs, ARs, SAEs, SARs and SUSARs must be recorded and reported must start from consent:

Where a participant withdraws consent for further processing of data, this does not preclude the reporting of SARs and SUSARs which are required to continue being reported according to the protocol for regulatory purposes.

Adverse events (AE) should be graded using the NCI Common Terminology Criteria for Adverse Events (CTCAE), Version (4.0) (Appendix 5). The toxicity grades should be recorded on the toxicity part of the CRF:

- Full participant trial number
- An Adverse Event / Adverse Reaction
- A completed assessment of the seriousness and causality as performed by PI (or another appropriately medically qualified doctor registered on the delegation log).

For peer review only - http://bmjopen.bmj.com/site/about/guidelines.xhtml

If any of these details are missing, the site will be contacted and the information must be provided by the site to the CTR within 24 hours. Missing data does not preclude CTR's requirement for onward reporting, and all events are treated as worst-case scenario in the case of missing information.

All other AEs should be reported on the CRF following the CRF procedure (section 11)

10.5.2 CTR Responsibilities

Following the initial report, all SAEs should be followed up to resolution wherever possible and further information may be requested by CTR. Follow up information must be provided on a new SAE form.

The CTR should continue reporting SAEs and SARs until 6 weeks after cessation of last dose of MMF. SARs should continue to be reported until the end of follow up.

Once an SAE is received at the CTR, it will be evaluated by staff at the CTR and sent to the Chief Investigator (or their delegate) for an assessment of expectedness.

10.6 SUSAR reporting

University Hospitals Bristol NHS Foundation Trust is undertaking the duties of trial Sponsor and has delegated to Cardiff University CTR the responsibility for reporting SUSARs and other SARs to the regulatory authorities (MHRA and relevant RECs).

Those events that are identified as SUSARs are reported to the MHRA and Research Ethics Committee and as per CTR's local procedures:

SUSARs which are fatal or life-threatening must be reported to the MHRA and REC within 7 calendar days of receipt at the CTR. If report is incomplete then additional follow-up information should be reported within a further 8 calendar days of submitting the initial report.

SUSARs that are not fatal or life-threatening must be reported to the MHRA and REC within 15 days of receipt at the CTR. Any additional, relevant information must be reported within a further 15 days.

N.B. There is no requirement for the CTR to report SUSARs to nIMPs to the MHRA except in the following instances:

- If the adverse reaction is suspected to be linked to an interaction between a nIMP and IMP, and is serious and unexpected, CTR should report as a SUSAR due to the interaction with the IMP.
- If a SUSAR is suspected and might be linked to either a nIMP or an IMP and cannot be attributed to only one of these.
- If the adverse reaction due to the nIMP is likely to affect the safety of trial subjects then CTR should report it to the MHRA and REC in accordance with the relevant Standard Operating Procedure for reporting Urgent Safety Measures.

For peer review only - http://bmjopen.bmj.com/site/about/guidelines.xhtml

10.7 Notification of deaths

All deaths are to be reported as an SAE. Deaths that are related to the IMP are not expected and therefore will always be reported as SUSAR. Only deaths that are assessed to be caused by the IMP will be reported to the sponsor as an SAE. This report will be immediate. Other deaths during treatment or follow up will be captured on the CRF.

10.8 Overdose

Reports of overdoses with Mycophenolate Mofetil have previously been received from clinical trials and during post-marketing experience, in many of these cases, no adverse events were reported. In those overdose cases in which adverse events were reported, the events fall within the known safety profile of the medicinal product.

It is expected that an overdose of Mycophenolate Mofetil could possibly result in oversuppression of the immune system and increase susceptibility to infections and bone marrow suppression. If neutropenia develops, dosing with Mycophenolate Mofetil should be interrupted or the dose reduced.

Details of SAE's associated with overdose (intentional or unintentional) in this trial will be fully described on the SAE report form. An overdose that does not meet the definition of an SAE should be reported via the CRFs.

10.9 Contraception and Pregnancy reporting whilst participating in the trial

As detailed in the exclusion criteria MMF in this trial has a genotoxic and teratogenic potential. Women of childbearing potential must use two reliable forms of contraception simultaneously before starting MMF, during therapy and six weeks after stopping MMF (hormonal or barrier method of birth control; abstinence). Contraceptive methods that can achieve a failure rate of less than 1% per year when used consistently and correctly are considered as highly effective birth control methods. Such methods are included in the Exclusion Criteria (7.2)

Pregnancy, or the pregnancy of a partner occurring whilst participating in the trial, is not considered an SAE, however, a congenital anomaly or birth defect is. Other cases (e.g. termination of pregnancy without information on congenital malformation, and reports of pregnancy exposure without outcome data) should not normally be reported as such. When pregnancy occurs in a trial, either in a female participant or the female partner of a male participant, this should be followed up until at least the end of pregnancy, whether that is a live birth, abortion etc. Without follow-up of the pregnancy, it would not be possible for the CTR to know if a congenital anomaly or birth defect occurred, and therefore if there was an SAE that must be included in the safety evaluation of the IMP. Information on a pregnancy in a trial participant will be captured on the CTR Pregnancy Report Form (this also includes confirmation of participant consent) supplied to sites by the CTR.

Sites should report pregnancy occurring within SAE reporting periods (see Section 9.5). Congenital anomalies or birth defects are considered an SAE and so these events must also be reported to the CTR on a trial-specific SAE form. Congenital anomalies or birth defects related to the IMP and unexpected with respect to the IMP Reference Safety Information (RSI) must be submitted by the CTR within expedited SUSAR time frames (7 or 15 days) to the MHRA, relevant REC.

For peer review only - http://bmjopen.bmj.com/site/about/guidelines.xhtml

10.10 Reporting urgent safety measures

An urgent safety measure is an action that the Sponsor, Chief Investigator or Principal Investigator may carry out in order to protect the subjects of a trial against any immediate hazard to their health or safety. Any urgent safety measure relating to this trial must be notified to the MHRA and Research Ethics Committee immediately by telephone, and in any event within 3 days in writing, that such a measure has been taken. USMs reported to the CTR will be handled according to CTR processes.

11 STATISTICS AND DATA ANALYSIS

11.1 Sample size

There is no published clinical data available for MMF use first line in ITP as this is a novel approach. We have analysed local data on MMF used second line in ITP in 12 patients which shows a median survival free from treatment failure of more than 10 months. We have data on 68 who experienced steroids as a first line treatment showing that 70% of them had experienced a treatment failure by 12 months and that the median survival free from treatment failure was 5.0 months (95% CI [3.2, 6.8]). Data for the 12 patients treated with MMF second line therapy have shorter follow-up times, with only 5 with 12 month data. The cox proportional hazards ratio model demonstrates the 90% confidence interval for the hazard ratio to be between 0.13 and 0.59, showing that our decision to power this on an estimate of a hazard of lower than 0.5 is potentially achievable.

Clinically a doubling in the time to remission was thought to be something that the patients would have welcomed. Less than that was not thought to be sufficient grounds for switching this treatment from second line to first line due to the potential for additional toxicity and immune suppression in those who may have remained in remission with steroids alone.

The sample size of 120 (60 per group) with less than 5% loss to follow-up achieves 91.5% power to detect a doubling of the median time to treatment failure from 5 months to 10 months if the patients are recruited at a steady rate or 10 per month for 12 months and all followed up until the last patients reaches 12 months follow-up.

11.2 Statistical analysis

The full statistical analysis will be written into a statistical analysis plan available separately.

The analysis will produce a CONSORT diagram for the reporting of clinical trials.

The baseline characteristics of the two groups will be tabulated but not tested for statistically significant differences between the groups.

The primary analysis is by intention to treat; however an investigation of compliance with the treatment pathway and compliance with the criteria for changing to a second line therapy will be carried out prior to the primary analysis to check the date of the primary event. The primary event is the date at which there was a requirement for second line therapy. Where the platelet count falls below the level required for this treatment

For peer review only - http://bmjopen.bmj.com/site/about/guidelines.xhtml

decision, the first date at which either symptoms or a blood test revealing this event will be used. If a clinician decides to use a second line therapy without a platelet count below the criteria, the date of the treatment decision/new prescription will be taken to represent that event. The results will be expressed as a hazard ratio with 95% confidence interval, median time to event if more than 50% have had an event and plotted as Kaplen-Mier curves.

The primary analysis will contain all patients who are randomised for as long as they have been followed up or until their first event in a survival analysis using intention to treat methodology. All patients will be followed up to 12 months. In addition patients who have not had an event in the first 12 months post randomisation will be followed until their first event or until the last patient has reached the 12 month point – whichever is the sooner and included in the analysis until that time accordingly.

Analysis of other outcomes will use as full a data set as possible and focus on the 12 month data point or area under the curve as appropriate and detailed in the analysis plan.

11.3 Interim analysis

No interim analyses of the main endpoint will be supplied to the independent Data Monitoring Committee (DMC) due to the short time frame (12 months recruitment) in which all patients will be recruited by the time the first patient has completed follow-up. Adverse event rates will be reported on a monthly basis to the TMG, may be tabulated and provided to the DMC. The DMC could advise the chairman of the Trial Steering Committee and Chief Investigator if these provide proof beyond reasonable doubt that it would be unethical to continue with the trial.

A graph showing expected accrual and actual accrual will be presented to the trial management team, steering committee and DMC at timely intervals.

11.4 Procedure(s) to account for missing or spurious data

For the primary outcome of time to first event – all patients will be included for as long as they were followed up. Patients who deviate from the protocol will be analysed under intention to treat methodology. Patients who choose to completely withdraw from the study will be included from the date of randomisation to the date at which they withdrew. Patients who are lost to follow-up will be included from the date of randomisation to the date at which they were last seen.

11.5 Economic evaluation

An economic evaluation of the intervention will be conducted from an NHS and personal and social services perspective, with results expressed in terms of both cost per relapse averted and per QALY. Health related quality of life will be collected via EQ-5D-5L at baseline, 2, 6 and 12 months.

Detailed resource use will be collected from haematology services (i.e. clinic visits, blood tests, medication use and procedures for treating bleeding events and side effects) and use of all NHS & personal and social services will be monitored via CRF and questionnaires. To estimate costs, resource use will be valued using national sources Curtis 2014, DoH 2014) for a standard price year. Where national unit cost data is not available for

For peer review only - http://bmjopen.bmj.com/site/about/guidelines.xhtml

items of resource use, local data will be identified pertaining to the participating centres. A secondary analysis will consider the societal perspective and therefore patient level indirect costs (i.e. lost productivity, out of pocket expenses) will be collected. Analysis will follow good practice guidelines (NICE 2013, Gold 1996, Glick 2007) and will include the construction of cost-effectiveness acceptability curves to evaluate sampling uncertainty as appropriate.

Both the aforementioned cost-effectiveness analysis and cost-utility analysis will be conducted as a within trial analysis. We note that the trial analysis will potentially be conservative about the benefits accruing to patients beyond the trial period due to the longer term osteoporotic implications associated with repeated steroid use. Therefore, if there is a difference in primary outcome at 1 year, we will use simple decision analytic techniques to provide a cost utility analysis over a longer time horizon.

Patients with ITP are seen in local haematology units at a rate of approximately 12 per year (depending on the size of the unit). Most are diagnosed in office hours and remain outpatients requiring frequent follow up appointments. Up to 30 of the largest will be used as centres, with additional patients referred from nearby interested centres. If a recruitment rate of 50% is achieved, the sample size of 120 patients will be reached within 12 months. Because there is normally only one consultant who sees these patients per site, the trial has excellent support from the patient group and there has been a lot of interest from clinicians in the study, the recruitment rates may be higher than 50%. It is intended that recruitment should aim for 120 patients, expecting to achieve 90 complete sets of data.

12 DATA HANDLING

Source documents produced for this trial will be kept in the patient's hospital records and source data will be transcribed into trial-specific Case Report Forms (CRFs) at the end of each patient visit. It is intended to develop data recording for this trial via a web based system. This is a secure encrypted system accessed by an institutional password which complies with Data Protection Act standards. The database will be stored and regularly backed up on a Cardiff University Server. The CRFs will be coded with the study number and will not include patients' names and addresses

Paper records will be kept in a locked cabinet in secure premises at all times when the record is not in use for a study visit. Access to the records will be restricted to researchers working on the study, Sponsor representatives and representatives of regulatory authorities required to audit the conduct of the research study.

Identifiable data including the link between the patients' names and the study number will be stored separately from other data. All files will be password protected. Electronic data containing personalised information will be saved on Cardiff University computers only in password protected files and backed up regularly to hard copy on secure remote Cardiff University servers. Participant data will be anonymised by the use of study numbers. A copy of the study number code identifying subjects will be kept in a secure cabinet at local study sites accessible to the investigators at all times. Analysis will be conducted by the study team. Analysis will only be conducted on anonymised data.

For peer review only - http://bmjopen.bmj.com/site/about/guidelines.xhtml

The Chief Investigator will act as custodian of the data, however for practical purposes this role will be delegated to CTR, Cardiff University. Personal data will be stored for a minimum of 15 years. Access will be controlled by the Chief Investigator who will continue to act as custodian for all data and will permit trial related monitoring, audits, REC review, and regulatory inspections (where appropriate) by providing direct access to source data and all other documents (i.e. patients' case sheets, blood test reports, etc).

The patient information sheet includes the above information, and the patient must consent to the above data access arrangements in order to enter the trial.

Trial related information in patient medical records will be flagged as 'do not destroy'.

13 QUALITY CONTROL & ASSURANCE

13.1 Monitoring, Audit & Inspections

The clinical trial risk assessment has been used to determine the intensity and focus of central and on-site monitoring activity in this trial. The study will be monitored in accordance with University Hospitals Bristol's Monitoring and Oversight of Research Activity SOP. All trial related documents will be made available on request for monitoring and audit by UH Bristol, the relevant Research Ethics Committee and for inspection by the Medicines and Healthcare products Regulatory Authority or other licensing bodies. The monitoring plan will be developed and agreed by the sponsor.

.

Investigators should agree to allow trial related monitoring, including audits and regulatory inspections, by providing direct access to source data/documents as required. Participant consent for this will be obtained.

Findings generated from on-site and central monitoring will be shared with the Sponsor, CI, PI & local R&D.

13.2 Quality Control

Where applicable, a random sample of at least 10% of CRFs will be checked, by the trial Research Team or UHB Research & Innovation monitor, against entries within the database and with the source data for quality purposes. The percentage checked will be increased if a significant error rate is found. The data from the first patient recruited at a new site will be reviewed. This may include consent records, safety data and primary endpoint data.

14 ETHICAL AND REGULATORY CONSIDERATIONS

This study will be conducted in accordance with:

- The Medicines for Human Use (Clinical Trials) Regulations 2004 and subsequent amendments
- International Conference for Harmonisation of Good Clinical Practice (ICH GCP) guidelines
- Research Governance Framework for Health and Social Care

This protocol and related documents will be submitted for review by a Research Ethics Committee (REC) that is legally 'recognised' by the United Kingdom Ethics Committee Authority for review and approval and to the Medicines and Healthcare products Regulatory Agency (MHRA) for Clinical Trial Authorisation approval.

For peer review only - http://bmjopen.bmj.com/site/about/guidelines.xhtml

This trial protocol will also be submitted through HRA (as it is an English led study) for global governance review. Approval will be obtained from the host care organisation – University Hospitals Bristol NHS Foundation Trust - who will consider local governance requirements and site feasibility. The Research Governance approval of the host care organisation will be obtained before recruitment of participants within that host care organisation.

The Sponsor and CTR will make the decision on non-substantial and substantial amendments for this trial. Valid substantial amendments will be submitted to REC and/or MHRA, dependent on whether the amendment applies to the original CTA and/or REC application, for review. Such amendments may also need to be reviewed and accepted by NHS R&D departments before they can be implemented in practice at sites. All amendment paperwork will be version and date controlled to ensure the amendment history is tracked.

The Chief Investigator (or delegate) will submit annual progress and safety reports and a final report at conclusion of the trial to the REC and the MHRA within the timelines defined in the Regulations.

14.1 Peer review

This trial has been subject to high quality independent and expert peer review. The NIHR funding application was also peer reviewed by the NIHR review committee board and 3 reviewers (independent and expert). It has also been peer reviewed and approved by members of the UK ITP forum (Haematologists specialising in ITP).

14.2 Public and Patient Involvement

Patients have been Involved in identifying the research topic/prioritising the research questions, and preparing the funding application. They have had invaluable input into the design of the research and will continue to contribute to management of the research (patient representation on the steering/advisory group), developing participant information resources, contributing to the reporting of the study report and dissemination of research findings.

Direct feedback from patients regarding the difficulties they face in the first months following ITP diagnosis has been the primary driving force for this research proposal. The current standard of care for first line treatment is high dose steroids, but this has several downsides for patients. Local (Bristol) and national patient groups (ITP support association) have been fundamental to the formulation of patient relevant priorities for treatment. Patients report that avoidance of relapse, early achievement of a stable platelet count, reduced overall steroid dose and reduced hospital attendances are the most important goals for ITP management from their perspective.

In the UK, Mycophenolate (MMF) is a popular second line treatment because it is well tolerated with good efficacy (50-80% response rates). Patients agreed that it was a good idea to try bringing forward the timing of MMF to first line so that it would have "taken over" by the time the first course of steroid had finished, preventing relapse. They supported the proposed trial end points and data collection and encouraged inclusion of patient oriented outcomes (quality of life, fatigue, time off work). They suggested that any extra blood tests needed for the trial and data collection should be done during usual care appointments. These suggestions have

For peer review only

For peer review only - http://bmjopen.bmj.com/site/about/guidelines.xhtml

been incorporated into the design. This research proposal has been discussed and received the support from local and national patient groups.

This clinical trial is supported by the ITP support association which is a patient and public group formed in 1995 by Shirley Watson MBE who is an extremely valuable co-applicant for this clinical trial. As the only UK charity for this disorder, the ITP support Association has been in touch with over 4000 UK ITP sufferers to date, and has members in every continent. It organises annual patient conventions, monthly newsletters and through these and its website, is an important source of support and information for health care professionals, patients and their relatives. These communication links will be utilised to optimise communication to and feedback from the patients and public during and after this trial.

14.3 Protocol compliance

Protocol deviations, non-compliances, or breaches are departures from the approved protocol.

- prospective, planned deviations or waivers to the protocol are not allowed under the UK regulations on Clinical Trials and must not be used e.g. it is not acceptable to enrol a subject if they do not meet the eligibility criteria or restrictions specified in the trial protocol
- accidental protocol deviations can happen at any time. They must be adequately documented on the relevant forms and reported to the Chief Investigator and Sponsor immediately.
- deviations from the protocol which are found to frequently recur are not acceptable, will require immediate action and could potentially be classified as a serious breach.
- Variations in steroid dose and duration are expected and may not exactly follow the treatment flow chart but are NOT considered protocol deviations. The initial daily dose of prednisolone should not exceed 1mg/kg with a maximum of 100mg.

14.4 Data protection and patient confidentiality

The CTR will act to preserve participant confidentiality and will not disclose or reproduce any information by which participants could be identified, except where specific consent is obtained. Data will be stored in a secure manner and will be registered in accordance with the Data Protection Act 1998. The data custodian and the translational sample custodian for this trial is the Chief Investigator.

This includes collection of NHS number (or equivalent – e.g. CHI number in Scotland), name and postcode to register and trace participants with the Health and Social Care Information Centre (HSCIC). Participants will be asked to consent separately for this

14.5 Financial and other competing interests for the chief investigator, PIs at each site and committee members for the overall trial management

As MMF is a generic drug we do not expect any relevant competing interests that might influence trial design, conduct, or reporting.

For peer review only - http://bmjopen.bmj.com/site/about/guidelines.xhtml

14.6 Indemnity

This is an NHS-sponsored study. For NHS sponsored research HSG(96)48 reference No. 2 refers. If there is negligent harm during the clinical trial when the NHS body owes a duty of care to the person harmed, NHS Indemnity covers NHS staff, medical academic staff with honorary contracts and those conducting the trial.

NHS Indemnity does not offer no-fault compensation and is unable to agree in advance to pay compensation for non-negligent harm. Ex-gratia payments may be considered in the case of a claim.

15 TRIAL MANAGEMENT

15.1 TMG (Trial Management Group)

TMG members will be required to sign up to the remit and conditions as set out in the TMG Charter. The TMG will oversee the day to day trial management and will meet in person or by teleconference on a monthly basis for the duration of the study. The TMG will overview and provide guidance on all aspects of regulatory approval, set-up, recruitment, protocol deviations, adverse events, data management, data analysis and dissemination. The TMG will report 6 monthly to the TSC and to the study sponsor as required.

15.2 TSC (Trial Steering Committee)

TSC members will be required to sign up to the remit and conditions as set out in the TSC Charter. The TSC will meet in person or by teleconference at a minimum of 6 monthly intervals during the trial. The TSC principal responsibilities will be to:

- Review the protocol and comment on any major concerns in the trial design that they feel would prevent it addressing the primary objectives.
- Review progress reports on the trial and provide advice if problems arise.
- Comment on protocol amendments.
- Protect the interests of patients should safety issues arise.
- Ensure the integrity of the data as far as they are able.

15.3 DMC (Data Monitoring Committee)

DMC members will be required to sign up to the remit and conditions as set out in the DMC Charter and will review the accumulating safety and efficacy results of the trial on a continuous basis.

16 ANCILLARY TRANSLATIONAL RESEARCH SUB-STUDY

Patients will also be invited to participate in the following optional investigation for ancillary translation research study - (FLIGHT additional laboratory study) which has been funded by a combination of charitable sources:

Patients will be asked to consent to provide additional blood samples at V1 and V2.

For peer review only - http://bmjopen.bmj.com/site/about/guidelines.xhtml

The blood samples will be sent to the Bristol Biobank in the Bristol Royal Infirmary. The samples will be labelled with a unique identifier so that they can be linked to the blood results already collected by the FLIGHT study and compare laboratory results with patient outcomes. Laboratory staff will not be able to directly link the samples to patients. The samples will be processed by University of Bristol immediately after collection. Any remaining samples will be stored securely in the Bristol Biobank for an indefinite amount of time for future research within the UK. Patients will be asked to consent separately for this.

The objectives of this laboratory sub-study include the development of laboratory biomarkers to improve the prediction of clinical outcomes in ITP including illness severity, chronicity and response to treatment. The long term goal of the translational research is to individualise treatment strategy for patients with ITP, enabling earlier disease control and avoiding side effects of ineffective or unnecessary treatments.

17 DISSEMINATION POLICY

The trial will be registered on ISRCTN and Clinicaltrials.gov websites.

The data arising from the trial is owned by Sponsor and managed in accordance with the Sponsor's Collaboration Agreement.

On completion of the trial, the data will be analysed and tabulated and a Final Study Report prepared which can be accessed by EudraCT Clinical Trials Database. The responsibility for developing and disseminating the final trial report will reside with the trial team (CI, statistician, trial manager, Sponsor, etc.)

The trial findings will be presented at national and international meetings and published in high quality peer review journals with acknowledgement of NIHR funding and participating centres. In addition, a lay summary for patients and public will be generated and published in the magazine ("the platelet") published by the ITP Support Association.

18 ARCHIVING

The TMF and TSF containing essential documents will be archived at an approved external storage facility for a minimum of 15 years. The CTR will maintain the TMF and TSFs and prepare them for archiving on behalf of the Sponsor. The Principal Investigator is responsible for archival of the ISF at site as documented in the Site Agreement. Essential documents pertaining to the trial shall not be destroyed without permission from the Sponsor.

For peer review only - http://bmjopen.bmj.com/site/about/guidelines.xhtml

19 REFERENCES

1. Moulis G, Palmaro A, Montastruc JL, Godeau B, Lapeyre-Mestre M, Sailler L. Epidemiology of incident immune thrombocytopenia: a nationwide population-based study in France. Blood 2014;124:3308-15.

2. McKenzie CG, Guo L, Freedman J, Semple JW. Cellular immune dysfunction in immune thrombocytopenia (ITP). British journal of haematology 2013;163:10-23.

3. Iraqi M, Perdomo J, Yan F, Choi PY, Chong BH. Immune thrombocytopenia: antiplatelet autoantibodies inhibit proplatelet formation by megakaryocytes and impair platelet production in vitro. Haematologica 2015;100:623-32.

4. Ji X, Zhang L, Peng J, Hou M. T cell immune abnormalities in immune thrombocytopenia. Journal of hematology & oncology 2014;7:72.

5. Audia S, Rossato M, Santegoets K, et al. Splenic TFH expansion participates in B-cell differentiation and antiplatelet-antibody production during immune thrombocytopenia. Blood 2014;124:2858-66.

6. Hu Y, Wang X, Yu S, Hou Y, Ma D, Hou M. Neutralizations of IL-17A and IL-21 regulate regulatory T cell/Thelper 17 imbalance via T-helper 17-associated signaling pathway in immune thrombocytopenia. Expert opinion on therapeutic targets 2015;19:723-32.

7. Yu S, Liu C, Li L, et al. Inactivation of Notch signaling reverses the Th17/Treg imbalance in cells from patients with immune thrombocytopenia. Laboratory investigation; a journal of technical methods and pathology 2015;95:157-67.

8. Kuwana M, Okazaki Y, Ikeda Y. Detection of circulating B cells producing anti-GPIb autoantibodies in patients with immune thrombocytopenia. PloS one 2014;9:e86943.

9. Li F, Ji L, Wang W, et al. Insufficient secretion of IL-10 by Tregs compromised its control on over-activated CD4+ T effector cells in newly diagnosed adult immune thrombocytopenia patients. Immunologic research 2015;61:269-80.

10. Hua F, Ji L, Zhan Y, et al. Aberrant frequency of IL-10-producing B cells and its association with Treg/Th17 in adult primary immune thrombocytopenia patients. BioMed research international 2014;2014:571302.

11. Ma L, Liang Y, Fang M, et al. The cytokines (IFN-gamma, IL-2, IL-4, IL-10, IL-17) and Treg cytokine (TGFbeta1) levels in adults with immune thrombocytopenia. Die Pharmazie 2014;69:694-7.

12. Cines DB, Cuker A, Semple JW. Pathogenesis of immune thrombocytopenia. Presse medicale (Paris, France : 1983) 2014;43:e49-59.

13. Nishimoto T, Kuwana M. CD4+CD25+Foxp3+ regulatory T cells in the pathophysiology of immune thrombocytopenia. Seminars in hematology 2013;50 Suppl 1:S43-9.

14. Provan D, Stasi R, Newland AC, et al. International consensus report on the investigation and

For peer review only - http://bmjopen.bmj.com/site/about/guidelines.xhtml

management of primary immune thrombocytopenia. Blood 2010;115:168-86.
15. Neunert C, Lim W, Crowther M, Cohen A, Solberg L, Jr., Crowther MA. The American Society of Hematology 2011 evidence-based practice guideline for immune thrombocytopenia. Blood 2011;117:4190-207.
16. Brown TM, Horblyuk RV, Grotzinger KM, Matzdorff AC, Pashos CL. Patient-reported treatment burden of chronic immune thrombocytopenia therapies. BMC blood disorders 2012;12:2.
17. Neunert C, Noroozi N, Norman G, et al. Severe bleeding events in adults and children with primary immune thrombocytopenia: a systematic review. Journal of thrombosis and haemostasis : JTH 2015;13:457-64.
18. Hill QA, Newland AC. Fatigue in immune thrombocytopenia. British journal of haematology 2015;170:141-9.
19. Snyder CF, Mathias SD, Cella D, Isitt JJ, Wu AW, Young J. Health-related quality of life of immune thrombocytopenic purpura patients: results from a web-based survey. Current medical research and opinion 2008;24:2767-76.
20. Tarantino MD, Mathias SD, Snyder CF, Isitt JJ, Gernsheimer T, Young J. Impact of ITP on physician visits and workplace productivity. Current medical research and opinion 2010;26:319-28.
21. Taylor A, Neave L, Solanki S, et al. Mycophenolate mofetil therapy for severe immune thrombocytopenia. British journal of haematology 2015;171:625-30.
22. Kotb R, Pinganaud C, Trichet C, et al. Efficacy of mycophenolate mofetil in adult refractory auto-immune cytopenias: a single center preliminary study. European journal of haematology 2005;75:60-4.
23. Provan D, Moss AJ, Newland AC, Bussel JB. Efficacy of mycophenolate mofetil as single-agent therapy for refractory immune thrombocytopenic purpura. American journal of hematology 2006;81:19-25.
24. Hou M, Peng J, Shi Y, et al. Mycophenolate mofetil (MMF) for the treatment of steroid-resistant idiopathic thrombocytopenic purpura. European journal of haematology 2003;70:353-7.
25. Colovic M, Suvajdzic N, Colovic N, Tomin D, Vidovic A, Palibrk V. Mycophenolate mophetil therapy for chronic immune thrombocytopenic purpura resistant to steroids, immunosuppressants, and/or splenectomy in adults. Platelets 2011;22:153-6.
26. Zhang WG, Ji L, Cao XM, et al. Mycophenolate mofetil as a treatment for refractory idiopathic thrombocytopenic purpura. Acta pharmacologica Sinica 2005;26:598-602.
27. Howard J, Hoffbrand AV, Prentice HG, Mehta A. Mycophenolate mofetil for the treatment of refractory auto-immune haemolytic anaemia and auto-immune thrombocytopenia purpura. British journal of haematology 2002;117:712-5.

For peer review only - http://bmjopen.bmj.com/site/about/guidelines.xhtml

28. Kristinsson SY, Gridley G, Hoover RN, Check D, Landgren O. Long-term risks after splenectomy among 8,149 cancer-free American veterans: a cohort study with up to 27 years follow-up. Haematologica 2014;99:392-8.

29. Ghanima W, Khelif A, Waage A, et al. Rituximab as second-line treatment for adult immune thrombocytopenia (the RITP trial): a multicentre, randomised, double-blind, placebo-controlled trial. Lancet (London, England) 2015;385:1653-61.

30. Gudbrandsdottir S, Birgens HS, Frederiksen H, et al. Rituximab and dexamethasone vs dexamethasone monotherapy in newly diagnosed patients with primary immune thrombocytopenia. Blood 2013;121:1976-81.

31. Psaila B, Cooper N. B-cell depletion in immune thrombocytopenia. Lancet (London, England) 2015;385:1599-601.

32. Rodeghiero F, Ruggeri M. Treatment of immune thrombocytopenia in adults: the role of thrombopoietinreceptor agonists. Seminars in hematology 2015;52:16-24.

33. Gomez-Almaguer D, Herrera-Rojas MA, Jaime-Perez JC, et al. Eltrombopag and high-dose dexamethasone as frontline treatment of newly diagnosed immune thrombocytopenia in adults. Blood 2014;123:3906-8.

34. Rodeghiero, F., et al., Standardization of terminology, definitions and outcome criteria in immune thrombocytopenic purpura of adults and children: report from an international working group. Blood, 2009;113(11): p. 2386-93.

35. Department of Health. National schedule of reference costs for NHS trusts and NHS foundation trusts 2013- 2014. Available at: https://www.gov.uk/government/publications/nhs-reference-costs-2013-to-2014. November 2014.

36. Curtis L. Unit Costs of Health and Social Care, PSSRU 2014. Available from: http://www.pssru.ac.uk/project-pages/unit-costs/2014/.

37. NICE. Guide to the Methods of Technology appraisal. 2013.

38. Gold MR. Cost-effectiveness in health and medicine. New York: Oxford University Press; 1996. xxiii, 425p. p.

39. Glick H. Economic evaluation in clinical trials. Oxford ; New York: Oxford University Press; 2007. x, 244 p. p.

40. Miano M, Ramenghi U, Russo G, Rubert L, Barone A, Tucci F, Farruggia P, Petrone A, Mondino A, Lo Valvo L, Crescenzio N, Bellia F, Olivieri I, Palmisani E, Caviglia I, Dufour C, Fioredda F. Mycophenolate mofetil for the treatment of children with immune thrombocytopenia and Evans syndrome. A retrospective data review from the Italian association of paediatric haematology/oncology. Br J Haematol. 2016 Jul 22.

For peer review only

For peer review only - http://bmjopen.bmj.com/site/about/guidelines.xhtml

20 APPENDICIES

APPENDIX 1 – SCHEDULE OF PROCEDURES

Procedures	V0	V1	V2 (2 mths)	V3 (4 mths)	V4 (6 mths)	V5 (12 mths)	V6 12-24 mths
	Screen	Baseline / Randomisation to pathway 1 or 2	Follow up	Follow up	Follow up	Follow up	Data collection from sites
Eligibility assessment	x						
Randomisation		x					
Informed consent		x					
Demographics		x					
Medical history		x	x	x	x	x	
Physical examination		x					
Vital signs (incl height & weight)		x	x	x	x	x	
Pregnancy test	x						
Concomitant medications		x	x	x	x	x	
Standard practice bloods (includes blood sugar if applicable)	x	x	x	x	x	x	
Hepatitis B, C & HIV serology	x						
Immunoglobulins (blood)		x			x	x	
Extra blood samples (optional)		x	x				
Dispensing of trial drugs		x*					
Compliance			x				
QofL FACT-Th6, V4	x	x	x	x	x	x	
QofL ICECAP V2 – A measure	x	x	x	x	x	x	
QofL SF-36v2 – Health Survey	x	x	x	x	x	x	
QofL FACIT-F, V4, pg 3 (fatigue)	x	x	x	x	x	x	
QofL Thrombocytopenia costs questionnaire	x	x	x	x	x	x	
Data collection from sites on platelet count & treatment		x	x	x	x	x	x

* MMF and corticosteroid dispensing frequency can follow standard local practice.

For peer review only - http://bmjopen.bmj.com/site/about/guidelines.xhtml

APPENDIX 2 - MYCOPHENOLATE MOFETIL

A full and up to date description is available via the link below:

https://www.medicines.org.uk/emc/medicine/24288

APPENDIX 3 – PREDNISOLONE

A full and up to date description is available via the link below:

https://www.medicines.org.uk/emc/medicine/24130

APPENDIX 4 – DEXAMETHASONE

A full and up to date description is available via the link below:

http://www.medicines.org.uk/emc/medicine/28919

APPENDIX 5 – COMMON TERMINOLOGY CRITERIA FOR GRADING OF ADVERSE EVENTS

Grading of toxicity and adverse events will be made according to the Common Terminology Criteria for Adverse Events (CTCAE) version 4. The full CTCAE document is available in the Investigator Site File and on the National Cancer Institute (NCI) website. The following address was correct when this version of the protocol was approved:

http://ctep.cancer.gov/protocolDevelopment/electronic_applications/ctc.htm

For peer review only - http://bmjopen.bmj.com/site/about/guidelines.xhtml

APPENDIX 6 – QUALITY OF LIFE QUESTIONNAIRES

FACT-Th6 (Version 4)

English (Universal) 20 September 2016 Copyright 1987, 1997 Page 1 of 1

Below is a list of statements that other people with your illness have said are important.

Please circle or mark one number per line to indicate your response as it applies to the <u>past 7 days.</u>

		Not at all	A little bit	Some-what	Quite a bit	Very much
An7	I am able to do my usual activities	0	1	2	3	4
Th3	I worry about problems with bruising or bleeding ……	0	1	2	3	4
Th4	I worry about the possibility of serious bleeding	0	1	2	3	4
Th10	I avoid or limit physical activity (because of concern with bleeding or bruising)	0	1	2	3	4
Th11	I avoid or limit social activity (because of concern with bleeding or bruising)	0	1	2	3	4
Th12	I am frustrated by not being able to do my usual activities	0	1	2	3	4

For peer review only - http://bmjopen.bmj.com/site/about/guidelines.xhtml

© 2010 Hareth Al-Janabi and Joanna Coast

ABOUT YOUR OVERALL QUALITY OF LIFE

Please indicate which statements best describe your overall quality of life at the moment by placing a tick (ü) in **ONE** box for each of the five groups below.

1. Feeling settled and secure

I am able to feel settled and secure in **all** areas of my life	4	☐
I am able to feel settled and secure in **many** areas of my life	3	☐
I am able to feel settled and secure in **a few** areas of my life	2	☐
I am **unable** to feel settled and secure in **any** areas of my life	1	☐

2. Love, friendship and support

I can have **a lot** of love, friendship and support	4	☐
I can have **quite a lot** of love, friendship and support	3	☐
I can have **a little** love, friendship and support	2	☐
I **cannot** have **any** love, friendship and support	1	☐

3. Being independent

I am able to be **completely** independent	4	☐
I am able to be independent in **many** things	3	☐
I am able to be independent in **a few** things	2	☐
I am **unable** to be at all independent	1	☐

For peer review only

For peer review only - http://bmjopen.bmj.com/site/about/guidelines.xhtml

4. Achievement and progress

I can achieve and progress in **all** aspects of my life [4]	☐
I can achieve and progress in **many** aspects of my life [3]	☐
I can achieve and progress in **a few** aspects of my life [2]	☐
I **cannot** achieve and progress in **any** aspects of my life [1]	☐

5. Enjoyment and pleasure

I can have **a lot** of enjoyment and pleasure [4]	☐
I can have **quite a lot** of enjoyment and pleasure [3]	☐
I can have **a little** enjoyment and pleasure [2]	☐
I **cannot** have **any** enjoyment and pleasure [1]	☐

Please ensure you have only ticked **ONE** box for each of the five groups.

For peer review only

For peer review only - http://bmjopen.bmj.com/site/about/guidelines.xhtml

Your Health and Well-Being

This survey asks for your views about your health. This information will help keep track of how you feel and how well you are able to do your usual activities. ***Thank you for completing this survey!***

For each of the following questions, please tick the one box that best describes your answer.

1. In general, would you say your health is:

Excellent	Very good	Good	Fair	Poor
☐ 1	☐ 2	☐ 3	☐ 4	☐ 5

2. Compared to one year ago, how would you rate your health in general now?

Much better now than one year ago	Somewhat better now than one year ago	About the same as one year ago	Somewhat worse now than one year ago	Much worse now than one year ago
☐ 1	☐ 2	☐ 3	☐ 4	☐ 5

For peer review only - http://bmjopen.bmj.com/site/about/guidelines.xhtml

3. The following questions are about activities you might do during a typical day. Does <u>your health now limit you</u> in these activities? If so, how much?

	Yes, limited a lot	Yes, limited a little	No, not limited at all
a Vigorous activities, such as running, lifting heavy objects, participating in strenuous sports	☐ 1	☐ 2	☐ 3
b Moderate activities, such as moving a table, pushing a vacuum cleaner, bowling, or playing golf	☐ 1	☐ 2	☐ 3
c Lifting or carrying groceries	☐ 1	☐ 2	☐ 3
d Climbing several flights of stairs	☐ 1	☐ 2	☐ 3
e Climbing one flight of stairs	☐ 1	☐ 2	☐ 3
f Bending, kneeling, or stooping	☐ 1	☐ 2	☐ 3
g Walking more than a mile	☐ 1	☐ 2	☐ 3
h Walking several hundred yards	☐ 1	☐ 2	☐ 3
i Walking one hundred yards	☐ 1	☐ 2	☐ 3
j Bathing or dressing yourself	☐ 1	☐ 2	☐ 3

For peer review only

For peer review only - http://bmjopen.bmj.com/site/about/guidelines.xhtml

4. During the <u>past 4 weeks</u>, how much of the time have you had any of the following problems with your work or other regular daily activities <u>as a result of your physical health</u>?

	All of the time	Most of the time	Some of the time	A little of the time	None of the time
a Cut down on the amount of time you spent on work or other activities	☐ 1	☐ 2	☐ 3	☐ 4	☐ 5
b Accomplished less than you would like	☐ 1	☐ 2	☐ 3	☐ 4	☐ 5
c Were limited in the kind of work or other activities	☐ 1	☐ 2	☐ 3	☐ 4	☐ 5
d Had difficulty performing the work or other activities (for example, it took extra effort)	☐ 1	☐ 2	☐ 3	☐ 4	☐ 5

5. During the <u>past 4 weeks</u>, how much of the time have you had any of the following problems with your work or other regular daily activities <u>as a result of any emotional problems</u> (such as feeling depressed or anxious)?

	All of the time	Most of the time	Some of the time	A little of the time	None of the time
a Cut down on the amount of time you spent on work or other activities	☐ 1	☐ 2	☐ 3	☐ 4	☐ 5
b Accomplished less than you would like	☐ 1	☐ 2	☐ 3	☐ 4	☐ 5
c Did work or other activities less carefully than usual	☐ 1	☐ 2	☐ 3	☐ 4	☐ 5

For peer review only - http://bmjopen.bmj.com/site/about/guidelines.xhtml

6. **During the past 4 weeks, to what extent has your physical health or emotional problems interfered with your normal social activities with family, friends, neighbours, or groups?**

Not at all	Slightly	Moderately	Quite a bit	Extremely
☐ 1	☐ 2	☐ 3	☐ 4	☐ 5

7. **How much bodily pain have you had during the past 4 weeks?**

None	Very mild	Mild	Moderate	Severe	Very severe
☐ 1	☐ 2	☐ 3	☐ 4	☐ 5	☐ 6

8. **During the past 4 weeks, how much did pain interfere with your normal work (including both work outside the home and housework)?**

Not at all	A little bit	Moderately	Quite a bit	Extremely
☐ 1	☐ 2	☐ 3	☐ 4	☐ 5

For peer review only - http://bmjopen.bmj.com/site/about/guidelines.xhtml

9. These questions are about how you feel and how things have been with you <u>during the past 4 weeks</u>. For each question, please give the one answer that comes closest to the way you have been feeling. How much of the time during the <u>past 4 weeks</u>…

	All of the time	Most of the time	Some of the time	A little of the time	None of the time
a Did you feel full of life?	☐ 1	☐ 2	☐ 3	☐ 4	☐ 5
b Have you been very nervous?	☐ 1	☐ 2	☐ 3	☐ 4	☐ 5
c Have you felt so down in the dumps that nothing could cheer you up?	☐ 1	☐ 2	☐ 3	☐ 4	☐ 5
d Have you felt calm and peaceful?	☐ 1	☐ 2	☐ 3	☐ 4	☐ 5
e Did you have a lot of energy?	☐ 1	☐ 2	☐ 3	☐ 4	☐ 5
f Have you felt downhearted and low?	☐ 1	☐ 2	☐ 3	☐ 4	☐ 5
g Did you feel worn out?	☐ 1	☐ 2	☐ 3	☐ 4	☐ 5
h Have you been happy?	☐ 1	☐ 2	☐ 3	☐ 4	☐ 5
i Did you feel tired?	☐ 1	☐ 2	☐ 3	☐ 4	☐ 5

10. During the <u>past 4 weeks</u>, how much of the time has your <u>physical health or emotional problems</u> interfered with your social activities (like visiting with friends, relatives, etc.)?

All of the time	Most of the time	Some of the time	A little of the time	None of the time
☐ 1	☐ 2	☐ 3	☐ 4	☐ 5

For peer review only - http://bmjopen.bmj.com/site/about/guidelines.xhtml

11. How TRUE or FALSE is <u>each</u> of the following statements for you?

	Definitely true	Mostly true	Don't know	Mostly false	Definitely false
a I seem to get ill more easily than other people	☐ 1	☐ 2	☐ 3	☐ 4	☐ 5
b I am as healthy as anybody I know	☐ 1	☐ 2	☐ 3	☐ 4	☐ 5
c I expect my health to get worse	☐ 1	☐ 2	☐ 3	☐ 4	☐ 5
d My health is excellent	☐ 1	☐ 2	☐ 3	☐ 4	☐ 5

Thank you for completing these questions!

SF-36v2® Health Survey © 1992, 2002, 2009 Medical Outcomes Trust and QualityMetric Incorporated. All rights reserved.

SF-36® is a registered trademark of Medical Outcomes Trust.

(SF-36v2® Health Survey Standard, United Kingdom (English))

For peer review only - http://bmjopen.bmj.com/site/about/guidelines.xhtml

FACIT-F (Version 4)
Please note: Page 3 (Fatigue) of the following Quality of Life questionnaire being used only

FACIT-F (Version 4)

Below is a list of statements that other people with your illness have said are important. **Please circle or mark one number per line to indicate your response as it applies to the <u>past 7 days</u>.**

	**PHYSICAL WELL-BEING**	Not at all	A little bit	Some-what	Quite a bit	Very much
GP1	I have a lack of energy	0	1	2	3	4
GP2	I have nausea	0	1	2	3	4
GP3	Because of my physical condition, I have trouble meeting the needs of my family	0	1	2	3	4
GP4	I have pain	0	1	2	3	4
GP5	I am bothered by side effects of treatment	0	1	2	3	4
GP6	I feel ill	0	1	2	3	4
GP7	I am forced to spend time in bed	0	1	2	3	4

	**SOCIAL/FAMILY WELL-BEING**	Not at all	A little bit	Some-what	Quite a bit	Very much
GS1	I feel close to my friends	0	1	2	3	4
GS2	I get emotional support from my family	0	1	2	3	4
GS3	I get support from my friends	0	1	2	3	4
GS4	My family has accepted my illness	0	1	2	3	4
GS5	I am satisfied with family communication about my illness	0	1	2	3	4
GS6	I feel close to my partner (or the person who is my main support)	0	1	2	3	4
Q1	*Regardless of your current level of sexual activity, please answer the following question. If you prefer not to answer it, please mark this box ☐ and go to the next section.*					
GS7	I am satisfied with my sex life	0	1	2	3	4

English (Universal)
Copyright 1987, 1997

16 November 2007

For peer review only - http://bmjopen.bmj.com/site/about/guidelines.xhtml

FACIT-F (Version 4)

Please circle or mark one number per line to indicate your response as it applies to the past 7 days.

	EMOTIONAL WELL-BEING	Not at all	A little bit	Some-what	Quite a bit	Very much
GE1	I feel sad	0	1	2	3	4
GE2	I am satisfied with how I am coping with my illness	0	1	2	3	4
GE3	I am losing hope in the fight against my illness	0	1	2	3	4
GE4	I feel nervous	0	1	2	3	4
GE5	I worry about dying	0	1	2	3	4
GE6	I worry that my condition will get worse	0	1	2	3	4

	FUNCTIONAL WELL-BEING	Not at all	A little bit	Some-what	Quite a bit	Very much
GF1	I am able to work (include work at home)	0	1	2	3	4
GF2	My work (include work at home) is fulfilling	0	1	2	3	4
GF3	I am able to enjoy life	0	1	2	3	4
GF4	I have accepted my illness	0	1	2	3	4
GF5	I am sleeping well	0	1	2	3	4
GF6	I am enjoying the things I usually do for fun	0	1	2	3	4
GF7	I am content with the quality of my life right now	0	1	2	3	4

English (Universal)
Copyright 1987, 1997

For peer review only - http://bmjopen.bmj.com/site/about/guidelines.xhtml

FACIT-F (Version 4)

Please circle or mark one number per line to indicate your response as it applies to the past 7 days.

	ADDITIONAL CONCERNS	Not at all	A little bit	Some-what	Quite a bit	Very much
HI7	I feel fatigued	0	1	2	3	4
HI12	I feel weak all over	0	1	2	3	4
An1	I feel listless ("washed out")	0	1	2	3	4
An2	I feel tired	0	1	2	3	4
An3	I have trouble starting things because I am tired	0	1	2	3	4
An4	I have trouble finishing things because I am tired	0	1	2	3	4
An5	I have energy	0	1	2	3	4
An7	I am able to do my usual activities	0	1	2	3	4
An8	I need to sleep during the day	0	1	2	3	4
An12	I am too tired to eat	0	1	2	3	4
An14	I need help doing my usual activities	0	1	2	3	4
An15	I am frustrated by being too tired to do the things I want to do	0	1	2	3	4
An16	I have to limit my social activity because I am tired	0	1	2	3	4

English (Universal) 16 November 2007
Copyright 1987, 1997 Page 3 of 3

For peer review only - http://bmjopen.bmj.com/site/about/guidelines.xhtml

FACT-Th6 (Version 4)

Below is a list of statements that other people with your illness have said are important. Please circle or mark one number per line to indicate your response as it applies to the past 7 days.

		Not at all	A little bit	Some-what	Quite a bit	Very much
An7	I am able to do my usual activities	0	1	2	3	4
Th5	I worry about problems with bruising or bleeding	0	1	2	3	4
Th4	I worry about the possibility of serious bleeding	0	1	2	3	4
Th10	I avoid or limit physical activity (because of concern with bleeding or bruising)	0	1	2	3	4
Th11	I avoid or limit social activity (because of concern with bleeding or bruising)	0	1	2	3	4
Th12	I am frustrated by not being able to do my usual activities	0	1	2	3	4

English (Universal)
Copyright 1987, 1997

20 September 2016
Page 1 of 1

For peer review only - http://bmjopen.bmj.com/site/about/guidelines.xhtml

Thrombocytopenia care costs questionnaire, V1.0, May 2017

Please tick one box for the category that describes your employment status prior to your diagnosis of thrombocytopenia.

In full time employment (30 hours or more a week) ☐
In part time employment (less than 30 hours a week) ☐
Employed but on sick leave ☐
Retired ☐
Not in paid employment ☐

If you normally work a fixed number of hours per week, please specify ☐ hours

Do you have dependent children at home? Yes ☐ No ☐

Do you provide support for someone else with health problems? Yes ☐ No ☐

For peer review only

For peer review only - http://bmjopen.bmj.com/site/about/guidelines.xhtml

This set of questions is aimed at finding out the financial cost to you and your family and the health services over the last 2 months. Please think back over the past 2 months. If you are unsure about any answer please write in your best guess.

Please tick one box for the category that describes your current employment

In full time employment (30 hours or more a week)	☐
In part time employment (less than 30 hours a week)	☐
Employed but on sick leave	☐
Retired	☐
Not in paid employment	☐

If you are employed, approximately how much time have you taken off work **in total** during the last 3 months due to your health including to attend blood tests or appointments? ☐ days ☐ hours

Have you **provided** support for someone else with health problems? Yes ☐ No ☐

Have you **received** help or support from family or friends due to your own health problems (including help to attend appointments)? Yes ☐ No ☐

If yes, approximately how much time have they set aside to help you in a **typical week**? ☐ hours ☐ days

If yes, and if they work, approximately how much time have they taken off work in order to help you in a **typical week**? ☐ hours ☐ days

For peer review only - http://bmjopen.bmj.com/site/about/guidelines.xhtml

Healthcare log

In the past 2 months, did you see any health or care professionals in the community? (this refers to all health care and social care that is not based in the hospital. This includes for example your GP, practice or community nurse, social worker, home help or physiotherapist who is not based in the hospital.

Please indicate the person you saw, how many times you saw them. If the type of professional is not listed then please write this in. Please record all contact whether or not this was related to your condition.

Number:	Home visit	Clinic/ practice	By phone
Contact with doctor (GP)			
Contact with nurse			
Other Health Professional (………………..)			
Other Health Professional (………………..)			

For peer review only

Travel

Over the past 2 months:

How many miles in total have you travelled to attend hospital or other health or social care appointments including any unplanned visits? Please record all travel whether or not it was related to your thrombocytopenia.

[ ] miles

How much have your spent on healthcare-related parking?

£

For peer review only - http://bmjopen.bmj.com/site/about/guidelines.xhtml

Prescriptions

Do you pay for your prescriptions? *(please tick)*

Yes	
No	

Other Expenses

Please record any other expenses you have had to meet over the last 2 months because of your health or treatment. Please record all expenses, whether or not they were related to your thrombocytopenia.

Brief description	Cost

For peer review only

For peer review only - http://bmjopen.bmj.com/site/about/guidelines.xhtml

Cardiff University / Prifysgol Caerdydd — Centre for Trials Research / Canolfan Ymchwil Treialon

University Hospitals Bristol NHS Foundation Trust

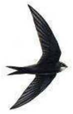

FLIGHT: A multicentre randomised trial of First Line treatment pathways for newly diagnosed Immune Thrombocytopenia: Standard steroid treatment versus combined steroid and mycophenolate.

Patient Information Sheet

Chief Investigator
Dr Charlotte Bradbury, University of Bristol

You are invited to take part in a research trial. Before you decide whether to take part or not, you need to understand why the research is being done and what it would involve for you. Taking part in research is voluntary. You have the right not to join the trial, or to join and later decide to withdraw from the trial without giving any reason for your decision. This will not affect your care.

Please take time to read the following information carefully. **A member of our team will go through the information leaflet with you, explain the trial in more detail, and answer any questions you have**. If anything is not clear or you would like more information, do not hesitate to ask a member of the research team. Talk to friends or relatives about the trial if you wish, and take time to decide. If you would like to take part, you will be asked to confirm by signing a separate consent form. You will also be given a copy for your own records.

What is the purpose of the trial?

The initial treatment for ITP is high dose steroids and has been the same for many years. For most patients these are effective at raising the platelet count and they work quite quickly (usually within 2 weeks). However, the illness usually comes back when the steroids are reduced or stopped leaving patients back at square one. The next treatment used is often Mycophenolate (MMF), which works well but takes time to work.

We plan to try using MMF with steroids from diagnosis to see how well this controls the ITP. To do this we will compare the current steroid only pathway with the new one (steroid with MMF). Patients from different hospitals will be asked to take part and half will be randomly chosen for the new pathway.

Why have I been invited?

We are looking for people like you, who have ITP and need a first course of treatment. We will ask about 120 people in the UK to take part in this trial.

For peer review only - http://bmjopen.bmj.com/site/about/guidelines.xhtml

For peer review only

What will happen to me if I take part?

If you decide to take part you will be treated either with the normal first treatment for ITP= **Steroid only group** or the normal first treatment with MMF= **Steroids and MMF group.**

As part of your standard treatment for ITP, you will have regular blood tests and doctor or nurse reviews to check how you are getting on. This will be the same whether you chose to take part in this trial or not. There will be no extra doctor visits for the research trial and your usual visits will be used to collect information on how you are. It may mean that some of the visits take a bit longer as we will be gathering a bit more information than we normally would.

What advantages and disadvantages are there if I agree to do this trial?

For this trial, if you agree to take part, there will be no additional appointments for blood tests or doctor visits and your usual follow up visits will be used to record how you are. However, it is possible that some of these appointments will take a bit longer than they would do if you were not in the trial so that we can gather all the information needed to carefully record how you are. We will also ask you to fill in a questionnaire at the beginning, at 2 months, 4 months, 6 months and 12 months. This will help us assess how your illness and its treatment are affecting you.

If you are allocated to the steroid only group, you will be treated with the same medicines that you would do if you did not agree to take part. If you are allocated to the new steroids and MMF pathway, you will have the same medicines but will take an extra medicine called MMF. The advantage of receiving MMF first is that your ITP is more likely to be controlled sooner and less likely to come back. Overall, you are expected to need fewer steroids with the associated side effects. The disadvantage of receiving MMF first is that it is possible you may have been one of the few patients who only need steroids to get better (1 in 5) and therefore you will have taken MMF without needing it. In addition, you may be at slightly higher risk of infection and it will be very important that you or your partner do not become pregnant while taking MMF as MMF can cause harm to unborn babies. Importantly, the design of this trial includes an MMF dose reduction and stopping at 6 months if your ITP is well controlled to avoid unnecessary long term use.

Is there anything important I should know before agreeing to the trial?

You must be willing to follow the research nurse/doctor's instructions. You must take the trial medication as instructed. If you miss any doses, you should continue to the next scheduled dose. At each visit you must return any leftover trial medication that you do not take and the container, even if empty.

- It is very important you or your partner do not become pregnant whilst taking MMF or breast feed. Please see contraception advice on page 7 under **Further information to know about taking Mycophenolate.**
- At the beginning of the trial, at 2 months, 4 months, 6 months and 12 months we will ask you some questions regarding your health.
- A card with information about the trial and the research team's contact details will be given to you and you should carry it with you and show it to any other doctors or nurses that you might see.
- You cannot take part in any other research trial that involves taking medication while you are in this trial.

For peer review only - http://bmjopen.bmj.com/site/about/guidelines.xhtml

Centre for Trials Research
Canolfan Ymchwil Treialon
University Hospitals Bristol NHS Foundation Trust

What will happen to any samples I give?

Treatment for ITP includes regular blood tests to check how the illness is responding and these will be processed at your local hospital as they would if you were not in the trial. We will also ask your permission to take some additional blood samples for ITP research at the beginning and after 2 months of treatment. This is optional and you can still take part in the Flight trial but decline to have these blood samples taken, but please consider how valuable these are to our research before making such a decision. You will receive a separate information sheet and consent form for this. If you agree, these blood samples will be taken when you are having routine bloods done and will not need additional appointments or needles. These blood samples will be processed in Bristol Biobank Laboratories in the Bristol Royal Infirmary and used to help understand the causes of ITP and develop blood tests that can improve prediction of treatment responses and outcome in ITP. The aim of this additional research is to individualise treatments for ITP in the future.

What alternatives are there to taking part in the trial?

If you decide not to take part in the research trial, then you will continue standard treatment and follow up under your Haematologists supervision.

What happens when the research trial stops?

After 12 months of detailed follow up in the trial you will not need to answer any more questions and will continue on whatever treatment your haematologist recommends for you. However, we will continue collecting information from your blood tests and medical records until the trial closes.

What will happen if I don't want to carry on with the trial?

You are free to stop filling in the questionnaires at any time without giving a reason. If you wish to stop any more of your clinical data from being used in the trial you can ask for us to stop collecting it, it will not affect your care in any way. If you wish to withdraw from either of the treatment arms of the study your clinician will have to discuss with you what the alternative treatments could be, but we would still be interested to know how you are doing and would ask if you mind continuing to fill in the questionnaires and allowing us to collect your clinical data. The information we have recorded about you and the samples you have provided whilst you were on the trial may still be used. You can ask for these to be destroyed.

What will happen to the results of the research trial?

The results of the research will not be known for about 2 years after the start of the trial. The results will be reported in medical journals or presented at scientific meetings but your identity will not be disclosed.

What if relevant new information becomes available?

The research team will tell you of any new important information that may influence your participation. Your doctor may also decide to stop your participation for medical reasons even without your consent. If the trial is stopped for any other reason, you will be told why and your continuing care will be arranged.

For peer review only - http://bmjopen.bmj.com/site/about/guidelines.xhtml

Cardiff University Centre for Trials Research / Prifysgol Caerdydd Canolfan Ymchwil Treialon — University Hospitals Bristol NHS Foundation Trust

Will my taking part in the trial be kept confidential?

All information which is collected about you during the course of the research will be kept strictly confidential. This includes personal information such as your name, address and NHS number, to allow us to keep in touch with you during the trial, and information about your health, to allow us to compare the two treatment pathways. The information will be stored in a secure database at the University of Cardiff, will be anonymized and will only be accessed by authorised members of the research team who are managing the research and will be responsible for aspects of your follow-up such as arranging visits. Your medical notes will need to be seen by authorised members of the hospital research team so they can collect information needed for this research trial.

With your consent, your GP will also be informed that you are taking part in the research trial. Your GP may be asked to provide information from your records which is required for the research. Occasionally, other members of NHS staff or research staff may need to check your medical records. This will be done by NHS staff or by researchers who are bound by the same rules of confidentiality as all NHS staff. The confidentiality of your medical records will be respected at all times. Under no circumstances will you be identified in any way in any report arising from the trial.

With your consent, after your leave the study we would still like to know how you are progressing using information routinely collected in the NHS and information held by the Health and Social Care Information Centre (HSCIC) and the National Health Service Central Register (NHSCR). Any information received in this way remains confidential. The information we collect about you may be useful for future medical research in this area, and if you give permission we may include the information in other ethically approved studies. However, you can still take part in this study if you would prefer that your data was not used for other research.

Computer and paper records relating to our study will be stored for 15 years according to the provisions of the Data Protection Act.

What if there is a problem?

If you are harmed by taking part in this research project, there are no special compensation arrangements. If you are harmed due to someone's negligence, then you may have grounds for a legal action but you may have to pay for it. If you wish to complain about any aspect of the way you have been approached or treated during the course of this trial, the normal National Health Service complaints mechanisms are available to you.

Who is organising and funding the research?

The research is funded by the National Institute for Health Research (research part of the NHS). University Hospitals Bristol NHS Foundation Trust has overall responsibility for the conduct of the trial. The research is being organised and run on their behalf by the Centre for Trials Research, University of Cardiff.

Who has looked at the trial?

All research in the NHS is looked at by an independent group of people, called a Research Ethics Committee, to protect your safety, rights, wellbeing and dignity. This trial has been reviewed and given a favourable opinion by South West – Central Bristol Research Ethics Committee.

You can obtain general advice on ITP and its treatment from The ITP Support Association
http://www.itpsupport.org.uk/ Tel 01234 376559

For peer review only - http://bmjopen.bmj.com/site/about/guidelines.xhtml

You can obtain general information on clinical research from the UK Clinical Research Collaboration (UKCRC) who produces a booklet called “Understanding Clinical Trials”. This provides in-depth information on the design and conduct of clinical trials and aims to answer the questions of those considering taking part.

Electronic copies can be downloaded from the UKCRC website: http://www.ukcrc.org/patients-and-public/public-awareness-of-clinical-research/information-resources-on-clinical-research/

Printed copies can be requested by emailing: crncc.info@nihr.ac.uk

Or contacting: (Tel: 020 7395 2271)
UK Clinical Research Collaboration
C/O Medical Research Council, One Kemble Street, London,WC2B 4TS

For peer review only

Thank you for taking time to read this information

For peer review only - http://bmjopen.bmj.com/site/about/guidelines.xhtml

Cardiff University Centre for Trials Research / Prifysgol Caerdydd Canolfan Ymchwil Treialon

University Hospitals Bristol NHS Foundation Trust

Further information about taking steroids (Prednisolone or dexamethasone)

What are steroids? Some steroids occur naturally in the human body. Man-made steroids act like natural steroids to suppress the immune system and reduce inflammation. They are used in many different autoimmune illnesses (e.g. arthritis, asthma).

What are the risks and possible side effects of taking steroids?

The most common side-effects are:

- weight gain and/or increase in appetite
- Difficulty sleeping and mood changes- you may feel very high or very low. This change may be more common in people with a previous history of mood disturbance. If you're worried please discuss this with your doctor
- Increased blood sugar and blood pressure – If you have diabetes, high blood pressure or epilepsy, steroids can sometimes make these worse. Your doctor should check your blood pressure and blood sugar levels from time to time, and may adjust your medication if necessary.
- Indigestion, stomach pains
- A round face, stretch marks, thinning of the skin.
- Steroid tablets can also make glaucoma worse or cause cataracts with long term use. It may also cause muscle weakness or occasionally interfere with the menstrual cycle.
- Taking steroid tablets can make you more likely to develop infections.
- If you feel feverish or unwell, or develop any new symptoms after starting taking steroid tablets it's important to tell your doctor or specialist nurse. You should also see your doctor if you develop chickenpox or shingles or come into contact with someone who has chickenpox or shingles. These can be severe in people on steroids, and you may need antiviral treatment.
- You should avoid live vaccines (e.g. shingles)
- Steroids can cause your bones to weaken, and make fractures more likely; this can lead to a condition known as osteoporosis. Regular exercise (especially weight-bearing) can help to reduce the risk of getting osteoporosis, as can making sure you get enough calcium in your diet (dairy products) and avoiding smoking and drinking too much alcohol.
- If you have any concerns about your treatment or its side-effects you should discuss these with your doctor, nurse or pharmacist.

Treatment in the Steroid only pathway:
Prednisolone (steroid) once daily.
Initial dose of 1mg/kg (max 100mg) for 4 days
Then 40mg daily for 2 weeks
Then 20mg daily for 2 weeks
Then 10mg daily for 2 weeks
Then 5mg daily 2 weeks
Then 5mg alternate days for 2 weeks, then stop*.
***Dexamethasone** (a different type of steroid) 20mg or 40mg daily for 4 days is an alternative option to prednisolone. Your doctor may think this is more suitable for you.
For the duration of steroid, patients will get a medication to protect against stomach irritation.

For peer review only - http://bmjopen.bmj.com/site/about/guidelines.xhtml

Cardiff University Centre for Trials Research / Prifysgol Caerdydd Canolfan Ymchwil Treialon

University Hospitals Bristol NHS Foundation Trust

Further information to know about taking Mycophenolate (MMF).

What is mycophenolate? Mycophenolate mofetil (MMF), similar to steroids is used to control autoimmune conditions including ITP by reducing the activity of the body's defence system (immune system).

What are the risks and side effects of MMF?

- If you are taking MMF and you are female you must not be pregnant or breast feeding as it can harm the baby. Therefore, you must use two reliable forms of contraception before starting MMF, during therapy and six weeks after stopping MMF (e.g. hormonal or barrier method of birth control; abstinence). Contraceptive methods that can achieve a failure rate of less than 1% per year when used consistently and correctly are considered as highly effective birth control methods. Such methods include:

  - combined (oestrogen and progestogen containing) hormonal contraception associated with inhibition of ovulation:
    - oral
    - intravaginal
    - transdermal
  - progestogen-only hormonal contraception associated with inhibition of ovulation
    - oral
    - injectable
    - implantable*
  - intrauterine device (IUD)*
  - intrauterine hormone-releasing system (IUS)*
  - bilateral tubal occlusion*
  - vasectomised partner*
  - sexual abstinence defined as refraining from heterosexual intercourse during the entire period of risk associated with the trial treatments.

  **These contraception methods are considered to be low user dependency.*

  NB: Women are considered not of child bearing potential if they are surgically sterile (i.e. they have undergone a hysterectomy, bilateral tubal ligation, or bilateral oophorectomy) or they are postmenopausal.

- If you are male, with a partner of child bearing potential, you must agree to use condoms for the duration of MMF and 3 months after stopping. Condom use applies for both reproductively competent and vasectomised men, because the risks associated with the transfer of seminal fluid also apply to men who have had a vasectomy. In addition, female partners of male patients treated with MMF are recommended to use highly effective contraception during treatment and for a total of 3 months after the last stop of MMF.
- The research team must be informed immediately if you or your partner become pregnant. Women who become pregnant must stop the MMF straight away. The pregnancy will be monitored until its conclusion for safety reasons and the research team may require access

For peer review only - http://bmjopen.bmj.com/site/about/guidelines.xhtml

to mother's and/or child's notes, and any possible follow up of the child including post-natal examinations. You will be asked to consent for this eventuality.

- If you are taking MMF, it is important not to drink more alcohol than the government recommended safe limits – no more than 14 units per week. It's strongly recommended to have alcohol free days, without 'saving up' units to drink in one go.
- If you're taking MMF it is recommended that you avoid live vaccines such as yellow fever or shingles.
- Mycophenolate has been used in tens of thousands of people with ITP as second line treatment and is generally, an effective, well tolerated and acceptably safe medication. However, some patients have experienced side effects. The most common side-effects of MMF are nausea (feeling sick), diarrhoea, vomiting or stomach pain.
- Mycophenolate can also affect your blood count (one of the effects is that fewer blood cells are made) and can make you more likely to develop infections.
- You should tell your doctor or nurse specialist straight away if you develop any of the following after starting mycophenolate:
    - a sore throat
    - a fever
    - any other symptoms of infection
    - any other new symptoms or
    - anything else that concerns you.
- If any of the symptoms listed above are severe, you should stop taking MMF and see your doctor immediately. Generally, however, it's best to talk to your doctor before stopping or reducing MMF.
- You should also see your doctor if you develop chickenpox or shingles or come into contact with someone who has chickenpox or shingles. These infections can be severe if you're taking MMF. You may need antiviral treatment, and MMF may be stopped until you're better.
- Although this is uncommon, there's a slightly increased risk of certain types of cancer in people using MMF for long term use. Please discuss this matter with your doctor if you're worried. Due to the small increase in risk of skin cancer, you should avoid exposure to strong sunlight and protect your skin with sunblock or sunscreen.
- Because MMF can affect your blood count, and rarely cause liver or kidney problems, your doctor will arrange for you to have a blood test before you start treatment and regular blood checks while on MMF.
- For advice on avoiding infection from food, visit: http://www.nhs.uk/conditions/food-poisoning/pages/prevention.aspx
- There is a very small possibility of previously unknown side effects from MMF. We will take every precaution possible to monitor you for all side effects and will encourage you to report anything of concern to the research team on <Tel. Number>.

Treatment in the MMF + steroid pathway:
Prednisolone or dexamethasone (regime identical to standard care pathway as above)
From randomisation (alongside steroid), MMF 500mg twice daily starting dose.
Increased to 750mg twice a day after 2 weeks if no side effects and 1g twice daily after another 2 weeks if no side effects (4 weeks after starting).

For peer review only - http://bmjopen.bmj.com/site/about/guidelines.xhtml

Cardiff University Centre for Trials Research
Prifysgol Caerdydd Canolfan Ymchwil Treialon
University Hospitals Bristol NHS Foundation Trust

After 6 months of MMF therapy, all patients who have very well controlled ITP will reduce the dose by 250mg (one capsule) each month. The aim is to continue on the lowest dose that achieves a safe platelet count and to ensure that patients do not continue to take the drug long term if they don't need it.

In both groups

If you have already taken steroid for a few days before joining the trial, this amount will be deducted from the regime above.

If your doctor thinks you need emergency or rescue treatments then it is fine to have these in this trial. These may include platelet transfusions, tranexamic acid and intravenous immunoglobulin.

If the first course of treatment doesn't work, then your haematology doctor will discuss with you and decide what the best next treatment is for you based on individual circumstances.

For peer review only

For peer review only - http://bmjopen.bmj.com/site/about/guidelines.xhtml

Centre for Trials Research
Canolfan Ymchwil Treialon

University Hospitals Bristol NHS
NHS Foundation Trust

FLIGHT: Extra blood samples.
Additional laboratory study (optional) – Sample Collection and Storage

Patient Information Sheet

Chief Investigator
Dr Charlotte Bradbury, University of Bristol

For peer review only

You are being invited to provide additional blood samples for a research study and for future research because you have a diagnosis of Immune Thrombocytopenia (ITP). Before you decide, it is important for you to understand why the research is being done and what it will involve. Please take time to read the following information carefully and discuss it with others if you wish. One of our team will go through the information sheet with you and answer any questions you have. We would suggest this should take about 10 minutes.

Ask us if there is anything that is not clear or if you would like more information and take time to decide whether or not you wish to take part.

What will you do with the additional blood samples?
We want to improve our understanding of the causes of ITP and why some patients bleed and others don't, why some patients have a short mild illness and others have a more prolonged difficult illness.

We are also developing blood tests to help predict responses to treatment in individual patients. If successful this would help us avoid treatments that aren't going to make patients better, which will reduce side effects and reduce the time patients are unwell by choosing the most effective treatment sooner.

To do this research we need to collect blood samples from patients like you who have ITP and need to start some treatment.

Do I have to take part?
It is up to you to decide whether or not to take part. We will describe the study and go through this information sheet. If you agree to take part, we will then ask you to sign a consent form and we will take blood samples. If you decide to take part you are free to withdraw without giving a reason at any time up to the point where we have taken the blood sample. A decision to withdraw or not to take part, will not affect any aspect of your care.

You can still take part in the Flight study but decline to have these extra blood samples done.

What will happen to me if I take part and what will happen to the samples I give?
We will need a small quantity of your blood (Usually 10-30 ml up to a maximum of 50ml or a quarter of a teacup) at the start of treatment, then 2 months after starting. The blood sample will be taken when you have routine bloods done and will not involve any additional needles or appointments.

For peer review only - http://bmjopen.bmj.com/site/about/guidelines.xhtml

Centre for Trials Research
Canolfan Ymchwil Treialon

University Hospitals Bristol NHS Foundation Trust

The blood samples will be sent to the Bristol Biobank Laboratories in the Bristol Royal Infirmary. Your samples will be labelled with a unique identifier so that we can link them to the blood results already collected by the FLIGHT study and compare laboratory results with patient outcomes. Laboratory staff will not be able to directly link the samples to you. The samples will be processed immediately after receipt by the laboratory, including extraction of DNA and RNA to be used solely for research. Any remaining samples will be stored securely in a Biobank for an indefinite amount of time for future research.

All the laboratory and patient data will be securely stored and kept strictly confidential and you will not be told the laboratory results for your blood sample.

What will I have to do?
There is nothing that you should or should not do as a result of providing additional blood samples.

What are the possible benefits of taking part?
There are no clinical benefits for any person taking part in this project.

What are the possible disadvantages and risks of taking part?
Other than providing us with an extra small volume of blood, there are no anticipated disadvantages in taking part.

What will happen to the results of the research study?
Methods developed from this study may be published in medical and scientific journals. A summary will be published in the ITP patient bulletin “the platelet” and presented at the UK ITP support association patient convention. You will not be identified in any reports or publications from the study.

What happens when the research study stops?
New technologies that can help us look at biological components are emerging all the time. We would therefore wish to store any remaining samples and would ensure that they are anonymized at the end of the FLIGHT study.

What will happen if I don’t want to carry on with the study?
If you decide you no longer want your additional blood samples used for research, they will be destroyed in accordance with standard practices.

What if there is a problem?
This research is not expected to cause harm to you. If you do wish to complain about any aspect of the way you have been approached or treated during the course of this study, the normal National Health Service complaints mechanisms are available to you.

Who is organising and funding the research?
University Hospitals Bristol NHS Foundation Trust has overall responsibility for conduct of the study. The research is led by Clinical and Haematology research teams at UHB and University of Bristol. Funding for the research is from a combination of sources including ITP charitable funding.

Who has reviewed the study?
All research involving samples from people is looked at by an independent group of individuals, called a Research Ethics Committee, to protect your interests.

For peer review only - http://bmjopen.bmj.com/site/about/guidelines.xhtml

Cardiff University Centre for Trials Research
Prifysgol Caerdydd Canolfan Ymchwil Treialon

University Hospitals Bristol NHS Foundation Trust

Thank you for taking the time to read this information sheet

For peer review only - http://bmjopen.bmj.com/site/about/guidelines.xhtml

Cardiff University Prifysgol Caerdydd — Centre for Trials Research / Canolfan Ymchwil Treialon

University Hospitals Bristol NHS Foundation Trust

FLIGHT: A multicentre randomised trial of **F**irst **L**ine treatment pathways for newly diagnosed **I**mmune **T**hrombocytopenia: Standard steroid treatment versus combined steroid and mycophenolate.

For peer review only

Patient consent form

Chief Investigator: Dr Charlotte Bradbury
Local Investigator:

STUDY ID

Please ask the patient to complete the following: *Please initial all boxes*

1. I confirm that I have read and understood the Patient Information Leaflet (dated ____/____/____, version _____). I have had the opportunity to ask questions about the study and received satisfactory answers to my questions.

   I have spoken to: ____

2. I understand that I am free to withdraw from the study at any time without giving a reason and that withdrawing from the study will not affect my medical care or legal rights.

3. I understand that if I am taking MMF and either myself or my partner are of child bearing potential, I will need to use appropriate contraception detailed in the information leaflet and inform the research team if myself or my partner becomes pregnant

4. I give permission for sections of my medical records to be looked at by the study team, the regulatory authorities and the hospital trust overseeing the research. I understand that strict confidentiality will be maintained.

5. I agree to my GP being informed of my participation in this study.

6. I give permission for my personal data to be stored for the duration of the study.

7. I understand that my doctor will provide information about my progress, in confidence to the central organisers and the trials unit at Cardiff University and that all information will be held securely and in strict confidence.

For peer review only - http://bmjopen.bmj.com/site/about/guidelines.xhtml

Cardiff University — Centre for Trials Research
Prifysgol Caerdydd — Canolfan Ymchwil Treialon

University Hospitals Bristol NHS
NHS Foundation Trust

8. I agree to take part in this study.

______________________________ ______________________________ ______________
Name of patient Signature Date

______________________________ ______________________________ ______________
Name of person taking consent Signature Date

1 copy for patient; 1 for research team (original); 1 to be kept with hospital notes

CONSENT FOR MY DETAILS TO BE USED IN FUTURE RESEARCH (optional)

Please initial box

I understand that my name, NHS number and date of birth will be passed to Health and Social Care Information Centre (HSCIC) and/or National Health Service Central Register (NHSCR) in order to track my health status after I leave the study. Any information received in this way remains confidential.

1 copy for patient; 1 for research team (original); 1 to be kept with hospital notes

For peer review only - http://bmjopen.bmj.com/site/about/guidelines.xhtml

Cardiff University / Prifysgol Caerdydd — Centre for Trials Research / Canolfan Ymchwil Treialon

University Hospitals Bristol NHS Foundation Trust

FLIGHT: Extra blood samples.

Additional laboratory study (optional) – Sample Collection and Storage

Patient Consent Form

Chief Investigator: Dr Charlotte Bradbury
Local Investigator:

STUDY ID

Please **initial** all boxes

1. I confirm that I have read and understand the information sheet (insert date ____/____/____, version ______) and had the opportunity to consider the information, ask questions and have had these answered satisfactorily.
I have spoken to ____
2. I understand that my participation is voluntary and that I am free to withdraw at any time without giving any reason.
3. I understand that I will not receive any individual results from my blood samples for this part of the study.
4. I agree for my samples to be used for research purposes only.
5. I agree to DNA and RNA being extracted from my blood which will be used for research purposes only.
6. I give permission for my medical data to be stored at the trials unit at Cardiff University and that all information will be held securely and in strict confidence.
7. I understand that my samples may be processed in other laboratories in other parts of the UK, but that the laboratory staff will not be able to link the samples back to me.
8. I agree for my anonymized samples to be used in future research.
9. I agree to take part in the above study.

For peer review only - http://bmjopen.bmj.com/site/about/guidelines.xhtml

Centre for Trials Research
Canolfan Ymchwil Treialon

University Hospitals Bristol NHS Foundation Trust

Name of participant	Date	Signature
Name of person taking consent	Date	Signature

1 copy for patient; 1 for research team (original); 1 to be kept with hospital notes

For peer review only

For peer review only - http://bmjopen.bmj.com/site/about/guidelines.xhtml

Reporting checklist for protocol of a clinical trial.

Based on the SPIRIT guidelines.

Instructions to authors

Complete this checklist by entering the page numbers from your manuscript where readers will find each of the items listed below.

Your article may not currently address all the items on the checklist. Please modify your text to include the missing information. If you are certain that an item does not apply, please write "n/a" and provide a short explanation.

Upload your completed checklist as an extra file when you submit to a journal.

In your methods section, say that you used the SPIRIT reporting guidelines, and cite them as:

Chan A-W, Tetzlaff JM, Altman DG, Laupacis A, Gøtzsche PC, Krleža-Jerić K, Hróbjartsson A, Mann H, Dickersin K, Berlin J, Doré C, Parulekar W, Summerskill W, Groves T, Schulz K, Sox H, Rockhold FW, Rennie D, Moher D. SPIRIT 2013 Statement: Defining standard protocol items for clinical trials. Ann Intern Med. 2013;158(3):200-207

		Reporting Item	Page Number
Title	#1	Descriptive title identifying the study design, population, interventions, and, if applicable, trial acronym	1
Trial registration	#2a	Trial identifier and registry name. If not yet registered, name of intended registry	2
Trial registration: data set	#2b	All items from the World Health Organization Trial Registration Data Set	2
Protocol version	#3	Date and version identifier	1
Funding	#4	Sources and types of financial, material, and other support	3
Roles and responsibilities: contributorship	#5a	Names, affiliations, and roles of protocol contributors	1 and 13
Roles and	#5b	Name and contact information for the trial sponsor	1

For peer review only

responsibilities: sponsor contact information			
Roles and responsibilities: sponsor and funder	#5c	Role of study sponsor and funders, if any, in study design; collection, management, analysis, and interpretation of data; writing of the report; and the decision to submit the report for publication, including whether they will have ultimate authority over any of these activities	Supplementary
Roles and responsibilities: committees	#5d	Composition, roles, and responsibilities of the coordinating centre, steering committee, endpoint adjudication committee, data management team, and other individuals or groups overseeing the trial, if applicable (see Item 21a for data monitoring committee)	Supplementary
Background and rationale	#6a	Description of research question and justification for undertaking the trial, including summary of relevant studies (published and unpublished) examining benefits and harms for each intervention	3-6
Background and rationale: choice of comparators	#6b	Explanation for choice of comparators	3-6
Objectives	#7	Specific objectives or hypotheses	6-7
Trial design	#8	Description of trial design including type of trial (eg, parallel group, crossover, factorial, single group), allocation ratio, and framework (eg, superiority, equivalence, non-inferiority, exploratory)	7
Study setting	#9	Description of study settings (eg, community clinic, academic hospital) and list of countries where data will be collected. Reference to where list of study sites can be obtained	8
Eligibility criteria	#10	Inclusion and exclusion criteria for participants. If applicable, eligibility criteria for study centres and individuals who will perform the interventions (eg, surgeons, psychotherapists)	7

For peer review only

Interventions: description	#11a	Interventions for each group with sufficient detail to allow replication, including how and when they will be administered	8, 9 and figure 1
Interventions: modifications	#11b	Criteria for discontinuing or modifying allocated interventions for a given trial participant (eg, drug dose change in response to harms, participant request, or improving / worsening disease)	Supplementary
Interventions: adherance	#11c	Strategies to improve adherence to intervention protocols, and any procedures for monitoring adherence (eg, drug tablet return; laboratory tests)	Supplementary
Interventions: concomitant care	#11d	Relevant concomitant care and interventions that are permitted or prohibited during the trial	8,9 and figure 1
Outcomes	#12	Primary, secondary, and other outcomes, including the specific measurement variable (eg, systolic blood pressure), analysis metric (eg, change from baseline, final value, time to event), method of aggregation (eg, median, proportion), and time point for each outcome. Explanation of the clinical relevance of chosen efficacy and harm outcomes is strongly recommended	9,10
Participant timeline	#13	Time schedule of enrolment, interventions (including any run-ins and washouts), assessments, and visits for participants. A schematic diagram is highly recommended (see Figure)	Table 1
Sample size	#14	Estimated number of participants needed to achieve study objectives and how it was determined, including clinical and statistical assumptions supporting any sample size calculations	11
Recruitment	#15	Strategies for achieving adequate participant enrolment to reach target sample size	11
Allocation: sequence generation	#16a	Method of generating the allocation sequence (eg, computer-generated random numbers), and list of any factors for stratification. To reduce predictability of a random sequence, details of any planned restriction (eg, blocking) should be provided in a	8

For peer review only

		separate document that is unavailable to those who enrol participants or assign interventions	
Allocation concealment mechanism	#16b	Mechanism of implementing the allocation sequence (eg, central telephone; sequentially numbered, opaque, sealed envelopes), describing any steps to conceal the sequence until interventions are assigned	8
Allocation: implementation	#16c	Who will generate the allocation sequence, who will enrol participants, and who will assign participants to interventions	8
Blinding (masking)	#17a	Who will be blinded after assignment to interventions (eg, trial participants, care providers, outcome assessors, data analysts), and how	N/A
Blinding (masking): emergency unblinding	#17b	If blinded, circumstances under which unblinding is permissible, and procedure for revealing a participant's allocated intervention during the trial	N/A
Data collection plan	#18a	Plans for assessment and collection of outcome, baseline, and other trial data, including any related processes to promote data quality (eg, duplicate measurements, training of assessors) and a description of study instruments (eg, questionnaires, laboratory tests) along with their reliability and validity, if known. Reference to where data collection forms can be found, if not in the protocol	10 and table 1
Data collection plan: retention	#18b	Plans to promote participant retention and complete follow-up, including list of any outcome data to be collected for participants who discontinue or deviate from intervention protocols	Supplementary
Data management	#19	Plans for data entry, coding, security, and storage, including any related processes to promote data quality (eg, double data entry; range checks for data values). Reference to where details of data management procedures can be found, if not in the protocol	10 and Supplementary
Statistics: outcomes	#20a	Statistical methods for analysing primary and secondary outcomes. Reference to where other	11,12

For peer review only

		details of the statistical analysis plan can be found, if not in the protocol	
Statistics: additional analyses	#20b	Methods for any additional analyses (eg, subgroup and adjusted analyses)	NA
Statistics: analysis population and missing data	#20c	Definition of analysis population relating to protocol non-adherence (eg, as randomised analysis), and any statistical methods to handle missing data (eg, multiple imputation)	Supplementary
Data monitoring: formal committee	#21a	Composition of data monitoring committee (DMC); summary of its role and reporting structure; statement of whether it is independent from the sponsor and competing interests; and reference to where further details about its charter can be found, if not in the protocol. Alternatively, an explanation of why a DMC is not needed	Supplementary
Data monitoring: interim analysis	#21b	Description of any interim analyses and stopping guidelines, including who will have access to these interim results and make the final decision to terminate the trial	12
Harms	#22	Plans for collecting, assessing, reporting, and managing solicited and spontaneously reported adverse events and other unintended effects of trial interventions or trial conduct	Supplementary
Auditing	#23	Frequency and procedures for auditing trial conduct, if any, and whether the process will be independent from investigators and the sponsor	Supplementary
Research ethics approval	#24	Plans for seeking research ethics committee / institutional review board (REC / IRB) approval	2
Protocol amendments	#25	Plans for communicating important protocol modifications (eg, changes to eligibility criteria, outcomes, analyses) to relevant parties (eg, investigators, REC / IRBs, trial participants, trial registries, journals, regulators)	Supplementary
Consent or assent	#26a	Who will obtain informed consent or assent from potential trial participants or authorised surrogates,	7 and Supplementary

For peer review only

		and how (see Item 32)	
Consent or assent: ancillary studies	#26b	Additional consent provisions for collection and use of participant data and biological specimens in ancillary studies, if applicable	10 and Supplementary
Confidentiality	#27	How personal information about potential and enrolled participants will be collected, shared, and maintained in order to protect confidentiality before, during, and after the trial	Supplementary
Declaration of interests	#28	Financial and other competing interests for principal investigators for the overall trial and each study site	3
Data access	#29	Statement of who will have access to the final trial dataset, and disclosure of contractual agreements that limit such access for investigators	Supplementary
Ancillary and post trial care	#30	Provisions, if any, for ancillary and post-trial care, and for compensation to those who suffer harm from trial participation	Supplementary
Dissemination policy: trial results	#31a	Plans for investigators and sponsor to communicate trial results to participants, healthcare professionals, the public, and other relevant groups (eg, via publication, reporting in results databases, or other data sharing arrangements), including any publication restrictions	2
Dissemination policy: authorship	#31b	Authorship eligibility guidelines and any intended use of professional writers	NA
Dissemination policy: reproducible research	#31c	Plans, if any, for granting public access to the full protocol, participant-level dataset, and statistical code	Supplementary
Informed consent materials	#32	Model consent form and other related documentation given to participants and authorised surrogates	Supplementary
Biological specimens	#33	Plans for collection, laboratory evaluation, and storage of biological specimens for genetic or molecular analysis in the current trial and for future use in ancillary studies, if applicable	Supplementary

For peer review only

The SPIRIT checklist is distributed under the terms of the Creative Commons Attribution License CC-BY-ND 3.0. This checklist can be completed online using https://www.goodreports.org/, a tool made by the EQUATOR Network in collaboration with Penelope.ai

For peer review only

BMJ Open

A multicentre randomised trial of First Line treatment pathways for newly diagnosed Immune Thrombocytopenia: Standard steroid treatment versus combined steroid and mycophenolate. The FLIGHT trial

Journal:	*BMJ Open*
Manuscript ID	bmjopen-2018-024427.R1
Article Type:	Protocol
Date Submitted by the Author:	06-Aug-2018

Complete List of Authors:	Pell, Julie; Cardiff University Centre for Trials Research<br>Greenwood, Rosemary; University Hospitals Bristol NHS Foundation Trust, Research and Design Service<br>Ingram, Jenny; Bristol University , School of Social & Community Medicine<br>Wale, Katharine; Research and Innovation, University Hospitals Bristol<br>Thomas, Ian; Cardiff University Centre for Trials Research<br>Kandiyali, Rebecca; Research and Innovation, University Hospitals Bristol<br>Mumford, Andrew; University Hospitals Bristol NHS Foundation Trust, Department of Haematology<br>Dick, Andrew; University of Bristol<br>Bagot, Catherine; Glasgow Royal Infirmary<br>Cooper, Nichola; Imperial College London<br>Hill, Quentin; Leeds Teaching Hospitals NHS Trust<br>Bradbury, Charlotte; University of Bristol, Cellular and Molecular Medicine; University Hospitals Bristol NHS Foundation Trust, Haematology

**Primary Subject Heading**:	Haematology (incl blood transfusion)
Secondary Subject Heading:	Immunology (including allergy)
Keywords:	Immune Thrombocytopenia, ITP, Prednisolone, mycophenolate, Dexamethasone, corticosteroid

SCHOLARONE™ Manuscripts

TITLE: A multicentre randomised trial of First Line treatment pathways for newly diagnosed Immune Thrombocytopenia: Standard steroid treatment versus combined steroid and mycophenolate. The FLIGHT trial

Authors: Julie Pell[1], Rosemary Greenwood[2], Jenny Ingram[2], Katherine Wale[3], Ian Thomas[1], Rebecca Kandiyali[3], Andrew Mumford[4,5], Andrew Dick[4,6], Catherine Bagot[7], Nichola Cooper[8], Quentin Hill[9], Charlotte Bradbury[4,5]

1 Centre for Trials Research, Cardiff University, PellJC@cardiff.ac.uk, thomasif@cardiff.ac.uk,

2 Research Design Service – South West, University of Bristol, rosemary.greenwood@UHBristol.nhs.uk, jenny.ingram@bristol.ac.uk,

3 Research & Innovation, University Hospitals Bristol NHS Foundation Trust, katharine.Wale@UHBristol.nhs.uk, rebecca.kandiyali@uhbristol.nhs.uk,

4 Cellular and Molecular Medicine, University of Bristol, a.mumford@bristol.ac.uk, a.dick@bristol.ac.uk, c.bradbury@bristol.ac.uk,

5 Department of Haematology, University Hospitals Bristol NHS Foundation Trust

6 UCL-Institute of Ophthalmology

7 Glasgow Royal Infirmary, Glasgow, Catherine.Bagot@ggc.scot.nhs.uk,

8 Imperial College London and Hammersmith Hospital, London, n.cooper@imperial.ac.uk,

9 Leeds Teaching Hospitals NHS Trust, quentinhill@nhs.net,

KEYWORDS

Immune Thrombocytopenia, Prednisolone, Mycophenolate, Corticosteroid

Corresponding author: Dr Charlotte Bradbury, Level 8, Bristol Haematology & Oncology Centre, Horfield Road, Bristol, BS2 8ED c.bradbury@bristol.ac.uk

Version: V3.0, 24-Oct 2017

Trial sponsor: University Hospitals Bristol NHS Foundation Trust, Research and Innovation, Level 3, UH Bristol Education and Research Centre, Upper Maudlin Street, Bristol BS2 8AE

Word Count: 4058

For peer review only

ABSTRACT

Introduction

Immune thrombocytopenia (ITP) is an autoimmune condition that may cause thrombocytopenia related bleeding. Current first line ITP treatment is with high dose corticosteroids but frequent side effects, heterogeneous responses and high relapse rates are significant problems with only 20% remaining in sustained remission with this approach. Mycophenolate (MMF) is often used as the next treatment with efficacy in 50-80% of patients and good tolerability but can take up to 2 months to work.

Objective: To test the hypothesis that MMF combined with corticosteroid is a more effective first line treatment for immune thrombocytopenia (ITP) than current standard of corticosteroid alone.

Methods and analysis

Design: Multicentre, UK based, open label, randomised controlled trial

Setting: Haematology departments in secondary care

Participants: We plan to recruit 120 patients >16 years old with a diagnosis of ITP and a platelet count <30 x10^9/L who require first line treatment. Patients will be followed up for a minimum of 12 months following randomisation.

Primary outcome: Time from randomisation to treatment failure defined as platelets <30x10^9/L and a need for 2nd line treatment.

Secondary outcomes: Side effects, bleeding events, remission rates, time to relapse, time to next therapy, cumulative corticosteroid dose, rescue therapy, splenectomy, socioeconomic costs, patient reported outcomes (quality of life, fatigue, impact of bleeding, care costs).

Analysis: The sample size of 120 achieves a 91.5% power to detect a doubling of the median time to treatment failure from 5 to 10 months. This will be expressed as a hazard ratio with 95% confidence interval, median time to event if more than 50% have had an event and illustrated with Kaplan Meier curves. Cost effectiveness will be based on the first 12 months from diagnosis.

Ethics and dissemination

Ethical approval from NRES Committee South West (IRAS number 225959). EudraCT Number: 2017-001171-23. ClinicalTrials.gov number: NCT03156452. Results will be submitted for publication in peer-reviewed journals.

For peer review only

STRENGTHS AND LIMITATIONS OF THIS STUDY

- First UK multicentre RCT for first line treatment of ITP
- Independent funding from NIHR testing a pragmatic, cost effective approach which if effective, may be applicable to other autoimmune conditions
- The trial includes patient-oriented outcomes by using validated questionnaires to assess quality of life, fatigue, impact of bleeding and care costs
- Option to consent to additional blood samples for translational research to maximise scientific potential
- The limitations include the open label design, lack of very long term follow up and sample size to detect only moderate differences between treatment arms

FUNDING

The FLIGHT trial is independent research funded by the National Institute for Health Research (NIHR) under its Research for Patient Benefit (RfPB) Programme (Grant Reference Number PB-PG-0815-20016). The views expressed are those of the authors and not necessarily those of the NHS, the NIHR or the Department of Health.

CONFLICTS OF INTEREST

None of the authors have competing interests

INTRODUCTION

Immune thrombocytopenia (ITP) has an incidence of 2.9/100,000 person-years. [1] It is an autoimmune condition that may present with bleeding and bruising due to a low platelet count. In ITP, there is increased consumption and reduced production of platelets due to both antibody and cell mediated autoimmune attack of platelets and megakaryocytes involving dysregulated autoreactive T and B cell lymphocytes. [2-5]

ITP can be classified according to the duration of illness into newly diagnosed (<3 months), persistent (3-12 months) and chronic (>12 months)[6]. ITP may also be classified as either primary when it presents in isolation or secondary when ITP occurs in the context of an associated illness or medication[6].

For peer review only - http://bmjopen.bmj.com/site/about/guidelines.xhtml

ITP is a diagnosis of exclusion and made when the platelet count <100x 109/L and other causes of thrombocytopenia are excluded by history, examination and laboratory evaluation[6 7].

Current first line ITP treatment is with high dose corticosteroids but this has several downsides. First, the majority of patients suffer significant side effects including mood swings, difficulty sleeping, weight gain, high blood pressure, diabetes, gastric irritation, skin thinning and osteoporosis. A published survey of ITP patients reported 98% had at least one side effect and 38% stopped or reduced dosage due to intolerable side effects.[8] In the UK ITP registry, the most frequently reported co-morbidities were related to corticosteroids and correlated with duration of treatment (hypertension in 30%, diabetes in 19%) (Newland A et al, poster BSH 2015[25]). The second problem is that patients are heterogeneous in their response to corticosteroid with some (approximately 20%) not responding at all and the majority of others (70%-90%) relapsing when the corticosteroids are reduced or stopped. [7 9 10] Patients who are refractory or relapse (the majority), remain at risk of bleeding/bruising, which occasionally can be severe including intracranial haemorrhage.[11] They often receive more corticosteroid with associated side effects. Some require hospital admission and expensive rescue therapies (e.g. IVIG for a 70kg patient=£3,906). They continue to require frequent blood tests and doctor visits and are usually unable to continue their normal activities until their illness is controlled. Fatigue is also associated with disease activity and can be severe. [12] Physical factors combine with psychological stress through fear of bleeding, need for time off work and lifestyle restrictions due to bleeding risk to adversely impact quality of life.[13 14]

First line treatment for ITP is unsatisfactory but it remains unchanged for decades. Although a small number of studies have tested alternative approaches, a well- tolerated, effective and durable new approach has not been conclusively demonstrated. High dose corticosteroid remains the standard first line treatment recommended in most countries[9].

Compared to cancers in haematology, ITP remains relatively under-researched. The few trials done in ITP have often been funded by pharmaceutical companies, risking publication bias towards high cost non-generic drugs. For example, many “cheap”, generic drugs commonly prescribed for ITP such as azathioprine, mycophenolate and dapsone, have never been tested in randomised controlled trials in ITP. In contrast, the more expensive treatments, TPO-RAs and Rituximab have been tested in well- designed adequately powered RCTs. The relative rarity (2.9/100,000 person-years), non-cancerous nature and rare impact on survival of ITP have prevented ITP being a priority for research funding in the past. However, this underestimates the profound adverse impact a

For peer review only - http://bmjopen.bmj.com/site/about/guidelines.xhtml

diagnosis of ITP and its treatment can have for individual patients, many of whom are young. There is also a costly financial impact for the NHS from the healthcare resources patients require when their illness is uncontrolled. In addition, the problems faced by patients with ITP mirror those with other autoimmune conditions which as a group are common, affecting 3% of the population. There is an urgent clinical need to address this inequality, improving first line treatment for ITP through high quality, independently funded research to allow patients with this condition access to improvements in care seen in other illnesses such as cancer or heart disease.

Current popular options for second line or subsequent treatment include Mycophenolate, Rituximab, Thrombopoeitin receptor agonists (TPO RA) and splenectomy. Splenectomy is an effective treatment (60% long term remission rates) but irreversible and international guidelines recommend deferring splenectomy for the first 12 months following diagnosis due to the chance of spontaneous remission (risk of unnecessarily removing a healthy organ).[7 10] Surgical operations are not popular with patients and there is increasing awareness of the short and long term complications of splenectomy including infection, bleeding, arterial and venous thrombosis, cancer and relapse.[15] The splenectomy numbers performed in the UK has dramatically reduced over recent years (UK ITP registry data). Rituximab is a monoclonal antibody treatment which targets antibody production by B cells. It is relatively expensive, with disappointing long term remission rates similar to placebo.[15] TPO RA stimulate platelet production, are well tolerated and effective in the majority[16] but at significant financial cost, prohibiting widespread use in the UK for early treatment (NICE guidance). A small (n=12) non-randomised study using TPO RA with corticosteroid first line showed efficacy but perhaps less than expected.[17] By contrast, Mycophenolate (MMF) is a widely used second line agent in the UK due to good efficacy (response rates of 50-80%), safety and tolerability profile.[18-25] Mycophenolate has activity against both autoreactive T and B cells and has also shown efficacy in refractory ITP including steroid resistance suggesting a complimentary mechanism of action. [23] It is less expensive to the NHS than some other second line options costing approximately £182/year (generic cost) compared to costs for average doses of romiplostim (TPO RA) at £25 000/year, Eltrombopag (TPO RA) £20 000/year or rituximab at £8000 for a course of 4 infusions (375mg/m2 each dose) or £1000 (100mg each dose). However, similar to other second line therapies, MMF has a relatively slow (up to 2 months) onset of action. In the meantime, patients often receive further steroid (to maintain a "safe platelet count") and continue to suffer problems associated with their illness (see above). Direct feedback from patients regarding the difficulties they face in the first months following ITP diagnosis has been the primary driving force for this

For peer review only - http://bmjopen.bmj.com/site/about/guidelines.xhtml

clinical trial. Local (Bristol) and national patient groups (ITP support association) have been fundamental to the formulation of patient relevant priorities for treatment.

Rationale

The Flight trial is the first UK, NHS coordinated, Pharma independent multicentre randomised controlled trial, testing a “common sense/practical” new approach using MMF first line instead of second line with the aim of preventing the almost inevitable first relapse when corticosteroids stop. Patients will be randomly allocated to one of 2 treatment arms, either standard of care (corticosteroid alone) or MMF combined with corticosteroid with the primary outcome of time to treatment failure. By giving patients a stable platelet count sooner, we expect to improve other outcomes such as quality of life and fatigue. By reducing the risk of relapse, patients may also be less likely to receive a second course of corticosteroid with associated side effects. Potential indirect benefits to the NHS include reduced need for rescue treatments, blood tests, hospital attendances and admissions and reduced need for high cost treatments such as TPO mimetics. However, there will be some patients who will be treated with MMF who may have been successfully treated with corticosteroids alone (10-30%).[7-9] Similar to other immunosuppressives, MMF may slightly increase infection and cancer risk with long term use (SmPC) In addition, MMF is teratogenic and therefore stringent pregnancy prevention is essential for men and women taking the drug. This puts the trial in equipoise. The trial includes a strategy to reduce and stop MMF at 6 months for patients in complete remission to prevent unnecessary long-term use.

The choice of this open label design was made in order to allow true patient treatment costs to be calculated for the cost effectiveness analysis, and to deal with the complexities of placebo controlling a drug that needed titrating at the start and tapering at the end. In addition, over encapsulation was only possible for the lower MMF dose (250 mg) and the resulting capsule was the largest size which would mean most patients taking 8 large capsules per day in both arms; something that patients in Bristol thought would put them off taking part in the study. Patients were clear that from their perspective that a straight forward open label design would be preferable and was easier for a new patient to understand and consent. In addition, the quotes from 2 separate companies also showed the financial costs of encapsulation to generate a placebo were prohibitively expensive.

This trial proposal has received support and input from clinicians and patients nationally (UK ITP forum and ITP support association). To ensure objective and meaningful outcomes, it will be a

For peer review only - http://bmjopen.bmj.com/site/about/guidelines.xhtml

multicentre RCT, aiming to recruit 120 patients (expecting 100 full datasets). Patients will be given up to one week of corticosteroid prior to randomisation to enable sufficient time to read information, discuss and ask questions with informed consent in an appropriate setting. Patients will receive the usual follow up according to clinical need and local policy. Laboratory and clinical data will be collected from routine appointments. In addition, patient-oriented outcomes will be recorded at diagnosis, 2, 4, 6, and 12 months using validated patient questionnaires. Patients are also offered consent to additional blood samples for translational research studies (time 0 and 2 months).

Primary objective

To compare two first line treatment pathways for ITP, standard corticosteroid only versus corticosteroid combined with MMF and demonstrate which pathway helps patients achieve a stable platelet count sooner, measured as survival free from treatment failure (time from randomisation to treatment failure).

METHODS AND ANALYSIS

Trial design

A multicentre, open label randomised clinical trial of MMF with corticosteroid as first line treatment for patients with ITP versus the standard care pathway of corticosteroids alone as first line treatment.

Eligibility criteria

Inclusion criteria: Patients >16 years old with a diagnosis of ITP (primary or secondary), a platelet count <$30x10^9$/L AND a clinical need for first line treatment. Patients have provided written informed consent.

Patients can be recruited at any time after ITP diagnosis if they are suitable for first line treatment (i.e. Not previously or recently treated). Patients can receive up to 1 week of corticosteroid prior to recruitment to allow time to be informed about the trial, with the opportunity to ask questions and for consent to be taken during a routine specialist clinic appointment if preferred.

Exclusion criteria: Pregnancy and breastfeeding (Women of child bearing potential require a pregnancy test result within 7 days prior to randomisation to rule out unintended pregnancy). Patients with HIV, Hepatitis B or C, or Common Variable immunodeficiency. Contraindications to

For peer review only - http://bmjopen.bmj.com/site/about/guidelines.xhtml

MMF or corticosteroid (see SmPC) including patients with active significant infections, hypersensitivity to mycophenolate mofetil, mycophenolic acid or to any of the excipients or active significant infection. Patients not capable of giving informed consent (e.g. due to incapacity). Patients (men and women) unwilling to follow contraceptive advice if allocated to MMF treatment arm

Study setting

120 patients will be recruited from approximately 40 Haematology departments of hospitals (secondary care) across the UK where ITP patients are treated.

The trial processes will be run by the Centre for Trials Research (CTR), Cardiff University and sponsored by University Hospitals Bristol NHS Foundation Trust.

The flight trial opened for recruitment on 26th October 2017.

Randomisation

Patients who agree to participate will be randomised to MMF with corticosteroid or corticosteroid alone in a 1:1 ratio using a secure web based randomisation system based at Cardiff CTR. Randomisation will be stratified by primary or secondary ITP diagnosis. Due to the large number of centres and the small number of patients it will not be sensible to stratify randomisation by study centre. However, to ensure an even spread of patients across time, randomisation will be blocked using random block sizes of 6 and 8 to retain concealment.

Treatment arms (Figures 1 and 2):

1. **Corticosteroid only pathway (Figure 1):** 1mg/kg od prednisolone 4 days (maximum of 100mg), 40mg od 2 weeks, 20mg od 2 weeks, 10mg od 2 weeks, 5mg od 2 weeks then 5mg alternate days 2 weeks then stop*. For the duration of steroid, patients will get a PPI or H2 antagonist to protect against gastric bleeding and appropriate bone protection.

*Dexamethasone 20mg or 40mg orally daily for 4 days is an alternative option to prednisolone if deemed clinically more appropriate for individual circumstances.

2. **Corticosteroid +MMF pathway (Figure 2):** 1mg/kg od prednisolone 4 days (maximum of 100mg), 40mg od 2 weeks, 20mg od 2 weeks, 10mg od 2 weeks, 5mg od 2 weeks then 5mg alternate days 2 weeks then stop*. For the duration of steroid, patients will get a PPI or H2 antagonist to protect against gastric bleeding and appropriate bone protection.

For peer review only - http://bmjopen.bmj.com/site/about/guidelines.xhtml

*Dexamethasone 20mg or 40mg daily for 4 days is an alternative option to prednisolone if deemed clinically more appropriate for individual circumstances.

From randomisation (alongside steroid), MMF 500mg bd starting dose then increased to 750mg bd after 2 weeks if tolerated (no side effects or laboratory concerns such as neutropenia) and 1g bd after another 2 weeks if tolerated (4 weeks after starting). Earlier dose escalation to MMF 1g bd can be considered if clinically indicated.

After 6 months of MMF therapy, all patients who have remained in complete remission (pl count> 100 x10 ^{9}/L) will reduce the dose by 250mg (one capsule) each month. The aim is to continue on the lowest dose that achieves a haemostatic (safe) platelet count (pl >30 x10 ^{9}/L) and to ensure that patients who have gone into a remission do not continue to take the drug indefinitely.

In both groups: Any steroid commenced prior to randomisation will be deducted from the regimes. Importantly, emergency and rescue treatments will be permitted throughout the study. These include platelet transfusions, tranexamic acid and intravenous immunoglobulin. These are known not to impact on the natural history of ITP and it is recognised that they may be important for patient safety. The use of "rescue treatments" will be recorded on the CRF.

In addition, some degree of flexibility of corticosteroid dose and duration may be needed for individual patients according to comorbidity, tolerability and other factors.

If treatment failure occurs, choice of second line treatment will be individualised according to patient's clinical circumstances. Further steroid will be given according to clinical need.

Primary outcome is time from randomisation to treatment failure defined as a pl count <30x10^{9}/L AND a need to commence second line treatment. This will include patients who are refractory (pl <30x10^{9}/L in spite of 2 weeks treatment in the steroid arm or pl <30x10^{9}/L in spite of 2 months treatment in the steroid +MMF arm) or who initially respond but then relapse (defined clinically as pl <30x10^{9}/L and need for further therapy).

Secondary outcomes

1. Medication side effects, toxicity or other adverse events (including infection episodes)
2. Bleeding events

For peer review only - http://bmjopen.bmj.com/site/about/guidelines.xhtml

   a. Site and type of bleeding
   b. Treatment required for bleeding
   c. Whether hospital admission was required
   d. Whether ITP rescue treatments were needed
3. Remission rates (pl >30 x10^9/L and at least 2 fold increase from baseline); Complete 100x10^9/L, partial 30-100x10^9/L.
4. Time to relapse and time to next therapy
5. Cumulative corticosteroid dose
6. Need for rescue therapies
7. Need for splenectomy
8. Socioeconomic costs
9. Patient reported outcomes (quality of life, fatigue, impact of bleeding, care costs).

Patients follow up

Patients will be followed up until the end of the trial and for a minimum of 12 months. They will receive the usual follow up according to clinical need and local policy. Laboratory and clinical data will be collected from routine appointments. In addition, patient oriented outcomes and additional data will be recorded at diagnosis, 2, 4, 6, and 12 months using validated patient questionnaires. Patients are also offered consent to additional blood samples for translational research studies (time 0 and 2 months).

Data collection (Table 1)

Hospital monitoring of platelet levels (FBC) is part of routine care for ITP patients and these data will be collected and recorded on the CRF without requiring patients to come in for additional samples to be taken. These locally collected samples may be collected monthly (or less often) for patients believed to be in stable remission and weekly at lower or declining platelet levels. We expect this to allow us to calculate the time in remission and time to relapse with reasonable accuracy over the 12 to 24 month follow up period. Other clinical and laboratory data needed for the trial endpoints will be collected from the medical and electronic records and recorded on the CRF. In addition, we will also ask the patients to complete questionnaires on fatigue, quality of life and bleeding scores at baseline, 2, 4, 6, and 12 months.

Patient reported outcomes will be captured by the following questionnaires:

1. SF36v2 (Your health & wellbeing) – Quality of life,
2. FACIT-Fatigue (version 4) – Fatigue,

For peer review only - http://bmjopen.bmj.com/site/about/guidelines.xhtml

3. FACT-Th6 (version 4)- Bleeding,

4. ICECAP-A V2- Quality of life,

5. Health economic/resource use questionnaire - Personal and social costs.

Additional optional research blood samples (requiring separate consent) will be sent at baseline and 2 month follow up to the Bristol Biobank.

Data Management

Source documents produced for this trial will be kept in the patient's hospital records and source data will be transcribed into trial-specific Case Report Forms (CRFs) at the end of each patient visit. Data recording for this trial will be via a web based system. This is a secure encrypted system accessed by an institutional password which complies with Data Protection Act standards. The database will be stored and regularly backed up on a Cardiff University Server. The CRFs will be coded with the study number and will not include patients' names and addresses

Patient and Public Involvement and Engagement

During the trial development, a group of 8 ITP patients discussed the study design, burden of outcome measure completion to patients and the size of a potential placebo capsule which they reported could put them off getting involved in a trial. They reported that avoidance of relapse, early achievement of a stable platelet count, reduced overall corticosteroid dose and reduced hospital attendances are the most important goals for ITP management from their perspective.

We formed a Patient Advisory Group (PAG) with some of these patients and representatives from the ITP association that will advise the trial management group throughout the study. They have commented on all patient-facing documentation and will be instrumental in disseminating the study findings to patient groups and the public.

STATISTICS AND DATA ANALYSIS

Sample size calculation

There is no published clinical data available for MMF use first line in ITP as this is a novel approach. We have analysed local data on MMF used second line in ITP in 12 patients which shows an estimated median survival free from treatment failure of more than 10 months. We have data on 68 who experienced corticosteroids as a first line treatment showing that 70% of them had experienced a treatment failure by 12 months and that the median survival free from treatment failure was 5.0 months (95% CI [3.2, 6.8]). Data for the 12 patients treated with MMF second line therapy have

For peer review only - http://bmjopen.bmj.com/site/about/guidelines.xhtml

shorter follow-up times, with only 5 patients having follow up beyond 12 months. The cox proportional hazards ratio model demonstrates the 90% confidence interval for the hazard ratio to be between 0.13 and 0.59, showing that our decision to power this on an estimate of a hazard of lower than 0.5 is potentially achievable.

Clinically a doubling in the time to remission was thought to be something that the patients would have welcomed. Less than that was not thought to be sufficient grounds for switching this treatment from second line to first line due to the potential for additional toxicity and immune suppression in those who may have remained in remission with corticosteroids alone.

The sample size of 120 (60 per group) with less than 5% loss to follow-up achieves 91.5% power to detect a doubling of the median time to treatment failure from 5 months to 10 months if the patients are recruited at a steady rate or 10 per month for 12 months and all followed up until the last patients reaches 12 months follow up.

Statistical analysis

The full statistical analysis will be written into a statistical analysis plan available separately. The analysis will produce a CONSORT diagram for the reporting of clinical trials.

The baseline characteristics of the two groups will be tabulated but not tested for statistically significant differences between the groups.

The primary analysis is by intention to treat. However an investigation of compliance with the treatment pathway and compliance with the criteria for changing to a second line therapy will be carried out prior to the primary analysis to check the date of the primary event. The primary event is the date at which there was a requirement for second line therapy. Where the platelet count falls below the level required for this treatment decision, the first date at which either symptoms or a blood test revealing this event will be used. If a clinician decides to use a second line therapy without a platelet count below the criteria, the date of the treatment decision/new prescription will be taken to represent that event. The results will be expressed as a hazard ratio with 95% confidence interval, median time to event if more than 50% have had an event and plotted as Kaplan Meier curves.

For peer review only - http://bmjopen.bmj.com/site/about/guidelines.xhtml

The primary analysis will contain all patients who are randomised for as long as they have been followed up or until their first event in a survival analysis using intention to treat methodology. All patients will be followed up to 12 months. In addition, patients who have not had an event in the first 12 months post randomisation will be followed until their first event or until the last patient has reached the 12 month point – whichever is the sooner and included in the analysis until that time accordingly.

Analysis of other outcomes will use as full a data set as possible and focus on the 12 month data point or area under the curve as appropriate and detailed in the analysis plan.

No interim analyses of the main endpoint will be supplied to the independent Data Monitoring Committee (DMC) due to the short time frame (12 months recruitment) in which all patients will be recruited by the time the first patient has completed follow-up. Serious adverse event rates will be reported on a monthly basis to the trial management group (TMG) and the DMC. The DMC could advise the chairman of the Trial Steering Committee and Chief Investigator if these provide proof beyond reasonable doubt that it would be unethical to continue with the trial.

Pharmacovigilance

The collection and reporting of all adverse events is in accordance with the Medicines for Human Use Clinical Trials Regulations 2004 and its subsequent amendments and follows the standard operating procedures of the trials unit, Cardiff University Centre for Trials Research (CTR). Seriousness and causality are assessed by participating sites and further review of expectedness (based on the reference safety information) is conducted centrally on behalf of the Sponsor. Events are defined as serious adverse events (SAEs), serious adverse reactions (SARs) or suspected unexpected serious adverse reaction (SUSARs) in line with regulatory definitions on the basis of these assessments.

SAEs are reported throughout the treatment period up to 6 weeks after cessation of last dose of MMF. SARs should continue to be reported until the end of follow up. All deaths and overdoses are reported to the Sponsor as an SAE and reviewed in line with other events.

MMF in this trial has a genotoxic and teratogenic potential and therefore pregnancy is contraindicated. Participants that are female of child bearing potential, or male with female partners of equal potential, are required to use contraception as indicated in the protocol.

For peer review only - http://bmjopen.bmj.com/site/about/guidelines.xhtml

Pregnancy, or the pregnancy of a partner occurring whilst participating in the trial, is not considered an SAE, however, a congenital anomaly or birth defect is. Pregnancy is reported to Sponsor and followed up to outcome.

All safety events are reviewed by the Trial Management Group on an ongoing basis and reported to the Trial Steering Committee for oversight. Overall assessment of the safety profile of both arms will be included in final reporting and publication.

Author Contributions: Charlotte Bradbury is chief investigator for the Flight trial and was responsible for writing the protocol with clinical input from Nichola Cooper, Quentin Hill and Catherine Bagot. Rosemary Greenwood is the trial statistician who has contributed to the trial design and writing of protocol. Jenny Ingram contributed to trial design and leads the Patient Advisory Group input. Rebecca Kandiyali is the trial health economist and provided input to the trial design and protocol. Julie Pell and Ian Thomas (Cardiff CTR) and Katharine Wale (sponsor representative) have also provided contribution to writing the protocol. Andrew Mumford and Andrew Dick have provided mentorship to the Chief investigator.

Acknowledgements: We would like to thank the members of the UK ITP forum who have been invaluable providing feedback on the trial design and recruitment to the trial.

Figure 1. Flight treatment pathway: Corticosteroid only

Figure 2. Flight treatment pathway: Corticosteroid and Mycophenolate

For peer review only - http://bmjopen.bmj.com/site/about/guidelines.xhtml

Table 1: Time schedule of enrolment, interventions, assessments and visits

**Procedures**	**V0**	**V1**	**V2 (2 mths)**	**V3 (4 mths)**	**V4 (6 mths)**	**V5 (12 mths)**	**V6 12-24 mths**
	**Screen**	**Baseline / Randomisation to pathway 1 or 2**	**Follow up**	**Follow up**	**Follow up**	**Follow up**	**Data collection from sites**
Eligibility assessment	x						
Randomisation		x					
Informed consent		x					
Demographics		x					
Medical history		x	x	x	x	x	
Physical examination		x					
Vital signs (incl height & weight)		x	x	x	x	x	
Pregnancy test	x						
Concomitant medications		x	x	x	x	x	
Standard practice bloods (includes blood sugar if applicable)	x	x	x	x	x	x	
Hepatitis B, C & HIV serology	x						
Immunoglobulins (blood)		x			x	x	
Extra blood samples (optional)		x	x				
Dispensing of trial drugs		x*					
Compliance			x				
QofL FACT-Th6, V4	x	x	x	x	x	x	
QofL ICECAP V2 – A measure	x	x	x	x	x	x	
QofL SF-36v2 – Health Survey	x	x	x	x	x	x	
QofL FACIT-F, V4, pg 3 (fatigue)	x	x	x	x	x	x	
QofL Thrombocytopenia costs questionnaire	x	x	x	x	x	x	
Data collection from sites on platelet count & treatment		x	x	x	x	x	x

* MMF and corticosteroid dispensing frequency can follow standard local practice.

For peer review only - http://bmjopen.bmj.com/site/about/guidelines.xhtml

REFERENCES

1. Moulis G, Palmaro A, Montastruc JL, et al. Epidemiology of incident immune thrombocytopenia: a nationwide population-based study in France. *Blood* 2014;124(22):3308-15. doi: 10.1182/blood-2014-05-578336 [published Online First: 2014/10/12]
2. McKenzie CG, Guo L, Freedman J, et al. Cellular immune dysfunction in immune thrombocytopenia (ITP). *British journal of haematology* 2013;163(1):10-23. doi: 10.1111/bjh.12480 [published Online First: 2013/08/14]
3. Iraqi M, Perdomo J, Yan F, et al. Immune thrombocytopenia: antiplatelet autoantibodies inhibit proplatelet formation by megakaryocytes and impair platelet production in vitro. *Haematologica* 2015;100(5):623-32. doi: 10.3324/haematol.2014.115634 [published Online First: 2015/02/16]
4. Ji X, Zhang L, Peng J, et al. T cell immune abnormalities in immune thrombocytopenia. *Journal of hematology & oncology* 2014;7:72. doi: 10.1186/s13045-014-0072-6 [published Online First: 2014/10/03]
5. Audia S, Rossato M, Santegoets K, et al. Splenic TFH expansion participates in B-cell differentiation and antiplatelet-antibody production during immune thrombocytopenia. *Blood* 2014;124(18):2858-66. doi: 10.1182/blood-2014-03-563445 [published Online First: 2014/09/19]
6. Rodeghiero F, Stasi R, Gernsheimer T, et al. Standardization of terminology, definitions and outcome criteria in immune thrombocytopenic purpura of adults and children: report from an international working group. *Blood* 2009;113(11):2386-93. doi: 10.1182/blood-2008-07-162503 [published Online First: 2008/11/14]
7. Provan D, Stasi R, Newland AC, et al. International consensus report on the investigation and management of primary immune thrombocytopenia. *Blood* 2010;115(2):168-86. doi: 10.1182/blood-2009-06-225565 [published Online First: 2009/10/23]
8. Brown TM, Horblyuk RV, Grotzinger KM, et al. Patient-reported treatment burden of chronic immune thrombocytopenia therapies. *BMC blood disorders* 2012;12:2. doi: 10.1186/1471-2326-12-2 [published Online First: 2012/03/23]
9. Neunert CE. Management of newly diagnosed immune thrombocytopenia: can we change outcomes? *Hematology American Society of Hematology Education Program* 2017;2017(1):400-05. doi: 10.1182/asheducation-2017.1.400 [published Online First: 2017/12/10]
10. Neunert C, Lim W, Crowther M, et al. The American Society of Hematology 2011 evidence-based practice guideline for immune thrombocytopenia. *Blood* 2011;117(16):4190-207. doi: 10.1182/blood-2010-08-302984 [published Online First: 2011/02/18]
11. Neunert C, Noroozi N, Norman G, et al. Severe bleeding events in adults and children with primary immune thrombocytopenia: a systematic review. *Journal of thrombosis and haemostasis : JTH* 2015;13(3):457-64. doi: 10.1111/jth.12813 [published Online First: 2014/12/17]
12. Hill QA, Newland AC. Fatigue in immune thrombocytopenia. *British journal of haematology* 2015;170(2):141-9. doi: 10.1111/bjh.13385 [published Online First: 2015/04/01]
13. Snyder CF, Mathias SD, Cella D, et al. Health-related quality of life of immune thrombocytopenic purpura patients: results from a web-based survey. *Current medical research and opinion* 2008;24(10):2767-76. doi: 10.1185/03007990802377461 [published Online First: 2008/08/22]

For peer review only - http://bmjopen.bmj.com/site/about/guidelines.xhtml

14. Tarantino MD, Mathias SD, Snyder CF, et al. Impact of ITP on physician visits and workplace productivity. *Current medical research and opinion* 2010;26(2):319-28. doi: 10.1185/03007990903451298 [published Online First: 2009/12/09]
15. Kristinsson SY, Gridley G, Hoover RN, et al. Long-term risks after splenectomy among 8,149 cancer-free American veterans: a cohort study with up to 27 years follow-up. *Haematologica* 2014;99(2):392-8. doi: 10.3324/haematol.2013.092460 [published Online First: 2013/09/24]
16. Rodeghiero F, Ruggeri M. Treatment of immune thrombocytopenia in adults: the role of thrombopoietin-receptor agonists. *Seminars in hematology* 2015;52(1):16-24. doi: 10.1053/j.seminhematol.2014.10.006 [published Online First: 2015/01/13]
17. Gomez-Almaguer D, Herrera-Rojas MA, Jaime-Perez JC, et al. Eltrombopag and high-dose dexamethasone as frontline treatment of newly diagnosed immune thrombocytopenia in adults. *Blood* 2014;123(25):3906-8. doi: 10.1182/blood-2014-01-549360 [published Online First: 2014/05/08]
18. Miano M, Ramenghi U, Russo G, et al. Mycophenolate mofetil for the treatment of children with immune thrombocytopenia and Evans syndrome. A retrospective data review from the Italian association of paediatric haematology/oncology. *British journal of haematology* 2016;175(3):490-95. doi: 10.1111/bjh.14261 [published Online First: 2016/10/28]
19. Taylor A, Neave L, Solanki S, et al. Mycophenolate mofetil therapy for severe immune thrombocytopenia. *British journal of haematology* 2015;171(4):625-30. doi: 10.1111/bjh.13622 [published Online First: 2015/08/08]
20. Kotb R, Pinganaud C, Trichet C, et al. Efficacy of mycophenolate mofetil in adult refractory auto-immune cytopenias: a single center preliminary study. *European journal of haematology* 2005;75(1):60-4. doi: 10.1111/j.1600-0609.2005.00437.x [published Online First: 2005/06/11]
21. Provan D, Moss AJ, Newland AC, et al. Efficacy of mycophenolate mofetil as single-agent therapy for refractory immune thrombocytopenic purpura. *American journal of hematology* 2006;81(1):19-25. doi: 10.1002/ajh.20515 [published Online First: 2005/12/22]
22. Hou M, Peng J, Shi Y, et al. Mycophenolate mofetil (MMF) for the treatment of steroid-resistant idiopathic thrombocytopenic purpura. *European journal of haematology* 2003;70(6):353-7. [published Online First: 2003/05/21]
23. Colovic M, Suvajdzic N, Colovic N, et al. Mycophenolate mophetil therapy for chronic immune thrombocytopenic purpura resistant to steroids, immunosuppressants, and/or splenectomy in adults. *Platelets* 2011;22(2):153-6. doi: 10.3109/09537104.2010.520372 [published Online First: 2010/12/15]
24. Zhang WG, Ji L, Cao XM, et al. Mycophenolate mofetil as a treatment for refractory idiopathic thrombocytopenic purpura. *Acta pharmacologica Sinica* 2005;26(5):598-602. doi: 10.1111/j.1745-7254.2005.00088.x [published Online First: 2005/04/22]
25. Howard J, Hoffbrand AV, Prentice HG, et al. Mycophenolate mofetil for the treatment of refractory auto-immune haemolytic anaemia and auto-immune thrombocytopenia purpura. *British journal of haematology* 2002;117(3):712-5. [published Online First: 2002/05/25]

25. Newland A et al, poster at the British Society of Haematology meeting 2015

For peer review only - http://bmjopen.bmj.com/site/about/guidelines.xhtml

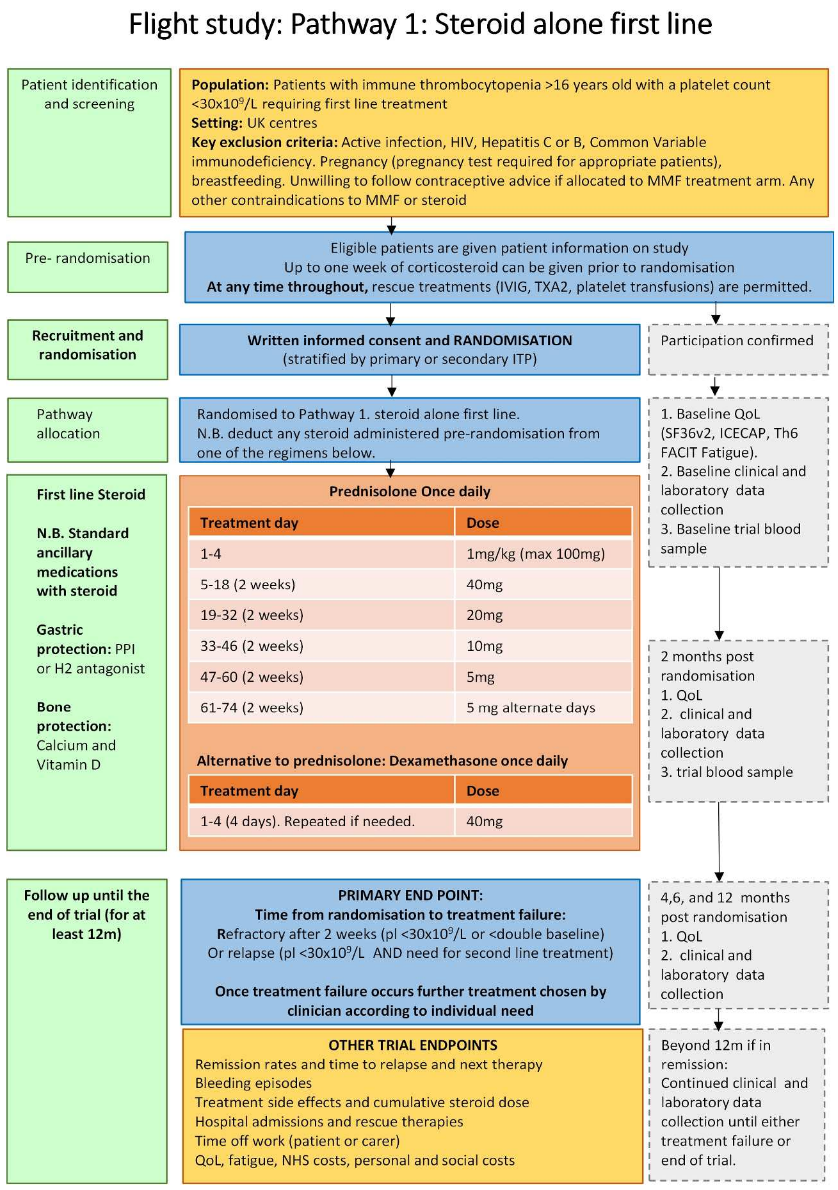

Figure 1. FLIGHT treatment pathway: Corticosteroid only

182x263mm (300 x 300 DPI)

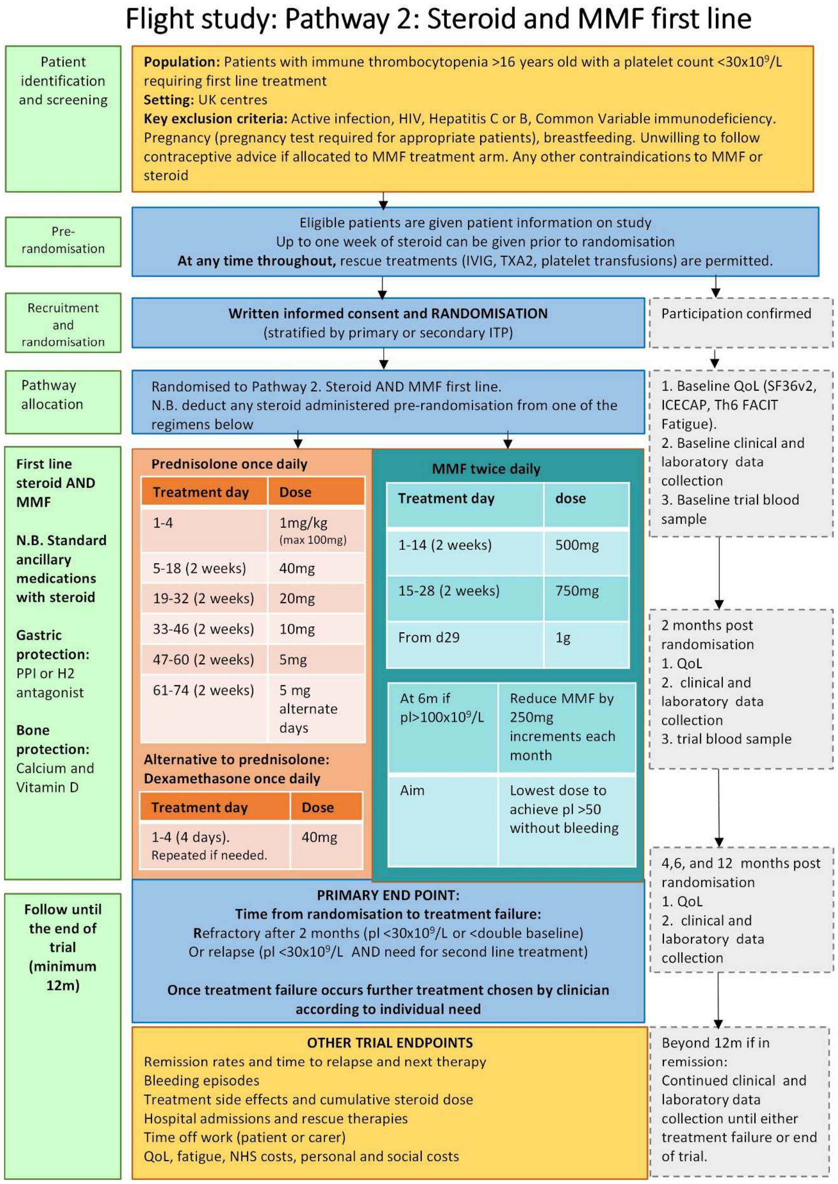

Figure 2. FLIGHT treatment pathway: Corticosteroid and Mycophenolate

185x267mm (300 x 300 DPI)

Reporting checklist for protocol of a clinical trial.

Based on the SPIRIT guidelines.

Instructions to authors

Complete this checklist by entering the page numbers from your manuscript where readers will find each of the items listed below.

Your article may not currently address all the items on the checklist. Please modify your text to include the missing information. If you are certain that an item does not apply, please write "n/a" and provide a short explanation.

Upload your completed checklist as an extra file when you submit to a journal.

In your methods section, say that you used the SPIRIT reporting guidelines, and cite them as:

Chan A-W, Tetzlaff JM, Altman DG, Laupacis A, Gøtzsche PC, Krleža-Jerić K, Hróbjartsson A, Mann H, Dickersin K, Berlin J, Doré C, Parulekar W, Summerskill W, Groves T, Schulz K, Sox H, Rockhold FW, Rennie D, Moher D. SPIRIT 2013 Statement: Defining standard protocol items for clinical trials. Ann Intern Med. 2013;158(3):200-207

		Reporting Item	Page Number
Title	#1	Descriptive title identifying the study design, population, interventions, and, if applicable, trial acronym	1
Trial registration	#2a	Trial identifier and registry name. If not yet registered, name of intended registry	2
Trial registration: data set	#2b	All items from the World Health Organization Trial Registration Data Set	2
Protocol version	#3	Date and version identifier	1
Funding	#4	Sources and types of financial, material, and other support	3
Roles and responsibilities: contributorship	#5a	Names, affiliations, and roles of protocol contributors	1 and 13
Roles and	#5b	Name and contact information for the trial sponsor	1

For peer review only

responsibilities: sponsor contact information			
Roles and responsibilities: sponsor and funder	#5c	Role of study sponsor and funders, if any, in study design; collection, management, analysis, and interpretation of data; writing of the report; and the decision to submit the report for publication, including whether they will have ultimate authority over any of these activities	Supplementary
Roles and responsibilities: committees	#5d	Composition, roles, and responsibilities of the coordinating centre, steering committee, endpoint adjudication committee, data management team, and other individuals or groups overseeing the trial, if applicable (see Item 21a for data monitoring committee)	Supplementary
Background and rationale	#6a	Description of research question and justification for undertaking the trial, including summary of relevant studies (published and unpublished) examining benefits and harms for each intervention	3-6
Background and rationale: choice of comparators	#6b	Explanation for choice of comparators	3-6
Objectives	#7	Specific objectives or hypotheses	6-7
Trial design	#8	Description of trial design including type of trial (eg, parallel group, crossover, factorial, single group), allocation ratio, and framework (eg, superiority, equivalence, non-inferiority, exploratory)	7
Study setting	#9	Description of study settings (eg, community clinic, academic hospital) and list of countries where data will be collected. Reference to where list of study sites can be obtained	8
Eligibility criteria	#10	Inclusion and exclusion criteria for participants. If applicable, eligibility criteria for study centres and individuals who will perform the interventions (eg, surgeons, psychotherapists)	7

For peer review only

Interventions: description	#11a	Interventions for each group with sufficient detail to allow replication, including how and when they will be administered	8, 9 and figure 1
Interventions: modifications	#11b	Criteria for discontinuing or modifying allocated interventions for a given trial participant (eg, drug dose change in response to harms, participant request, or improving / worsening disease)	Supplementary
Interventions: adherance	#11c	Strategies to improve adherence to intervention protocols, and any procedures for monitoring adherence (eg, drug tablet return; laboratory tests)	Supplementary
Interventions: concomitant care	#11d	Relevant concomitant care and interventions that are permitted or prohibited during the trial	8,9 and figure 1
Outcomes	#12	Primary, secondary, and other outcomes, including the specific measurement variable (eg, systolic blood pressure), analysis metric (eg, change from baseline, final value, time to event), method of aggregation (eg, median, proportion), and time point for each outcome. Explanation of the clinical relevance of chosen efficacy and harm outcomes is strongly recommended	9,10
Participant timeline	#13	Time schedule of enrolment, interventions (including any run-ins and washouts), assessments, and visits for participants. A schematic diagram is highly recommended (see Figure)	Table 1
Sample size	#14	Estimated number of participants needed to achieve study objectives and how it was determined, including clinical and statistical assumptions supporting any sample size calculations	11
Recruitment	#15	Strategies for achieving adequate participant enrolment to reach target sample size	11
Allocation: sequence generation	#16a	Method of generating the allocation sequence (eg, computer-generated random numbers), and list of any factors for stratification. To reduce predictability of a random sequence, details of any planned restriction (eg, blocking) should be provided in a	8

For peer review only

		separate document that is unavailable to those who enrol participants or assign interventions	
Allocation concealment mechanism	#16b	Mechanism of implementing the allocation sequence (eg, central telephone; sequentially numbered, opaque, sealed envelopes), describing any steps to conceal the sequence until interventions are assigned	8
Allocation: implementation	#16c	Who will generate the allocation sequence, who will enrol participants, and who will assign participants to interventions	8
Blinding (masking)	#17a	Who will be blinded after assignment to interventions (eg, trial participants, care providers, outcome assessors, data analysts), and how	N/A
Blinding (masking): emergency unblinding	#17b	If blinded, circumstances under which unblinding is permissible, and procedure for revealing a participant's allocated intervention during the trial	N/A
Data collection plan	#18a	Plans for assessment and collection of outcome, baseline, and other trial data, including any related processes to promote data quality (eg, duplicate measurements, training of assessors) and a description of study instruments (eg, questionnaires, laboratory tests) along with their reliability and validity, if known. Reference to where data collection forms can be found, if not in the protocol	10 and table 1
Data collection plan: retention	#18b	Plans to promote participant retention and complete follow-up, including list of any outcome data to be collected for participants who discontinue or deviate from intervention protocols	Supplementary
Data management	#19	Plans for data entry, coding, security, and storage, including any related processes to promote data quality (eg, double data entry; range checks for data values). Reference to where details of data management procedures can be found, if not in the protocol	10 and Supplementary
Statistics: outcomes	#20a	Statistical methods for analysing primary and secondary outcomes. Reference to where other	11,12

For peer review only

		details of the statistical analysis plan can be found, if not in the protocol	
Statistics: additional analyses	#20b	Methods for any additional analyses (eg, subgroup and adjusted analyses)	NA
Statistics: analysis population and missing data	#20c	Definition of analysis population relating to protocol non-adherence (eg, as randomised analysis), and any statistical methods to handle missing data (eg, multiple imputation)	Supplementary
Data monitoring: formal committee	#21a	Composition of data monitoring committee (DMC); summary of its role and reporting structure; statement of whether it is independent from the sponsor and competing interests; and reference to where further details about its charter can be found, if not in the protocol. Alternatively, an explanation of why a DMC is not needed	Supplementary
Data monitoring: interim analysis	#21b	Description of any interim analyses and stopping guidelines, including who will have access to these interim results and make the final decision to terminate the trial	12
Harms	#22	Plans for collecting, assessing, reporting, and managing solicited and spontaneously reported adverse events and other unintended effects of trial interventions or trial conduct	Supplementary
Auditing	#23	Frequency and procedures for auditing trial conduct, if any, and whether the process will be independent from investigators and the sponsor	Supplementary
Research ethics approval	#24	Plans for seeking research ethics committee / institutional review board (REC / IRB) approval	2
Protocol amendments	#25	Plans for communicating important protocol modifications (eg, changes to eligibility criteria, outcomes, analyses) to relevant parties (eg, investigators, REC / IRBs, trial participants, trial registries, journals, regulators)	Supplementary
Consent or assent	#26a	Who will obtain informed consent or assent from potential trial participants or authorised surrogates,	7 and Supplementary

For peer review only

		and how (see Item 32)	
Consent or assent: ancillary studies	#26b	Additional consent provisions for collection and use of participant data and biological specimens in ancillary studies, if applicable	10 and Supplementary
Confidentiality	#27	How personal information about potential and enrolled participants will be collected, shared, and maintained in order to protect confidentiality before, during, and after the trial	Supplementary
Declaration of interests	#28	Financial and other competing interests for principal investigators for the overall trial and each study site	3
Data access	#29	Statement of who will have access to the final trial dataset, and disclosure of contractual agreements that limit such access for investigators	Supplementary
Ancillary and post trial care	#30	Provisions, if any, for ancillary and post-trial care, and for compensation to those who suffer harm from trial participation	Supplementary
Dissemination policy: trial results	#31a	Plans for investigators and sponsor to communicate trial results to participants, healthcare professionals, the public, and other relevant groups (eg, via publication, reporting in results databases, or other data sharing arrangements), including any publication restrictions	2
Dissemination policy: authorship	#31b	Authorship eligibility guidelines and any intended use of professional writers	NA
Dissemination policy: reproducible research	#31c	Plans, if any, for granting public access to the full protocol, participant-level dataset, and statistical code	Supplementary
Informed consent materials	#32	Model consent form and other related documentation given to participants and authorised surrogates	Supplementary
Biological specimens	#33	Plans for collection, laboratory evaluation, and storage of biological specimens for genetic or molecular analysis in the current trial and for future use in ancillary studies, if applicable	Supplementary

For peer review only

The SPIRIT checklist is distributed under the terms of the Creative Commons Attribution License CC-BY-ND 3.0. This checklist can be completed online using https://www.goodreports.org/, a tool made by the EQUATOR Network in collaboration with Penelope.ai

For peer review only

BMJ Open

Trial Protocol: A multicentre randomised trial of First Line treatment pathways for newly diagnosed Immune Thrombocytopenia: Standard steroid treatment versus combined steroid and mycophenolate. The FLIGHT trial

Journal:	*BMJ Open*
Manuscript ID	bmjopen-2018-024427.R2
Article Type:	Protocol
Date Submitted by the Author:	24-Sep-2018
Complete List of Authors:	Pell, Julie; Cardiff University Centre for Trials Research<br>Greenwood, Rosemary; University Hospitals Bristol NHS Foundation Trust, Research and Design Service<br>Ingram, Jenny; Bristol University , School of Social & Community Medicine<br>Wale, Katharine; Research and Innovation, University Hospitals Bristol<br>Thomas, Ian; Cardiff University Centre for Trials Research<br>Kandiyali, Rebecca; Research and Innovation, University Hospitals Bristol<br>Mumford, Andrew; University Hospitals Bristol NHS Foundation Trust, Department of Haematology<br>Dick, Andrew; University of Bristol<br>Bagot, Catherine; Glasgow Royal Infirmary<br>Cooper, Nichola; Imperial College London<br>Hill, Quentin; Leeds Teaching Hospitals NHS Trust<br>Bradbury, Charlotte; University of Bristol, Cellular and Molecular Medicine; University Hospitals Bristol NHS Foundation Trust, Haematology
**Primary Subject Heading**:	Haematology (incl blood transfusion)
Secondary Subject Heading:	Immunology (including allergy)
Keywords:	Immune Thrombocytopenia, ITP, Prednisolone, mycophenolate, Dexamethasone, corticosteroid

SCHOLARONE™
Manuscripts

TITLE: Trial Protocol: A multicentre randomised trial of First Line treatment pathways for newly diagnosed Immune Thrombocytopenia: Standard steroid treatment versus combined steroid and mycophenolate. The FLIGHT trial

Authors: Julie Pell[1], Rosemary Greenwood[2], Jenny Ingram[2], Katherine Wale[3], Ian Thomas[1], Rebecca Kandiyali[3], Andrew Mumford[4,5], Andrew Dick[4,6], Catherine Bagot[7], Nichola Cooper[8], Quentin Hill[9], Charlotte Bradbury[4,5]

1 Centre for Trials Research, Cardiff University, PellJC@cardiff.ac.uk, thomasif@cardiff.ac.uk,
2 Research Design Service – South West, University of Bristol, rosemary.greenwood@UHBristol.nhs.uk, jenny.ingram@bristol.ac.uk,
3 Research & Innovation, University Hospitals Bristol NHS Foundation Trust, katharine.Wale@UHBristol.nhs.uk, rebecca.kandiyali@uhbristol.nhs.uk,
4 Cellular and Molecular Medicine, University of Bristol, a.mumford@bristol.ac.uk, a.dick@bristol.ac.uk, c.bradbury@bristol.ac.uk,
5 Department of Haematology, University Hospitals Bristol NHS Foundation Trust
6 UCL-Institute of Ophthalmology
7 Glasgow Royal Infirmary, Glasgow, Catherine.Bagot@ggc.scot.nhs.uk,
8 Imperial College London and Hammersmith Hospital, London, n.cooper@imperial.ac.uk,
9 Leeds Teaching Hospitals NHS Trust, quentinhill@nhs.net,

KEYWORDS

Immune Thrombocytopenia, Prednisolone, Mycophenolate, Corticosteroid

Corresponding author: Dr Charlotte Bradbury, Level 8, Bristol Haematology & Oncology Centre, Horfield Road, Bristol, BS2 8ED c.bradbury@bristol.ac.uk

Version: V3.0, 24-Oct 2017

Trial sponsor: University Hospitals Bristol NHS Foundation Trust, Research and Innovation, Level 3, UH Bristol Education and Research Centre, Upper Maudlin Street, Bristol BS2 8AE

Word Count: 4058

For peer review only - http://bmjopen.bmj.com/site/about/guidelines.xhtml

ABSTRACT

Introduction

Immune thrombocytopenia (ITP) is an autoimmune condition that may cause thrombocytopenia related bleeding. Current first line ITP treatment is with high dose corticosteroids but frequent side effects, heterogeneous responses and high relapse rates are significant problems with only 20% remaining in sustained remission with this approach. Mycophenolate (MMF) is often used as the next treatment with efficacy in 50-80% of patients and good tolerability but can take up to 2 months to work.

Objective: To test the hypothesis that MMF combined with corticosteroid is a more effective first line treatment for immune thrombocytopenia (ITP) than current standard of corticosteroid alone.

Methods and analysis

Design: Multicentre, UK based, open label, randomised controlled trial

Setting: Haematology departments in secondary care

Participants: We plan to recruit 120 patients >16 years old with a diagnosis of ITP and a platelet count <30 $x10^9$/L who require first line treatment. Patients will be followed up for a minimum of 12 months following randomisation.

Primary outcome: Time from randomisation to treatment failure defined as platelets <$30x10^9$/L and a need for 2nd line treatment.

Secondary outcomes: Side effects, bleeding events, remission rates, time to relapse, time to next therapy, cumulative corticosteroid dose, rescue therapy, splenectomy, socioeconomic costs, patient reported outcomes (quality of life, fatigue, impact of bleeding, care costs).

Analysis: The sample size of 120 achieves a 91.5% power to detect a doubling of the median time to treatment failure from 5 to 10 months. This will be expressed as a hazard ratio with 95% confidence interval, median time to event if more than 50% have had an event and illustrated with Kaplan Meier curves. Cost effectiveness will be based on the first 12 months from diagnosis.

Ethics and dissemination

Ethical approval from NRES Committee South West (IRAS number 225959). EudraCT Number: 2017-001171-23. ClinicalTrials.gov number: NCT03156452. Results will be submitted for publication in peer-reviewed journals.

For peer review only - http://bmjopen.bmj.com/site/about/guidelines.xhtml

STRENGTHS AND LIMITATIONS OF THIS STUDY

- First UK multicentre RCT for first line treatment of ITP
- Tests a pragmatic, cost effective approach which if effective, may be applicable to other autoimmune conditions
- The trial includes patient-oriented outcomes by using validated questionnaires to assess quality of life, fatigue, impact of bleeding and care costs
- Option to consent to additional blood samples for translational research to maximise scientific potential
- The limitations include the open label design, lack of very long term follow up and sample size unable to detect small differences between treatment arms

FUNDING

The FLIGHT trial is independent research funded by the National Institute for Health Research (NIHR) under its Research for Patient Benefit (RfPB) Programme (Grant Reference Number PB-PG-0815-20016). The views expressed are those of the authors and not necessarily those of the NHS, the NIHR or the Department of Health.

CONFLICTS OF INTEREST

None of the authors have competing interests

INTRODUCTION

Immune thrombocytopenia (ITP) has an incidence of 2.9/100,000 person-years.[1] It is an autoimmune condition that may present with bleeding and bruising due to a low platelet count. In ITP, there is increased consumption and reduced production of platelets due to both antibody and cell mediated autoimmune attack of platelets and megakaryocytes involving dysregulated autoreactive T and B cell lymphocytes.[2-5]

ITP can be classified according to the duration of illness into newly diagnosed (<3 months), persistent (3-12 months) and chronic (>12 months).[6] ITP may also be classified as either primary when it presents in isolation or secondary when ITP occurs in the context of an associated illness or medication.[6]

For peer review only - http://bmjopen.bmj.com/site/about/guidelines.xhtml

ITP is a diagnosis of exclusion and made when the platelet count <100x 109/L and other causes of thrombocytopenia are excluded by history, examination and laboratory evaluation.[6,7]

Current first line ITP treatment is with high dose corticosteroids but this has several downsides. First, the majority of patients suffer significant side effects including mood swings, difficulty sleeping, weight gain, high blood pressure, diabetes, gastric irritation, skin thinning and osteoporosis. A published survey of ITP patients reported 98% had at least one side effect and 38% stopped or reduced dosage due to intolerable side effects.[8] In the UK ITP registry, the most frequently reported co-morbidities were related to corticosteroids and correlated with duration of treatment (hypertension in 30%, diabetes in 19%).[9] The second problem is that patients are heterogeneous in their response to corticosteroid with some (approximately 20%) not responding at all and the majority of others (70%-90%) relapsing when the corticosteroids are reduced or stopped.[7,10,11] Patients who are refractory or relapse (the majority), remain at risk of bleeding/bruising, which occasionally can be severe including intracranial haemorrhage.[12] They often receive more corticosteroid with associated side effects. Some require hospital admission and expensive rescue therapies (e.g. IVIG for a 70kg patient=£3,906). They continue to require frequent blood tests and doctor visits and are usually unable to continue their normal activities until their illness is controlled. Fatigue is also associated with disease activity and can be severe.[13] Physical factors combine with psychological stress through fear of bleeding, need for time off work and lifestyle restrictions due to bleeding risk to adversely impact quality of life.[14,15]

First line treatment for ITP is unsatisfactory but it remains unchanged for decades. Although a small number of studies have tested alternative approaches, a well- tolerated, effective and durable new approach has not been conclusively demonstrated. High dose corticosteroid remains the standard first line treatment recommended in most countries.[10]

Compared to cancers in haematology, ITP remains relatively under-researched. The few trials done in ITP have often been funded by pharmaceutical companies, risking publication bias towards high cost non-generic drugs. For example, many "cheap", generic drugs commonly prescribed for ITP such as azathioprine, mycophenolate and dapsone, have never been tested in randomised controlled trials in ITP. In contrast, the more expensive treatments, TPO-RAs and Rituximab have been tested in well- designed adequately powered RCTs. The relative rarity (2.9/100,000 person-years), non-cancerous nature and rare impact on survival of ITP have prevented ITP being a priority for research funding in the past. However, this underestimates the profound adverse impact a

For peer review only - http://bmjopen.bmj.com/site/about/guidelines.xhtml

diagnosis of ITP and its treatment can have for individual patients, many of whom are young. There is also a costly financial impact for the NHS from the healthcare resources patients require when their illness is uncontrolled. In addition, the problems faced by patients with ITP mirror those with other autoimmune conditions which as a group are common, affecting 3% of the population. There is an urgent clinical need to address this inequality, improving first line treatment for ITP through high quality, independently funded research to allow patients with this condition access to improvements in care seen in other illnesses such as cancer or heart disease.

Current popular options for second line or subsequent treatment include Mycophenolate, Rituximab, Thrombopoeitin receptor agonists (TPO RA) and splenectomy. Splenectomy is an effective treatment (60% long term remission rates) but irreversible and international guidelines recommend deferring splenectomy for the first 12 months following diagnosis due to the chance of spontaneous remission (risk of unnecessarily removing a healthy organ).[7,11] Surgical operations are not popular with patients and there is increasing awareness of the short and long term complications of splenectomy including infection, bleeding, arterial and venous thrombosis, cancer and relapse.[16] The splenectomy numbers performed in the UK has dramatically reduced over recent years (UK ITP registry data). Rituximab is a monoclonal antibody treatment which targets antibody production by B cells. It is relatively expensive, with disappointing long term remission rates similar to placebo.[16] TPO RA stimulate platelet production, are well tolerated and effective in the majority[17] but at significant financial cost, prohibiting widespread use in the UK for early treatment (NICE guidance). A small (n=12) non-randomised study using TPO RA with corticosteroid first line showed efficacy but perhaps less than expected.[18] By contrast, Mycophenolate (MMF) is a widely used second line agent in the UK due to good efficacy (response rates of 50-80%), safety and tolerability profile.[19-26] Mycophenolate has activity against both autoreactive T and B cells and has also shown efficacy in refractory ITP including steroid resistance suggesting a complimentary mechanism of action. [24] It is less expensive to the NHS than some other second line options costing approximately £182/year (generic cost) compared to costs for average doses of romiplostim (TPO RA) at £25 000/year, Eltrombopag (TPO RA) £20 000/year or rituximab at £8000 for a course of 4 infusions (375mg/m2 each dose) or £1000 (100mg each dose). However, similar to other second line therapies, MMF has a relatively slow (up to 2 months) onset of action. In the meantime, patients often receive further steroid (to maintain a "safe platelet count") and continue to suffer problems associated with their illness (see above). Direct feedback from patients regarding the difficulties they face in the first months following ITP diagnosis has been the primary driving force for this

For peer review only - http://bmjopen.bmj.com/site/about/guidelines.xhtml

clinical trial. Local (Bristol) and national patient groups (ITP support association) have been fundamental to the formulation of patient relevant priorities for treatment.

Rationale

The Flight trial is the first UK, NHS coordinated, Pharma independent multicentre randomised controlled trial, testing a "common sense/practical" new approach using MMF first line instead of second line with the aim of preventing the almost inevitable first relapse when corticosteroids stop. Patients will be randomly allocated to one of 2 treatment arms, either standard of care (corticosteroid alone) or MMF combined with corticosteroid with the primary outcome of time to treatment failure. By giving patients a stable platelet count sooner, we expect to improve other outcomes such as quality of life and fatigue. By reducing the risk of relapse, patients may also be less likely to receive a second course of corticosteroid with associated side effects. Potential indirect benefits to the NHS include reduced need for rescue treatments, blood tests, hospital attendances and admissions and reduced need for high cost treatments such as TPO mimetics. However, there will be some patients who will be treated with MMF who may have been successfully treated with corticosteroids alone (10-30%).[7, 9, 10] Similar to other immunosuppressives, MMF may slightly increase infection and cancer risk with long term use (SmPC) In addition, MMF is teratogenic and therefore stringent pregnancy prevention is essential for men and women taking the drug. This puts the trial in equipoise. The trial includes a strategy to reduce and stop MMF at 6 months for patients in complete remission to prevent unnecessary long-term use.

The choice of this open label design was made in order to allow true patient treatment costs to be calculated for the cost effectiveness analysis, and to deal with the complexities of placebo controlling a drug that needed titrating at the start and tapering at the end. In addition, over encapsulation was only possible for the lower MMF dose (250 mg) and the resulting capsule was the largest size which would mean most patients taking 8 large capsules per day in both arms; something that patients in Bristol thought would put them off taking part in the study. Patients were clear that from their perspective that a straight forward open label design would be preferable and was easier for a new patient to understand and consent. In addition, the quotes from 2 separate companies also showed the financial costs of encapsulation to generate a placebo were prohibitively expensive.

This trial proposal has received support and input from clinicians and patients nationally (UK ITP forum and ITP support association). To ensure objective and meaningful outcomes, it will be a

For peer review only - http://bmjopen.bmj.com/site/about/guidelines.xhtml

multicentre RCT, aiming to recruit 120 patients (expecting 100 full datasets). Patients will be given up to one week of corticosteroid prior to randomisation to enable sufficient time to read information, discuss and ask questions with informed consent in an appropriate setting. Patients will receive the usual follow up according to clinical need and local policy. Laboratory and clinical data will be collected from routine appointments. In addition, patient-oriented outcomes will be recorded at diagnosis, 2, 4, 6, and 12 months using validated patient questionnaires. Patients are also offered consent to additional blood samples for translational research studies (time 0 and 2 months).

Primary objective

To compare two first line treatment pathways for ITP, standard corticosteroid only versus corticosteroid combined with MMF and demonstrate which pathway helps patients achieve a stable platelet count sooner, measured as survival free from treatment failure (time from randomisation to treatment failure).

METHODS AND ANALYSIS

Trial design

A multicentre, open label randomised clinical trial of MMF with corticosteroid as first line treatment for patients with ITP versus the standard care pathway of corticosteroids alone as first line treatment.

Eligibility criteria

Inclusion criteria: Patients >16 years old with a diagnosis of ITP (primary or secondary), a platelet count <$30x10^9$/L AND a clinical need for first line treatment. Patients have provided written informed consent.

Patients can be recruited at any time after ITP diagnosis if they are suitable for first line treatment (i.e. Not previously or recently treated). Patients can receive up to 1 week of corticosteroid prior to recruitment to allow time to be informed about the trial, with the opportunity to ask questions and for consent to be taken during a routine specialist clinic appointment if preferred.

Exclusion criteria: Pregnancy and breastfeeding (Women of child bearing potential require a pregnancy test result within 7 days prior to randomisation to rule out unintended pregnancy). Patients with HIV, Hepatitis B or C, or Common Variable immunodeficiency. Contraindications to

For peer review only - http://bmjopen.bmj.com/site/about/guidelines.xhtml

For peer review only

MMF or corticosteroid (see SmPC) including patients with active significant infections, hypersensitivity to mycophenolate mofetil, mycophenolic acid or to any of the excipients or active significant infection. Patients not capable of giving informed consent (e.g. due to incapacity). Patients (men and women) unwilling to follow contraceptive advice if allocated to MMF treatment arm.

Study setting

120 patients will be recruited from approximately 40 Haematology departments of hospitals (secondary care) across the UK where ITP patients are treated.

The trial processes will be run by the Centre for Trials Research (CTR), Cardiff University and sponsored by University Hospitals Bristol NHS Foundation Trust.

The flight trial opened for recruitment on 26th October 2017.

Randomisation

Patients who agree to participate will be randomised to MMF with corticosteroid or corticosteroid alone in a 1:1 ratio using a secure web based randomisation system based at Cardiff CTR. Randomisation will be stratified by primary or secondary ITP diagnosis. Due to the large number of centres and the small number of patients it will not be sensible to stratify randomisation by study centre. However, to ensure an even spread of patients across time, randomisation will be blocked using random block sizes of 6 and 8 to retain concealment.

Treatment arms (Figures 1 and 2):

1. **Corticosteroid only pathway (Figure 1):** 1mg/kg od prednisolone 4 days (maximum of 100mg), 40mg od 2 weeks, 20mg od 2 weeks, 10mg od 2 weeks, 5mg od 2 weeks then 5mg alternate days 2 weeks then stop*. For the duration of steroid, patients will get a PPI or H2 antagonist to protect against gastric bleeding and appropriate bone protection.

*Dexamethasone 20mg or 40mg orally daily for 4 days is an alternative option to prednisolone if deemed clinically more appropriate for individual circumstances.

2. **Corticosteroid +MMF pathway (Figure 2):** 1mg/kg od prednisolone 4 days (maximum of 100mg), 40mg od 2 weeks, 20mg od 2 weeks, 10mg od 2 weeks, 5mg od 2 weeks then 5mg alternate days 2 weeks then stop*. For the duration of steroid, patients will get a PPI or H2 antagonist to protect against gastric bleeding and appropriate bone protection.

For peer review only - http://bmjopen.bmj.com/site/about/guidelines.xhtml

*Dexamethasone 20mg or 40mg daily for 4 days is an alternative option to prednisolone if deemed clinically more appropriate for individual circumstances.

From randomisation (alongside steroid), MMF 500mg bd starting dose then increased to 750mg bd after 2 weeks if tolerated (no side effects or laboratory concerns such as neutropenia) and 1g bd after another 2 weeks if tolerated (4 weeks after starting). Earlier dose escalation to MMF 1g bd can be considered if clinically indicated.

After 6 months of MMF therapy, all patients who have remained in complete remission (pl count> 100 x10 ^{9}/L) will reduce the dose by 250mg (one capsule) each month. The aim is to continue on the lowest dose that achieves a haemostatic (safe) platelet count (pl >30 x10 ^{9}/L) and to ensure that patients who have gone into a remission do not continue to take the drug indefinitely.

In both groups: Any steroid commenced prior to randomisation will be deducted from the regimes. Importantly, emergency and rescue treatments will be permitted throughout the study. These include platelet transfusions, tranexamic acid and intravenous immunoglobulin. These are known not to impact on the natural history of ITP and it is recognised that they may be important for patient safety. The use of "rescue treatments" will be recorded on the CRF.

In addition, some degree of flexibility of corticosteroid dose and duration may be needed for individual patients according to comorbidity, tolerability and other factors.

If treatment failure occurs, choice of second line treatment will be individualised according to patient's clinical circumstances. Further steroid will be given according to clinical need.

Primary outcome is time from randomisation to treatment failure defined as a pl count <30x10^{9}/L AND a need to commence second line treatment. This will include patients who are refractory (pl <30x10^{9}/L in spite of 2 weeks treatment in the steroid arm or pl <30x10^{9}/L in spite of 2 months treatment in the steroid +MMF arm) or who initially respond but then relapse (defined clinically as pl <30x10^{9}/L and need for further therapy). Patients with a clinical need to start 2nd line treatment early (within 2 weeks for the steroid only arm and within 2 months for the MMF and steroid arm), for example due to significant bleeding, will also be classed as treatment failures.

Secondary outcomes

For peer review only - http://bmjopen.bmj.com/site/about/guidelines.xhtml

1. Medication side effects, toxicity or other adverse events (including infection episodes)
2. Bleeding events
    a. Site and type of bleeding
    b. Treatment required for bleeding
    c. Whether hospital admission was required
    d. Whether ITP rescue treatments were needed
3. Remission rates (pl >30 x10^9/L and at least 2 fold increase from baseline); Complete 100x10^9/L, partial 30-100x10^9/L.
4. Time to relapse and time to next therapy
5. Cumulative corticosteroid dose
6. Need for rescue therapies
7. Need for splenectomy
8. Socioeconomic costs
9. Patient reported outcomes (quality of life, fatigue, impact of bleeding, care costs).

Patients follow up

Patients will be followed up until the end of the trial and for a minimum of 12 months. They will receive the usual follow up according to clinical need and local policy. Laboratory and clinical data will be collected from routine appointments. In addition, patient oriented outcomes and additional data will be recorded at diagnosis, 2, 4, 6, and 12 months using validated patient questionnaires. Patients are also offered consent to additional blood samples for translational research studies (time 0 and 2 months).

Data collection (Table 1)

Hospital monitoring of platelet levels (FBC) is part of routine care for ITP patients and these data will be collected and recorded on the CRF without requiring patients to come in for additional samples to be taken. These locally collected samples may be collected monthly (or less often) for patients believed to be in stable remission and weekly at lower or declining platelet levels. We expect this to allow us to calculate the time in remission and time to relapse with reasonable accuracy over the 12 to 24 month follow up period. Other clinical and laboratory data needed for the trial endpoints will be collected from the medical and electronic records and recorded on the CRF. In addition, we will also ask the patients to complete questionnaires on fatigue, quality of life and bleeding scores at baseline, 2, 4, 6, and 12 months (Table 1).

Patient reported outcomes will be captured by the following questionnaires:

For peer review only - http://bmjopen.bmj.com/site/about/guidelines.xhtml

1. SF36v2 (Your health & wellbeing) – Quality of life,
2. FACIT-Fatigue (version 4) – Fatigue,
3. FACT-Th6 (version 4)- Bleeding,
4. ICECAP-A V2- Quality of life,
5. Health economic/resource use questionnaire - Personal and social costs.

Additional optional research blood samples (requiring separate consent) will be sent at baseline and 2 month follow up to the Bristol Biobank.

Data Management

Source documents produced for this trial will be kept in the patient's hospital records and source data will be transcribed into trial-specific Case Report Forms (CRFs) at the end of each patient visit. Data recording for this trial will be via a web based system. This is a secure encrypted system accessed by an institutional password which complies with Data Protection Act standards. The database will be stored and regularly backed up on a Cardiff University Server. The CRFs will be coded with the study number and will not include patients' names and addresses

Patient and Public Involvement and Engagement

During the trial development, a group of 8 ITP patients discussed the study design, burden of outcome measure completion to patients and the size of a potential placebo capsule which they reported could put them off getting involved in a trial. They reported that avoidance of relapse, early achievement of a stable platelet count, reduced overall corticosteroid dose and reduced hospital attendances are the most important goals for ITP management from their perspective.

We formed a Patient Advisory Group (PAG) with some of these patients and representatives from the ITP association that will advise the trial management group throughout the study. They have commented on all patient-facing documentation and will be instrumental in disseminating the study findings to patient groups and the public.

STATISTICS AND DATA ANALYSIS

Sample size calculation

There is no published clinical data available for MMF use first line in ITP as this is a novel approach. We have analysed local data on MMF used second line in ITP in 12 patients which shows an estimated median survival free from treatment failure of more than 10 months. We have data on 68 who experienced corticosteroids as a first line treatment showing that 70% of them had experienced

For peer review only - http://bmjopen.bmj.com/site/about/guidelines.xhtml

a treatment failure by 12 months and that the median survival free from treatment failure was 5.0 months (95% CI [3.2, 6.8]). Data for the 12 patients treated with MMF second line therapy have shorter follow-up times, with only 5 patients having follow up beyond 12 months. The cox proportional hazards ratio model demonstrates the 90% confidence interval for the hazard ratio to be between 0.13 and 0.59, showing that our decision to power this on an estimate of a hazard of lower than 0.5 is potentially achievable.

Clinically a doubling in the time to remission was thought to be something that the patients would have welcomed. Less than that was not thought to be sufficient grounds for switching this treatment from second line to first line due to the potential for additional toxicity and immune suppression in those who may have remained in remission with corticosteroids alone.

The sample size of 120 (60 per group) with less than 5% loss to follow-up achieves 91.5% power to detect a doubling of the median time to treatment failure from 5 months to 10 months if the patients are recruited at a steady rate or 10 per month for 12 months and all followed up until the last patients reaches 12 months follow up.

Statistical analysis

The full statistical analysis will be written into a statistical analysis plan available separately. The analysis will produce a CONSORT diagram for the reporting of clinical trials.

The baseline characteristics of the two groups will be tabulated but not tested for statistically significant differences between the groups.

The primary analysis is by intention to treat. However an investigation of compliance with the treatment pathway and compliance with the criteria for changing to a second line therapy will be carried out prior to the primary analysis to check the date of the primary event. The primary event is the date at which there was a requirement for second line therapy. Where the platelet count falls below the level required for this treatment decision, the first date at which either symptoms or a blood test revealing this event will be used. If a clinician decides to use a second line therapy without a platelet count below the criteria, the date of the treatment decision/new prescription will be taken to represent that event. The results will be expressed as a hazard ratio with 95% confidence interval, median time to event if more than 50% have had an event and plotted as Kaplan Meier curves.

For peer review only - http://bmjopen.bmj.com/site/about/guidelines.xhtml

The primary analysis will contain all patients who are randomised for as long as they have been followed up or until their first event in a survival analysis using intention to treat methodology. All patients will be followed up to 12 months. In addition, patients who have not had an event in the first 12 months post randomisation will be followed until their first event or until the last patient has reached the 12 month point – whichever is the sooner and included in the analysis until that time accordingly. Sensitivity analyses will include landmark analysis or shifting the time line to classify all treatment failures before 2 months as at 2 months in order to prevent potential biases caused by different definitions of treatment failure time frames between the two groups.

Analysis of other outcomes will use as full a data set as possible and focus on the 12 month data point or area under the curve as appropriate and detailed in the analysis plan.

No interim analyses of the main endpoint will be supplied to the independent Data Monitoring Committee (DMC) due to the short time frame (12 months recruitment) in which all patients will be recruited by the time the first patient has completed follow-up. Serious adverse event rates will be reported on a monthly basis to the trial management group (TMG) and the DMC. The DMC could advise the chairman of the Trial Steering Committee and Chief Investigator if these provide proof beyond reasonable doubt that it would be unethical to continue with the trial.

Pharmacovigilance

The collection and reporting of all adverse events is in accordance with the Medicines for Human Use Clinical Trials Regulations 2004 and its subsequent amendments and follows the standard operating procedures of the trials unit, Cardiff University Centre for Trials Research (CTR). Seriousness and causality are assessed by participating sites and further review of expectedness (based on the reference safety information) is conducted centrally on behalf of the Sponsor. Events are defined as serious adverse events (SAEs), serious adverse reactions (SARs) or suspected unexpected serious adverse reaction (SUSARs) in line with regulatory definitions on the basis of these assessments.

SAEs are reported throughout the treatment period up to 6 weeks after cessation of last dose of MMF. SARs should continue to be reported until the end of follow up. All deaths and overdoses are reported to the Sponsor as an SAE and reviewed in line with other events.

For peer review only - http://bmjopen.bmj.com/site/about/guidelines.xhtml

MMF in this trial has a genotoxic and teratogenic potential and therefore pregnancy is contraindicated. Participants that are female of child bearing potential, or male with female partners of equal potential, are required to use contraception as indicated in the protocol. Pregnancy, or the pregnancy of a partner occurring whilst participating in the trial, is not considered an SAE, however, a congenital anomaly or birth defect is. Pregnancy is reported to Sponsor and followed up to outcome.

All safety events are reviewed by the Trial Management Group on an ongoing basis and reported to the Trial Steering Committee for oversight. Overall assessment of the safety profile of both arms will be included in final reporting and publication.

Author Contributions: Charlotte Bradbury is chief investigator for the Flight trial and was responsible for writing the protocol with clinical input from Nichola Cooper, Quentin Hill and Catherine Bagot. Rosemary Greenwood is the trial statistician who has contributed to the trial design and writing of protocol. Jenny Ingram contributed to trial design and leads the Patient Advisory Group input. Rebecca Kandiyali is the trial health economist and provided input to the trial design and protocol. Julie Pell and Ian Thomas (Cardiff CTR) and Katharine Wale (sponsor representative) have also provided contribution to writing the protocol. Andrew Mumford and Andrew Dick have provided mentorship to the Chief investigator.

Acknowledgements: We would like to thank the members of the UK ITP forum who have been invaluable providing feedback on the trial design and recruitment to the trial.

Figure 1. Flight treatment pathway: Corticosteroid only

Figure 2. Flight treatment pathway: Corticosteroid and Mycophenolate

For peer review only

For peer review only - http://bmjopen.bmj.com/site/about/guidelines.xhtml

Table 1: Time schedule of enrolment, interventions, assessments and visits

Procedures	V0	V1	V2 (2 mths)	V3 (4 mths)	V4 (6 mths)	V5 (12 mths)	V6 12-24 mths
	Screen	Baseline / Randomisation to pathway 1 or 2	Follow up	Follow up	Follow up	Follow up	Data collection from sites
Eligibility assessment	x						
Randomisation		x					
Informed consent		x					
Demographics		x					
Medical history		x	x	x	x	x	
Physical examination		x					
Vital signs (incl height & weight)		x	x	x	x	x	
Pregnancy test	x						
Concomitant medications		x	x	x	x	x	
Standard practice bloods (includes blood sugar if applicable)	x	x	x	x	x	x	
Hepatitis B, C & HIV serology	x						
Immunoglobulins (blood)		x			x	x	
Extra blood samples (optional)		x	x				
Dispensing of trial drugs		x*					
Compliance			x				
QofL FACT-Th6, V4	x	x	x	x	x	x	
QofL ICECAP V2 – A measure	x	x	x	x	x	x	
QofL SF-36v2 – Health Survey	x	x	x	x	x	x	
QofL FACIT-F, V4, pg 3 (fatigue)	x	x	x	x	x	x	
QofL Thrombocytopenia costs questionnaire	x	x	x	x	x	x	
Data collection from sites on platelet count & treatment		x	x	x	x	x	x

* MMF and corticosteroid dispensing frequency can follow standard local practice.

For peer review only

For peer review only - http://bmjopen.bmj.com/site/about/guidelines.xhtml

REFERENCES

1. Moulis G, Palmaro A, Montastruc JL, et al. Epidemiology of incident immune thrombocytopenia: a nationwide population-based study in France. Blood 2014;124(22):3308-15. doi: 10.1182/blood-2014-05-578336 [published Online First: 2014/10/12]

2. McKenzie CG, Guo L, Freedman J, et al. Cellular immune dysfunction in immune thrombocytopenia (ITP). British journal of haematology 2013;163(1):10-23. doi: 10.1111/bjh.12480 [published Online First: 2013/08/14]

3. Iraqi M, Perdomo J, Yan F, et al. Immune thrombocytopenia: antiplatelet autoantibodies inhibit proplatelet formation by megakaryocytes and impair platelet production in vitro. Haematologica 2015;100(5):623-32. doi: 10.3324/haematol.2014.115634 [published Online First: 2015/02/16]

4. Ji X, Zhang L, Peng J, et al. T cell immune abnormalities in immune thrombocytopenia. Journal of hematology & oncology 2014;7:72. doi: 10.1186/s13045-014-0072-6 [published Online First: 2014/10/03]

5. Audia S, Rossato M, Santegoets K, et al. Splenic TFH expansion participates in B-cell differentiation and antiplatelet-antibody production during immune thrombocytopenia. Blood 2014;124(18):2858-66. doi: 10.1182/blood-2014-03-563445 [published Online First: 2014/09/19]

6. Rodeghiero F, Stasi R, Gernsheimer T, et al. Standardization of terminology, definitions and outcome criteria in immune thrombocytopenic purpura of adults and children: report from an international working group. Blood 2009;113(11):2386-93. doi: 10.1182/blood-2008-07-162503 [published Online First: 2008/11/14]

7. Provan D, Stasi R, Newland AC, et al. International consensus report on the investigation and management of primary immune thrombocytopenia. Blood 2010;115(2):168-86. doi: 10.1182/blood-2009-06-225565 [published Online First: 2009/10/23]

8. Brown TM, Horblyuk RV, Grotzinger KM, et al. Patient-reported treatment burden of chronic immune thrombocytopenia therapies. BMC blood disorders 2012;12:2. doi: 10.1186/1471-2326-12-2 [published Online First: 2012/03/23]

9. Newland A et al, poster at the British Society of Haematology meeting 2015

10. Neunert CE. Management of newly diagnosed immune thrombocytopenia: can we change outcomes? Hematology American Society of Hematology Education Program 2017;2017(1):400-05. doi: 10.1182/asheducation-2017.1.400 [published Online First: 2017/12/10]

11. Neunert C, Lim W, Crowther M, et al. The American Society of Hematology 2011 evidence-based practice guideline for immune thrombocytopenia. Blood 2011;117(16):4190-207. doi: 10.1182/blood-2010-08-302984 [published Online First: 2011/02/18]

12. Neunert C, Noroozi N, Norman G, et al. Severe bleeding events in adults and children with primary immune thrombocytopenia: a systematic review. Journal of thrombosis and haemostasis : JTH 2015;13(3):457-64. doi: 10.1111/jth.12813 [published Online First: 2014/12/17]

13. Hill QA, Newland AC. Fatigue in immune thrombocytopenia. British journal of haematology 2015;170(2):141-9. doi: 10.1111/bjh.13385 [published Online First: 2015/04/01]

For peer review only - http://bmjopen.bmj.com/site/about/guidelines.xhtml

14. Snyder CF, Mathias SD, Cella D, et al. Health-related quality of life of immune thrombocytopenic purpura patients: results from a web-based survey. Current medical research and opinion 2008;24(10):2767-76. doi: 10.1185/03007990802377461 [published Online First: 2008/08/22]

15. Tarantino MD, Mathias SD, Snyder CF, et al. Impact of ITP on physician visits and workplace productivity. Current medical research and opinion 2010;26(2):319-28. doi: 10.1185/03007990903451298 [published Online First: 2009/12/09]

16. Kristinsson SY, Gridley G, Hoover RN, et al. Long-term risks after splenectomy among 8,149 cancer-free American veterans: a cohort study with up to 27 years follow-up. Haematologica 2014;99(2):392-8. doi: 10.3324/haematol.2013.092460 [published Online First: 2013/09/24]

17. Rodeghiero F, Ruggeri M. Treatment of immune thrombocytopenia in adults: the role of thrombopoietin-receptor agonists. Seminars in hematology 2015;52(1):16-24. doi: 10.1053/j.seminhematol.2014.10.006 [published Online First: 2015/01/13]

18. Gomez-Almaguer D, Herrera-Rojas MA, Jaime-Perez JC, et al. Eltrombopag and high-dose dexamethasone as frontline treatment of newly diagnosed immune thrombocytopenia in adults. Blood 2014;123(25):3906-8. doi: 10.1182/blood-2014-01-549360 [published Online First: 2014/05/08]

19. Miano M, Ramenghi U, Russo G, et al. Mycophenolate mofetil for the treatment of children with immune thrombocytopenia and Evans syndrome. A retrospective data review from the Italian association of paediatric haematology/oncology. British journal of haematology 2016;175(3):490-95. doi: 10.1111/bjh.14261 [published Online First: 2016/10/28]

20. Taylor A, Neave L, Solanki S, et al. Mycophenolate mofetil therapy for severe immune thrombocytopenia. British journal of haematology 2015;171(4):625-30. doi: 10.1111/bjh.13622 [published Online First: 2015/08/08]

21. Kotb R, Pinganaud C, Trichet C, et al. Efficacy of mycophenolate mofetil in adult refractory auto-immune cytopenias: a single center preliminary study. European journal of haematology 2005;75(1):60-4. doi: 10.1111/j.1600-0609.2005.00437.x [published Online First: 2005/06/11]

22. Provan D, Moss AJ, Newland AC, et al. Efficacy of mycophenolate mofetil as single-agent therapy for refractory immune thrombocytopenic purpura. American journal of hematology 2006;81(1):19-25. doi: 10.1002/ajh.20515 [published Online First: 2005/12/22]

23. Hou M, Peng J, Shi Y, et al. Mycophenolate mofetil (MMF) for the treatment of steroid-resistant idiopathic thrombocytopenic purpura. European journal of haematology 2003;70(6):353-7. [published Online First: 2003/05/21]

24. Colovic M, Suvajdzic N, Colovic N, et al. Mycophenolate mophetil therapy for chronic immune thrombocytopenic purpura resistant to steroids, immunosuppressants, and/or splenectomy in adults. Platelets 2011;22(2):153-6. doi: 10.3109/09537104.2010.520372 [published Online First: 2010/12/15]

25. Zhang WG, Ji L, Cao XM, et al. Mycophenolate mofetil as a treatment for refractory idiopathic thrombocytopenic purpura. Acta pharmacologica Sinica 2005;26(5):598-602. doi: 10.1111/j.1745-7254.2005.00088.x [published Online First: 2005/04/22]

For peer review only - http://bmjopen.bmj.com/site/about/guidelines.xhtml

26. Howard J, Hoffbrand AV, Prentice HG, et al. Mycophenolate mofetil for the treatment of refractory auto-immune haemolytic anaemia and auto-immune thrombocytopenia purpura. British journal of haematology 2002;117(3):712-5. [published Online First: 2002/05/25]

For peer review only

For peer review only - http://bmjopen.bmj.com/site/about/guidelines.xhtml

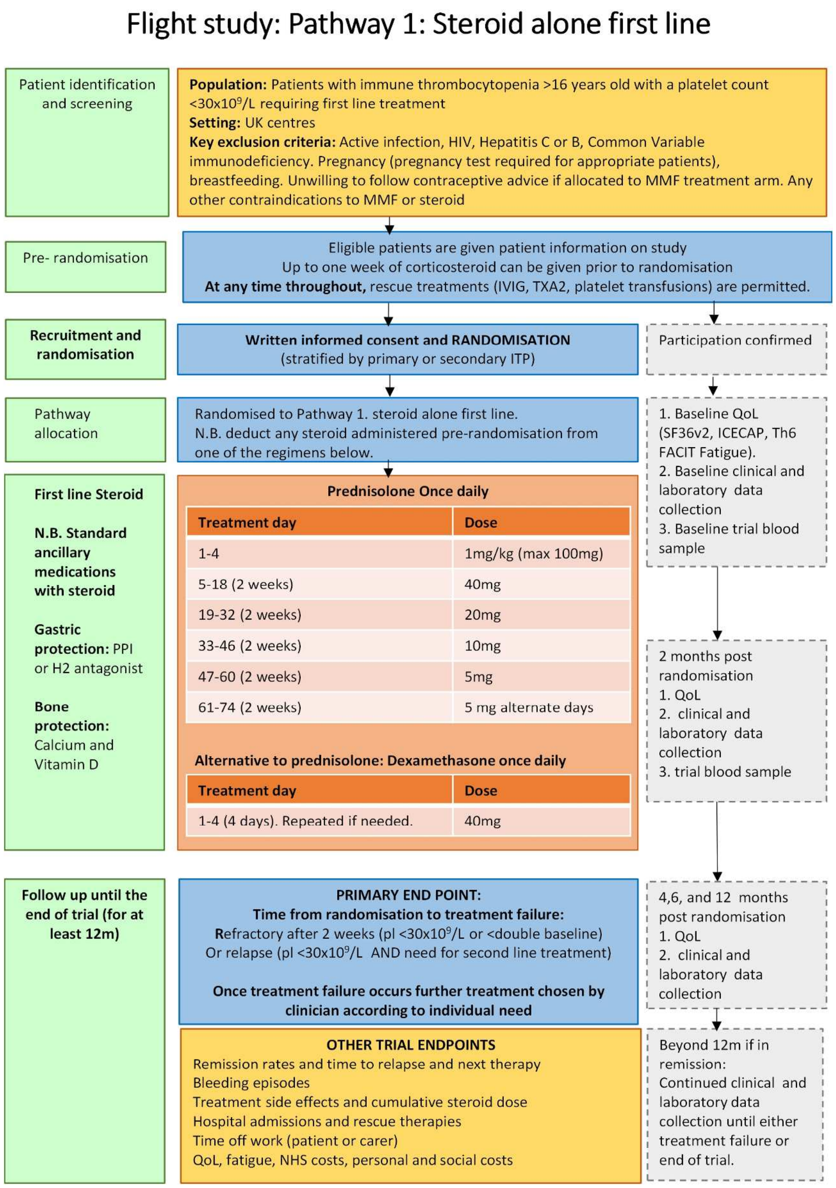

Figure 1. FLIGHT treatment pathway: Corticosteroid only

182x263mm (300 x 300 DPI)

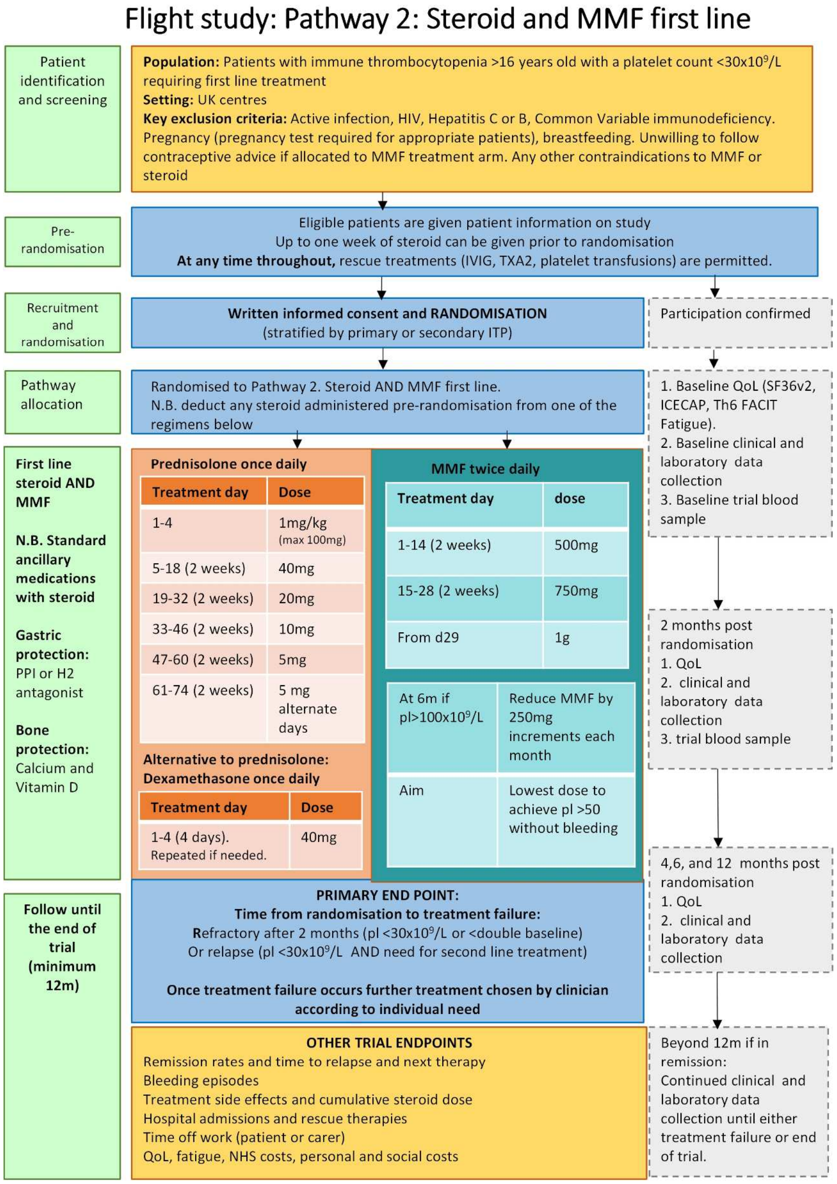

Figure 2. FLIGHT treatment pathway: Corticosteroid and Mycophenolate

185x267mm (300 x 300 DPI)

Reporting checklist for protocol of a clinical trial.

Based on the SPIRIT guidelines.

Instructions to authors

Complete this checklist by entering the page numbers from your manuscript where readers will find each of the items listed below.

Your article may not currently address all the items on the checklist. Please modify your text to include the missing information. If you are certain that an item does not apply, please write "n/a" and provide a short explanation.

Upload your completed checklist as an extra file when you submit to a journal.

In your methods section, say that you used the SPIRIT reporting guidelines, and cite them as:

Chan A-W, Tetzlaff JM, Altman DG, Laupacis A, Gøtzsche PC, Krleža-Jerić K, Hróbjartsson A, Mann H, Dickersin K, Berlin J, Doré C, Parulekar W, Summerskill W, Groves T, Schulz K, Sox H, Rockhold FW, Rennie D, Moher D. SPIRIT 2013 Statement: Defining standard protocol items for clinical trials. Ann Intern Med. 2013;158(3):200-207

		Reporting Item	Page Number
Title	#1	Descriptive title identifying the study design, population, interventions, and, if applicable, trial acronym	1
Trial registration	#2a	Trial identifier and registry name. If not yet registered, name of intended registry	2
Trial registration: data set	#2b	All items from the World Health Organization Trial Registration Data Set	2
Protocol version	#3	Date and version identifier	1
Funding	#4	Sources and types of financial, material, and other support	3
Roles and responsibilities: contributorship	#5a	Names, affiliations, and roles of protocol contributors	1 and 13
Roles and	#5b	Name and contact information for the trial sponsor	1

For peer review only

responsibilities: sponsor contact information			
Roles and responsibilities: sponsor and funder	#5c	Role of study sponsor and funders, if any, in study design; collection, management, analysis, and interpretation of data; writing of the report; and the decision to submit the report for publication, including whether they will have ultimate authority over any of these activities	Supplementary
Roles and responsibilities: committees	#5d	Composition, roles, and responsibilities of the coordinating centre, steering committee, endpoint adjudication committee, data management team, and other individuals or groups overseeing the trial, if applicable (see Item 21a for data monitoring committee)	Supplementary
Background and rationale	#6a	Description of research question and justification for undertaking the trial, including summary of relevant studies (published and unpublished) examining benefits and harms for each intervention	3-6
Background and rationale: choice of comparators	#6b	Explanation for choice of comparators	3-6
Objectives	#7	Specific objectives or hypotheses	6-7
Trial design	#8	Description of trial design including type of trial (eg, parallel group, crossover, factorial, single group), allocation ratio, and framework (eg, superiority, equivalence, non-inferiority, exploratory)	7
Study setting	#9	Description of study settings (eg, community clinic, academic hospital) and list of countries where data will be collected. Reference to where list of study sites can be obtained	8
Eligibility criteria	#10	Inclusion and exclusion criteria for participants. If applicable, eligibility criteria for study centres and individuals who will perform the interventions (eg, surgeons, psychotherapists)	7

For peer review only

Interventions: description	#11a	Interventions for each group with sufficient detail to allow replication, including how and when they will be administered	8, 9 and figure 1
Interventions: modifications	#11b	Criteria for discontinuing or modifying allocated interventions for a given trial participant (eg, drug dose change in response to harms, participant request, or improving / worsening disease)	Supplementary
Interventions: adherance	#11c	Strategies to improve adherence to intervention protocols, and any procedures for monitoring adherence (eg, drug tablet return; laboratory tests)	Supplementary
Interventions: concomitant care	#11d	Relevant concomitant care and interventions that are permitted or prohibited during the trial	8,9 and figure 1
Outcomes	#12	Primary, secondary, and other outcomes, including the specific measurement variable (eg, systolic blood pressure), analysis metric (eg, change from baseline, final value, time to event), method of aggregation (eg, median, proportion), and time point for each outcome. Explanation of the clinical relevance of chosen efficacy and harm outcomes is strongly recommended	9,10
Participant timeline	#13	Time schedule of enrolment, interventions (including any run-ins and washouts), assessments, and visits for participants. A schematic diagram is highly recommended (see Figure)	Table 1
Sample size	#14	Estimated number of participants needed to achieve study objectives and how it was determined, including clinical and statistical assumptions supporting any sample size calculations	11
Recruitment	#15	Strategies for achieving adequate participant enrolment to reach target sample size	11
Allocation: sequence generation	#16a	Method of generating the allocation sequence (eg, computer-generated random numbers), and list of any factors for stratification. To reduce predictability of a random sequence, details of any planned restriction (eg, blocking) should be provided in a	8

For peer review only

		separate document that is unavailable to those who enrol participants or assign interventions	
Allocation concealment mechanism	#16b	Mechanism of implementing the allocation sequence (eg, central telephone; sequentially numbered, opaque, sealed envelopes), describing any steps to conceal the sequence until interventions are assigned	8
Allocation: implementation	#16c	Who will generate the allocation sequence, who will enrol participants, and who will assign participants to interventions	8
Blinding (masking)	#17a	Who will be blinded after assignment to interventions (eg, trial participants, care providers, outcome assessors, data analysts), and how	N/A
Blinding (masking): emergency unblinding	#17b	If blinded, circumstances under which unblinding is permissible, and procedure for revealing a participant's allocated intervention during the trial	N/A
Data collection plan	#18a	Plans for assessment and collection of outcome, baseline, and other trial data, including any related processes to promote data quality (eg, duplicate measurements, training of assessors) and a description of study instruments (eg, questionnaires, laboratory tests) along with their reliability and validity, if known. Reference to where data collection forms can be found, if not in the protocol	10 and table 1
Data collection plan: retention	#18b	Plans to promote participant retention and complete follow-up, including list of any outcome data to be collected for participants who discontinue or deviate from intervention protocols	Supplementary
Data management	#19	Plans for data entry, coding, security, and storage, including any related processes to promote data quality (eg, double data entry; range checks for data values). Reference to where details of data management procedures can be found, if not in the protocol	10 and Supplementary
Statistics: outcomes	#20a	Statistical methods for analysing primary and secondary outcomes. Reference to where other	11,12

For peer review only

		details of the statistical analysis plan can be found, if not in the protocol	
Statistics: additional analyses	#20b	Methods for any additional analyses (eg, subgroup and adjusted analyses)	NA
Statistics: analysis population and missing data	#20c	Definition of analysis population relating to protocol non-adherence (eg, as randomised analysis), and any statistical methods to handle missing data (eg, multiple imputation)	Supplementary
Data monitoring: formal committee	#21a	Composition of data monitoring committee (DMC); summary of its role and reporting structure; statement of whether it is independent from the sponsor and competing interests; and reference to where further details about its charter can be found, if not in the protocol. Alternatively, an explanation of why a DMC is not needed	Supplementary
Data monitoring: interim analysis	#21b	Description of any interim analyses and stopping guidelines, including who will have access to these interim results and make the final decision to terminate the trial	12
Harms	#22	Plans for collecting, assessing, reporting, and managing solicited and spontaneously reported adverse events and other unintended effects of trial interventions or trial conduct	Supplementary
Auditing	#23	Frequency and procedures for auditing trial conduct, if any, and whether the process will be independent from investigators and the sponsor	Supplementary
Research ethics approval	#24	Plans for seeking research ethics committee / institutional review board (REC / IRB) approval	2
Protocol amendments	#25	Plans for communicating important protocol modifications (eg, changes to eligibility criteria, outcomes, analyses) to relevant parties (eg, investigators, REC / IRBs, trial participants, trial registries, journals, regulators)	Supplementary
Consent or assent	#26a	Who will obtain informed consent or assent from potential trial participants or authorised surrogates,	7 and Supplementary

For peer review only

		and how (see Item 32)	
Consent or assent: ancillary studies	#26b	Additional consent provisions for collection and use of participant data and biological specimens in ancillary studies, if applicable	10 and Supplementary
Confidentiality	#27	How personal information about potential and enrolled participants will be collected, shared, and maintained in order to protect confidentiality before, during, and after the trial	Supplementary
Declaration of interests	#28	Financial and other competing interests for principal investigators for the overall trial and each study site	3
Data access	#29	Statement of who will have access to the final trial dataset, and disclosure of contractual agreements that limit such access for investigators	Supplementary
Ancillary and post trial care	#30	Provisions, if any, for ancillary and post-trial care, and for compensation to those who suffer harm from trial participation	Supplementary
Dissemination policy: trial results	#31a	Plans for investigators and sponsor to communicate trial results to participants, healthcare professionals, the public, and other relevant groups (eg, via publication, reporting in results databases, or other data sharing arrangements), including any publication restrictions	2
Dissemination policy: authorship	#31b	Authorship eligibility guidelines and any intended use of professional writers	NA
Dissemination policy: reproducible research	#31c	Plans, if any, for granting public access to the full protocol, participant-level dataset, and statistical code	Supplementary
Informed consent materials	#32	Model consent form and other related documentation given to participants and authorised surrogates	Supplementary
Biological specimens	#33	Plans for collection, laboratory evaluation, and storage of biological specimens for genetic or molecular analysis in the current trial and for future use in ancillary studies, if applicable	Supplementary

For peer review only

The SPIRIT checklist is distributed under the terms of the Creative Commons Attribution License CC-BY-ND 3.0. This checklist can be completed online using https://www.goodreports.org/, a tool made by the EQUATOR Network in collaboration with Penelope.ai

For peer review only
